# Supplementary material for: Structural basis of NSD2 degradation via targeted recruitment of SCF-FBXO22
Source: Nat Commun. 2026 Apr 23;17:5635. doi: 10.1038/s41467-026-72235-9 (PMC13316043; doi:10.1038/s41467-026-72235-9)
Supplement: Supplementary file 1 — Supplementary Information [file 41467_2026_72235_MOESM1_ESM.pdf]

Supplementary Information

Structural basis of NSD2 degradation via targeted recruitment of SCF-FBXO22

Kevin C. Robertson[#], Sascha J. Amann[#], Tongkun Liu[#], Adam V. Funk, Xianxi Wang, Irina Grishkovskaya, Aamir Mehmood, John R. Tabor, Jacqueline L. Norris-Drouin, Cheryl H. Arrowsmith, Jon L. Collins, Yinglong Miao, Michael J. Emanuele, David Haselbach*, Lindsey I. James*, Nicholas G. Brown*

[#]These authors contributed equally: K.C.R., S.J.A., and T.L.

*Correspondence should be addressed to D.H. (david.haselbach@imp.ac.at), L.I.J. (ingerman@email.unc.edu), or N.G.B. (nbrown1@med.unc.edu)

Table of Contents

P2-P3 Supplementary Table 1
P4-P5 Supplementary Fig. 1
P6 Supplementary Fig. 2
P7-P8 Supplementary Fig. 3
P9-P10 Supplementary Fig. 4
P11-P12 Supplementary Fig. 5
P13 Supplementary Fig. 6
P14-P15 Supplementary Fig. 7
P16-P17 Supplementary Fig. 8
P18 Supplementary Fig. 9
P19 General Chemistry Procedures
P19-P20 Nuclear Magnetic Resonance Spectroscopy (NMR)
P20-P21 Abbreviations Used
P21-P41 Synthetic Procedures
P42-P65 NMR Spectra
P66-P79 LC-MS Spectra
P80-P82 MD Simulation Checklist

Supplementary Table 1. Cryo-EM data collection, refinement, and validation statistics.

	SCF^{FBXO22}, NSD2, UNC10088	SCF^{FBXO22}, NSD2, UNC10415667	SCF^{FBXO22}, NSD2, BACH1, UNC10088
EMDB ID	(EMD-57178)	(EMD-57180)	(EMD-57179)
PDB ID	(PDB 29HG)	(PDB 29HI)	(PDB 29HH)
**Data collection and processing**			
Magnification	130k x		
Voltage (kV)	300		
Electron exposure (e–/Åˆ2)	40		
Detector	Thermo Fisher Falcon 4i		
Defocus range (μm)	-1.0 to -2.0 μm (0.2 μm increments)		
Pixel size (Å)	0.951		
Symmetry imposed	C1		
Initial particle images (no.)	17,831,444	11,321,391	4,280,116
Final particle images (no.)	207,711	47,442	73,358
Map sharpening B factor (Å^2)	n/a	n/a	n/a
Map resolution range (Å)	3.2-4.0	5.1-5.8	4.0-4.2
FSC threshold	0.143		
**Model Refinement**			
Initial model used	AF3, PDB: 6UE6	AF3, PDB: 6UE6	AF3, PDB: 6UE6, 8UA3
Model resolution (Å)	6.0	7.1	7.6
FSC threshold	0.5		
**Model composition**			
Non-hydrogen atoms	12,386	12,388	14,320
Protein residues	1540	1540	1783
Ligands	1	1	1
**B factors**			
Protein (Å^2) min/max/mean	66.6/310.5/149.6	142.1/669.2/284.6	68.7/389.4/174.8
Ligand (Å^2)	154.2	337.1	199.2
**R.m.s. deviations**			
Bond lengths (Å)	0.005	0.005	0.005
Bond angles (°)	1.119	1.238	0.984

Validation			
MolProbity score	2.28	2.18	2.09
Clashscore	12.41	16.67	15.5
Poor rotamers (%)	2.07	0.07	0.13
**Ramachandran plot**			
Favored (%)	93.37	92.78	94.15
Allowed (%)	6.63	7.22	5.73
Disallowed (%)	0.00	0.00	0.11

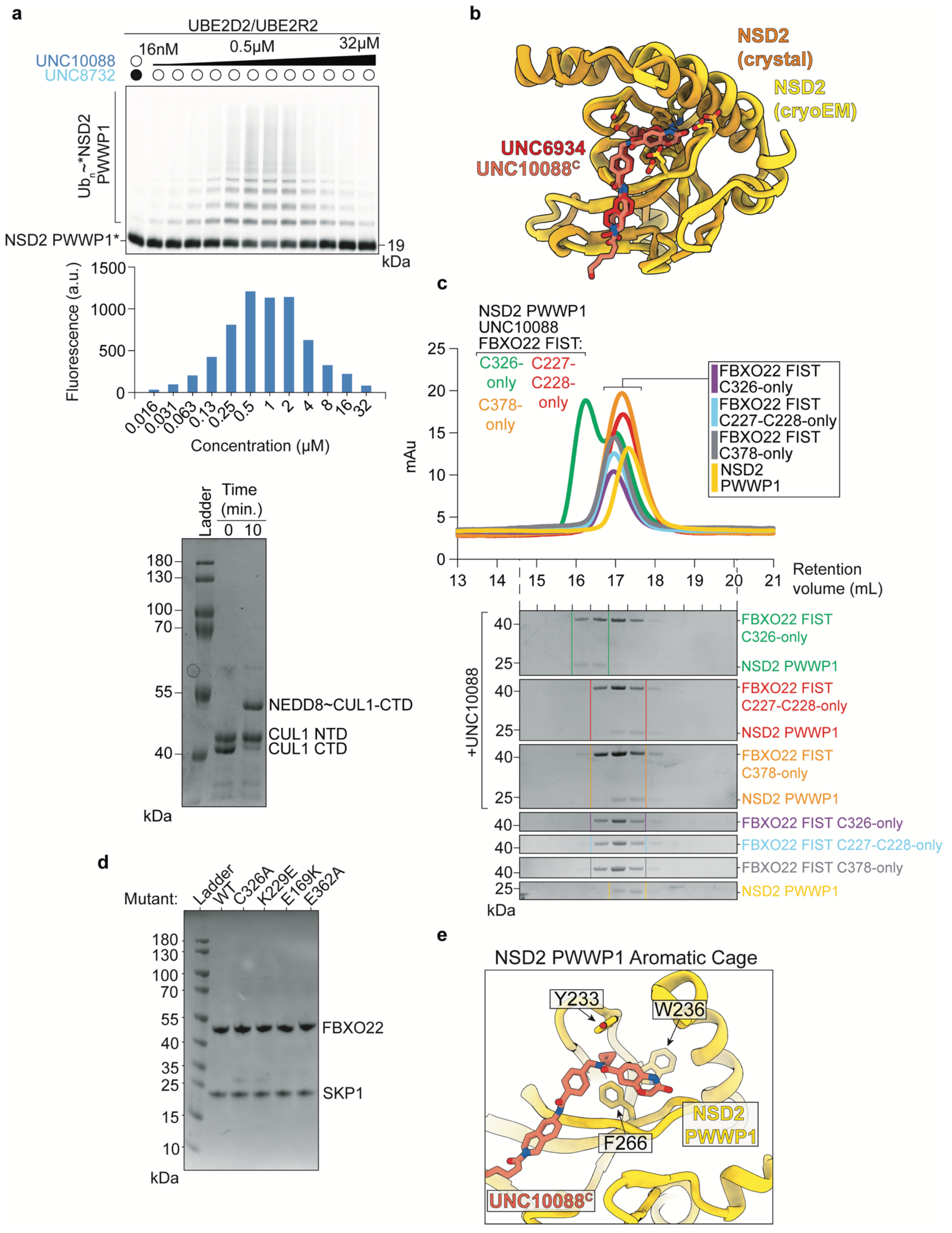
a
UBE2D2/UBE2R2
16nM
0.5μM
32μM
UNC10088
UNC8732
Ubn~*NSD2 PWWP1
NSD2 PWWP1*
19
kDa
Fluorescence (a.u.)
1500
1000
500
0
0.016
0.031
0.063
0.13
0.25
0.5
1
2
4
8
16
32
Concentration (μM)
Ladder
Time (min.)
0
10
180
130
100
70
55
40
kDa
NEDD8~CUL1-CTD
CUL1 NTD
CUL1 CTD
b
NSD2 (crystal)
NSD2 (cryoEM)
UNC6934
UNC10088c
c
NSD2 PWWP1
UNC10088
FBXO22 FIST:
C326-only
C227-C228-only
C378-only
mAu
25
20
15
10
5
0
13
14
15
16
17
18
19
20
21
Retention volume (mL)
FBXO22 FIST C326-only
FBXO22 FIST C227-C228-only
FBXO22 FIST C378-only
NSD2 PWWP1
+UNC10088
40
25
FBXO22 FIST C326-only
NSD2 PWWP1
FBXO22 FIST C227-C228-only
NSD2 PWWP1
FBXO22 FIST C378-only
NSD2 PWWP1
FBXO22 FIST C326-only
FBXO22 FIST C227-C228-only
FBXO22 FIST C378-only
NSD2 PWWP1
kDa
d
Mutant:
Ladder
WT
C326A
K229E
E169K
E362A
180
130
100
70
55
40
35
25
15
10
kDa
FBXO22
SKP1
e
NSD2 PWWP1 Aromatic Cage
Y233
W236
F266
NSD2 PWWP1
UNC10088c

Supplementary Fig. 1 | Structural comparisons using the SCF^{FBXO22}-UNC10088^{C}-NSD2 PWWP1 cryo-EM model reveal UNC10088^{C} occupies a similar orientation relative to previous structures and representative gels related to ubiquitination assays. a) Fluorescent monitoring of an SDS-PAGE gel (top) and quantified fluorescence from ubiquitination of NSD2 (middle), demonstrating an inhibitory hook effect upon titration of UNC10088 in SCF^{FBXO22}-degrader-dependent ubiquitination. Representative image of n=2 independent experiments. Fluorescence readout was quantified by performing a background subtraction of the negative control fluorescence (0.5 μM of UNC8732) from each condition. Coomassie-stained SDS-PAGE gel of a neddylation reaction using the Split 'n' Co-express version of full-length CUL1-RBX1 (bottom). Representative image of n=3 independent experiments. **b)** Structural comparison of SCF^{FBXO22}-UNC10088^{C}-NSD2 PWWP1 complex and the prior NSD2 crystal structure bound to the ligand used to derive UNC10088 (UNC6934) and similar molecules for FBXO22-mediated degradation[20,38]. **c)** Gel filtration profiles of individual proteins (NSD2 PWWP1 (yellow), FBXO22 FIST Cys 326-only (purple), Cys227-C228-only (blue), and Cys378-only (gray)) and protein pairs (NSD2 PWWP1 and UNC10088 added to either FBXO22 FIST Cys326-only (green), Cys227-C228-only (red), or Cys378 (orange)), revealing that only C326 can facilitate ternary complex formation. Data are representative of an independent experiment. **d)** SDS-PAGE gel of SKP1-FBXO22 WT and variants tested in Figure 2B. normalized to 5 μM. Proteins bands were visualized using Coomassie stain. **e)** Aromatic cage of NSD2 for ligand recruitment. Source data are provided as a source data file.

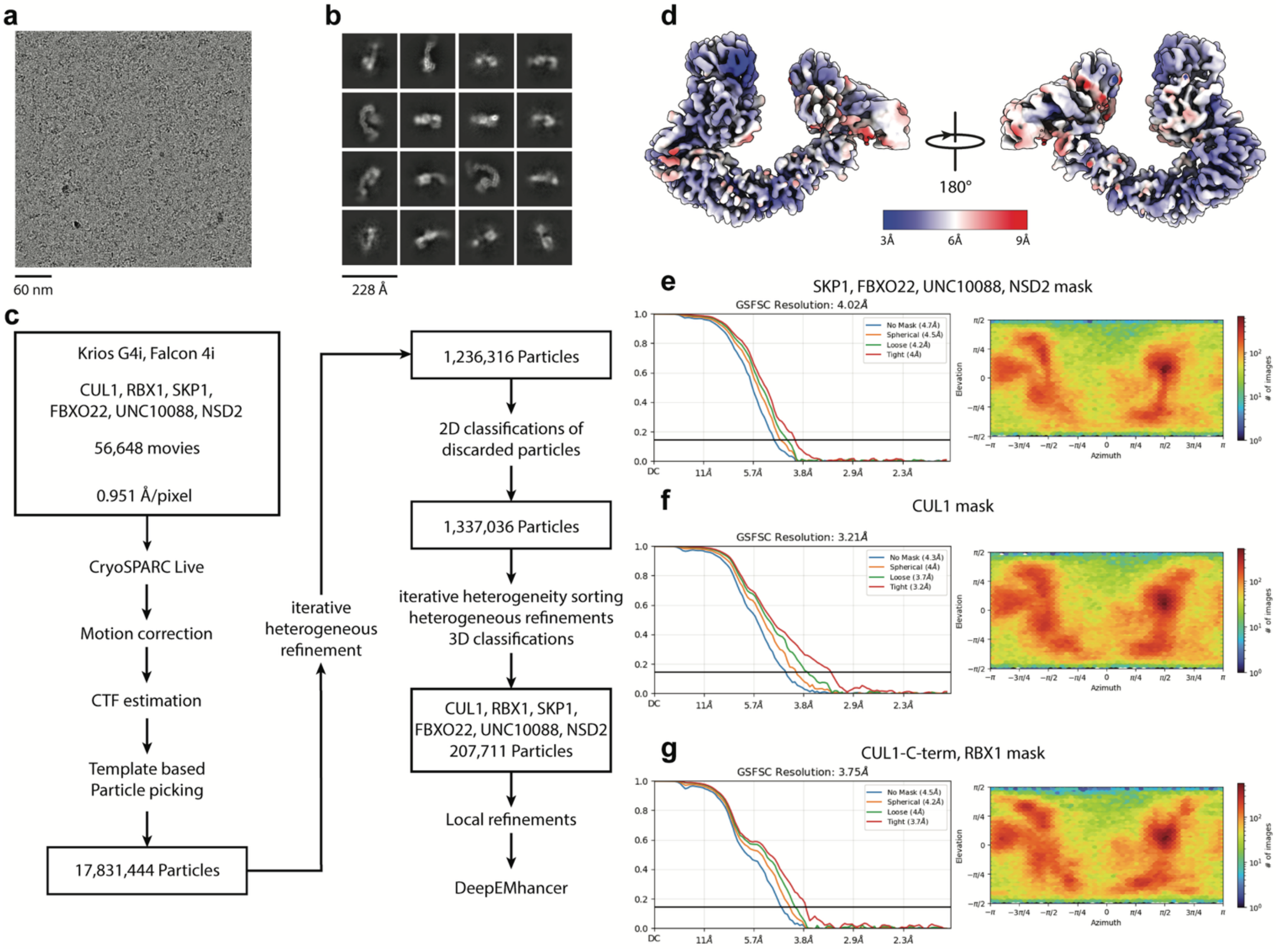

Supplementary Fig. 2 | Cryo-EM analysis of the SCF^{FBXO22}-$UNC10088^{C}$-NSD2 PWWP1 complex. a) Representative micrograph. **b)** Representative 2D class averages. **c)** Processing pipeline. **d)** Local resolution estimation of local refined map. **e-g)** GSFSC curves of local refined map and viewing angle distributions.

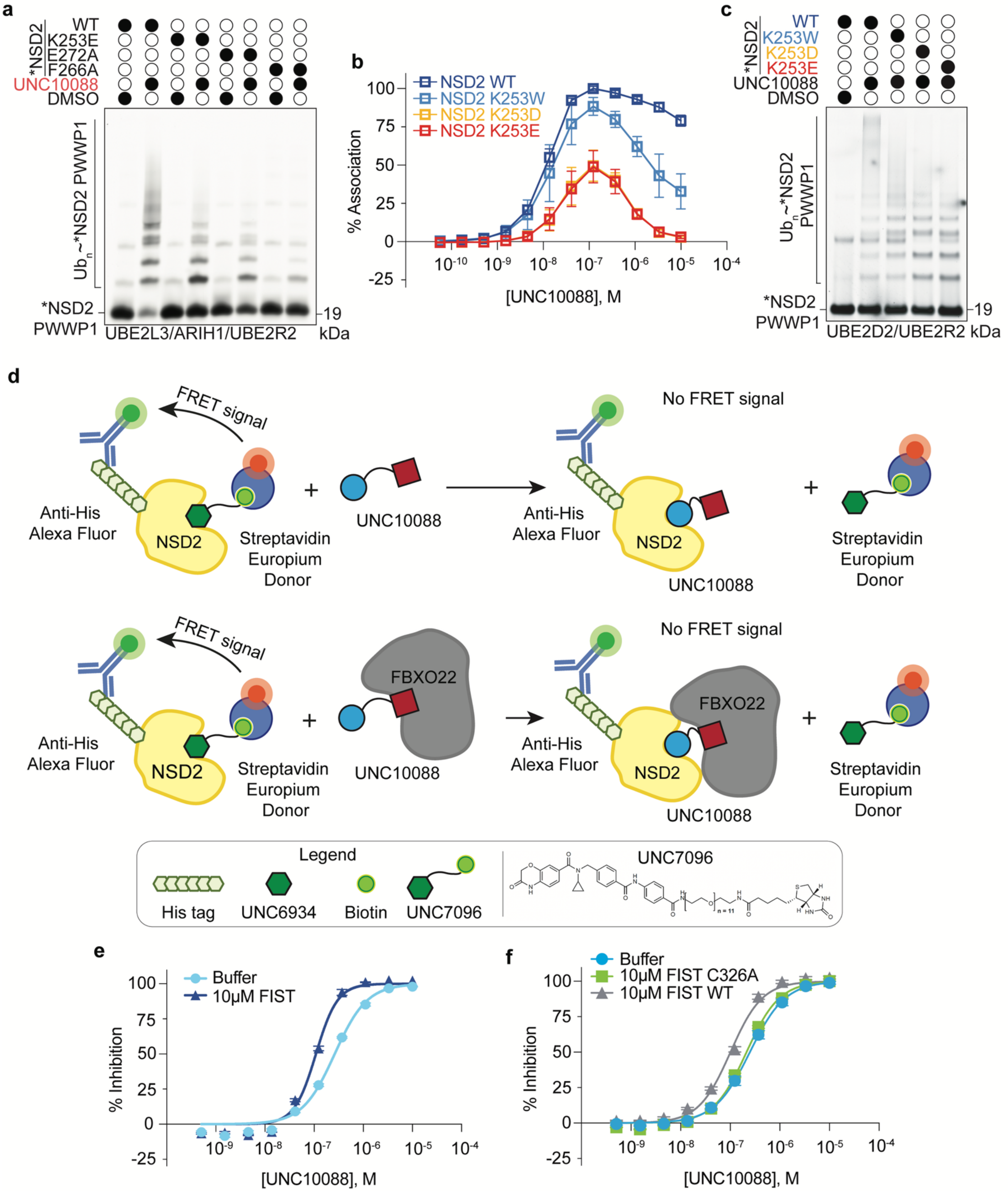

a
*NSD2
WT
K253E
E272A
F266A
UNC10088
DMSO
Ubn~*NSD2 PWWP1
*NSD2 PWWP1
19
UBE2L3/ARIH1/UBE2R2
kDa
b
NSD2 WT
NSD2 K253W
NSD2 K253D
NSD2 K253E
% Association
[UNC10088], M
c
*NSD2
WT
K253W
K253D
K253E
UNC10088
DMSO
Ubn~*NSD2 PWWP1
*NSD2 PWWP1
19
UBE2D2/UBE2R2 kDa
d
FRET signal
No FRET signal
Anti-His Alexa Fluor
NSD2
Streptavidin Europium Donor
UNC10088
FBXO22
Legend
His tag
UNC6934
Biotin
UNC7096
e
Buffer
10μM FIST
% Inhibition
[UNC10088], M
f
Buffer
10μM FIST C326A
10μM FIST WT
% Inhibition
[UNC10088], M

Supplementary Fig. 3 | NSD2 variants disrupt ternary complex formation and cooperative inhibition using TR-FRET. a) Similar to Fig. 2C, fluorescent monitoring of an SDS-PAGE gel comparing the effects of indicated substitutions on *NSD2 PWWP1 ubiquitination by SCF^{FBXO22} and UNC10088 using UBE2L3, ARIH1, and UBE2R2 as the UCEs. Representative image of n=2 independent experiments. **b)** His-tagged K253 variants of NSD2 disrupt complex association with biotinylated FBXO22 FIST as monitored by a UNC10088 titration in a TR-FRET assay. Data are presented as mean values of n=3 independent experiments. Error bars: standard deviation. **c)** Fluorescent monitoring of an SDS-PAGE gel demonstrating that K253 variants of NSD2 disrupt SCF^{FBXO22}-UNC10088-mediated ubiquitination at similar levels to their disruption of complex binding shown in b. Representative image of an independent experiment. **d)** Cartoon model of TR-FRET competition assay monitoring the interaction between His-tagged NSD2 PWWP1 and a biotinylated version of the NSD2 PWWP1 ligand (UNC7096), which is disrupted by the addition of UNC10088 in the presence or absence of FBXO22 FIST. **e)** Similar to Figure 2H, but the reagents were not preincubated. Data are presented as mean values of three technical replicates of an independent experiment. Error bars: standard error of the mean. **f)** Competitive inhibition of the TR-FRET signal upon addition of UNC10088 in the presence of buffer (IC_{50} = 248 ± 22 nM), WT FIST (IC_{50} = 101 ± 8 nM), or C326A FIST (IC_{50} = 227 ± 10 nM). Data and curve fits of UNC10088 titrations with or without the FBXO22 FIST domain showing cooperative binding are repeated from Fig 2H for comparison with the FIST domain harboring the C326A variant. Data are presented as mean values of n=2 independent experiments. Error bars: standard error of the mean. Source data are provided as a source data file.

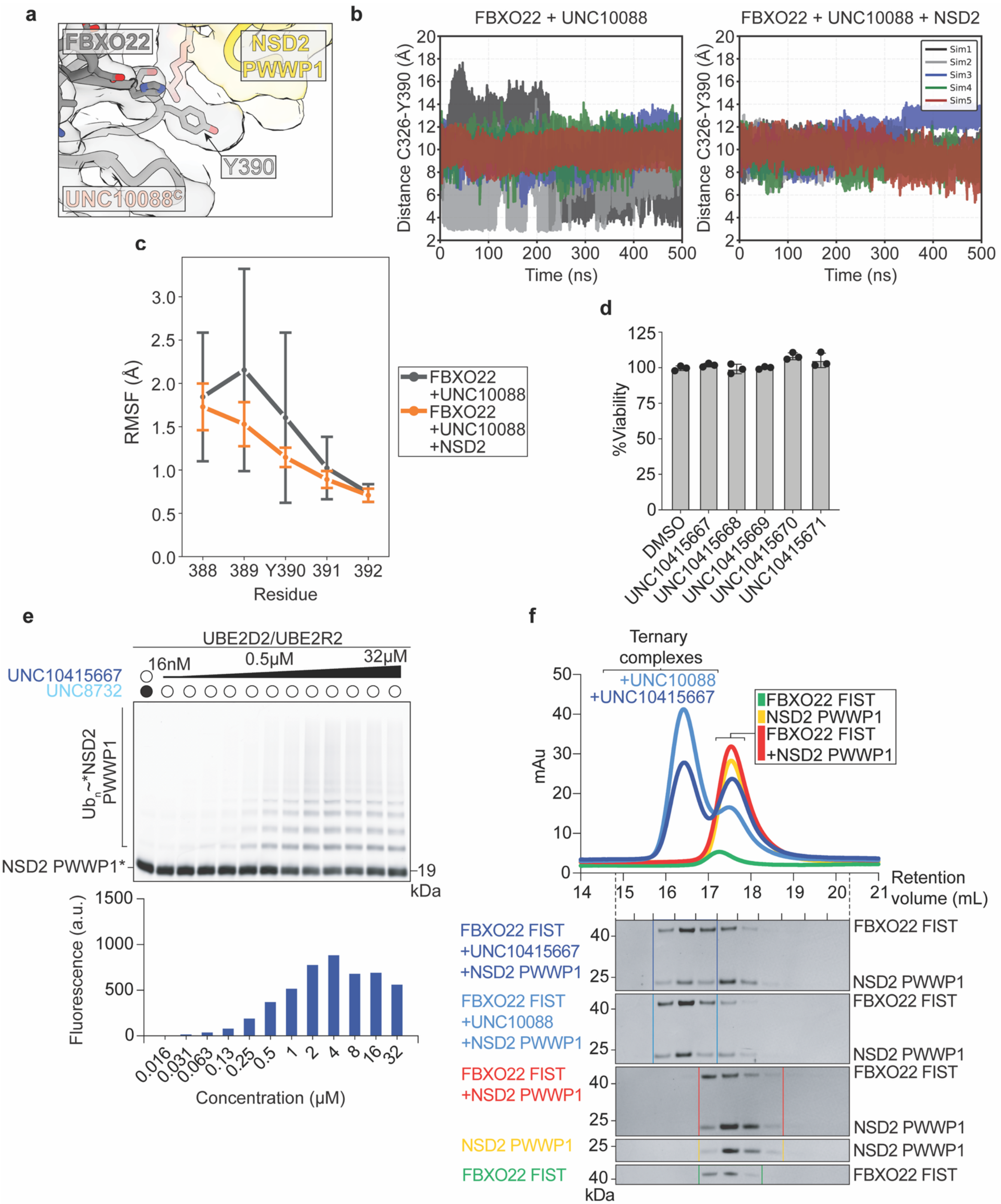
a
FBXO22
NSD2 PWWP1
Y390
UNC10088C
b
FBXO22 + UNC10088
FBXO22 + UNC10088 + NSD2
Distance C326-Y390 (Å)
Time (ns)
Sim1
Sim2
Sim3
Sim4
Sim5
c
RMSF (Å)
Residue
388
389
Y390
391
392
FBXO22 +UNC10088
FBXO22 +UNC10088 +NSD2
d
%Viability
DMSO
UNC10415667
UNC10415668
UNC10415669
UNC10415670
UNC10415671
e
UBE2D2/UBE2R2
16nM
0.5μM
32μM
UNC10415667
UNC8732
Ubn~*NSD2 PWWP1
NSD2 PWWP1*
19 kDa
Fluorescence (a.u.)
Concentration (μM)
f
Ternary complexes
+UNC10088
+UNC10415667
FBXO22 FIST
NSD2 PWWP1
FBXO22 FIST +NSD2 PWWP1
mAu
Retention volume (mL)
FBXO22 FIST +UNC10415667 +NSD2 PWWP1
FBXO22 FIST +UNC10088 +NSD2 PWWP1
FBXO22 FIST +NSD2 PWWP1
NSD2 PWWP1
FBXO22 FIST
kDa

Supplementary Fig. 4 | Y390-containing loop flexibility and characterization of UNC10415667. a) Model and related density of Y390 in the SCF^{FBXO22}-UNC10088^{C}-NSD2 PWWP1 complex. **b)** GaMD simulation trajectories reveal that the distance between C326 and Y390 remains highly stable in the presence of NSD2, with minimal fluctuations observed when compared to simulations in the absence of NSD2. **c)** RMSF analysis of the Y390-containing region reveals significantly reduced residue-level fluctuations for Y390 and its neighboring residues in the presence of NSD2 (orange) when compared to simulations in the absence of NSD2 (gray). These observations support the role of NSD2 in stabilizing the structural integrity of the complex and this loop. **d)** Representative bar graph of percent (%) cell viability of U2OS cells treated with benzaldehyde derivatives of UNC8732 from Fig 3B. Error bars: standard deviation. **e)** Fluorescent monitoring of an SDS-PAGE gel (top) and quantified fluorescence from ubiquitination of NSD2 (bottom) demonstrating an inhibitory hook effect upon titration of UNC10415667 in SCF^{FBXO22}-degrader-dependent ubiquitination. Fluorescence readout is quantified by performing a background subtraction of the negative control fluorescence (0.5 μM of UNC8732) from each condition. Representative image of n=2 independent experiments. **f)** UNC10088- and UNC10415667-mediated FBXO22 FIST-NSD2 PWWP1 ternary complex formation, similar to Fig. 2A. Gel filtration chromatography elution profiles of FBXO22 FIST wild-type alone or mixed with NSD2 and either UNC10088 or UNC10415667. Source data are provided as a source data file.

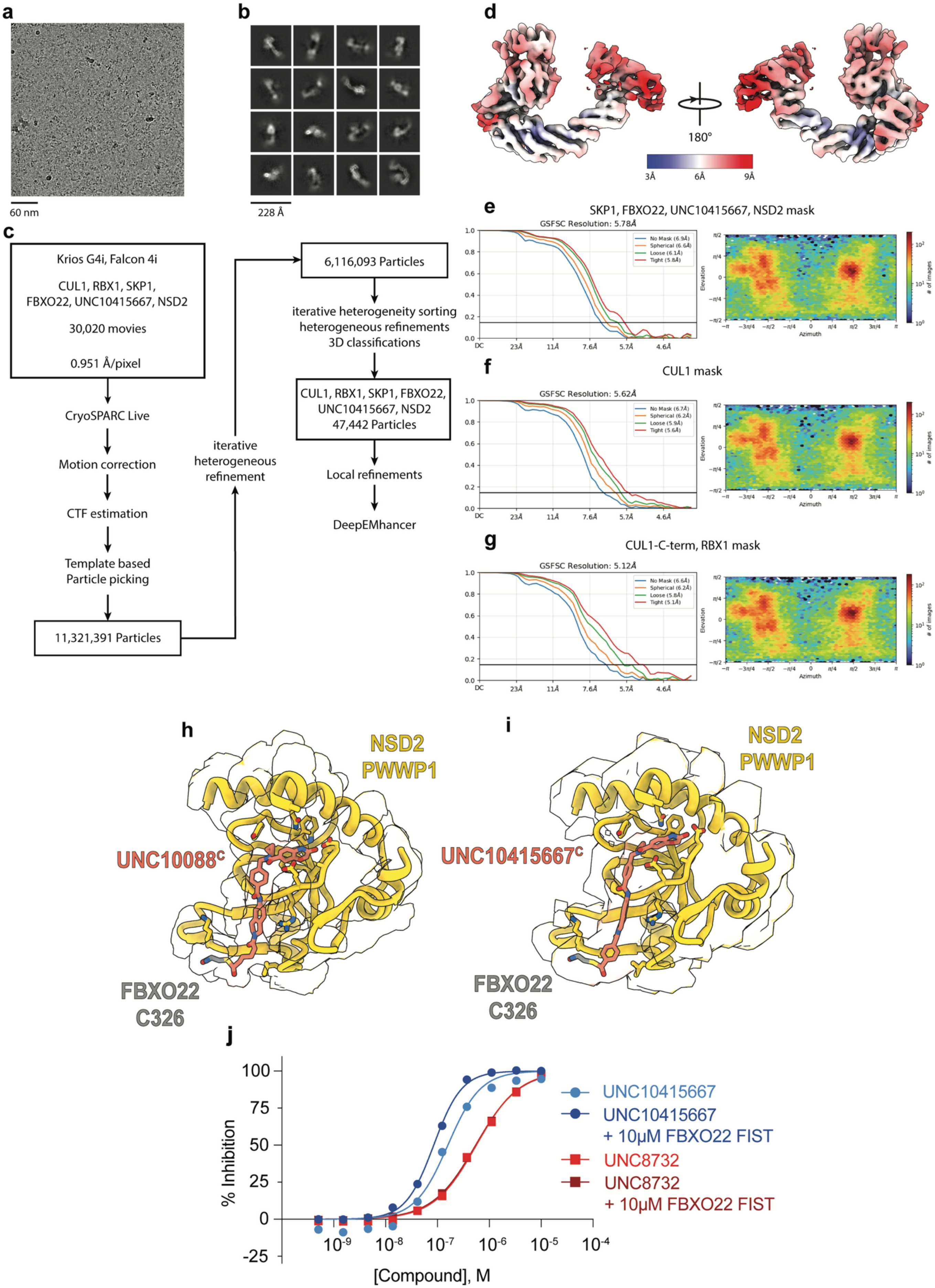

a
60 nm
b
228 Å
c
Krios G4i, Falcon 4i
CUL1, RBX1, SKP1, FBXO22, UNC10415667, NSD2
30,020 movies
0.951 Å/pixel
CryoSPARC Live
Motion correction
CTF estimation
Template based Particle picking
11,321,391 Particles
iterative heterogeneous refinement
6,116,093 Particles
iterative heterogeneity sorting
heterogeneous refinements
3D classifications
CUL1, RBX1, SKP1, FBXO22, UNC10415667, NSD2
47,442 Particles
Local refinements
DeepEMhancer
d
180°
3Å
6Å
9Å
e
SKP1, FBXO22, UNC10415667, NSD2 mask
GSFSC Resolution: 5.78Å
No Mask (6.9Å)
Spherical (6.6Å)
Loose (6.1Å)
Tight (5.8Å)
f
CUL1 mask
GSFSC Resolution: 5.62Å
No Mask (6.7Å)
Spherical (6.2Å)
Loose (5.9Å)
Tight (5.6Å)
g
CUL1-C-term, RBX1 mask
GSFSC Resolution: 5.12Å
No Mask (6.6Å)
Spherical (6.2Å)
Loose (5.8Å)
Tight (5.1Å)
Elevation
Azimuth
# of images
h
NSD2 PWWP1
UNC10088^C
FBXO22 C326
i
NSD2 PWWP1
UNC10415667^C
FBXO22 C326
j
% Inhibition
[Compound], M
UNC10415667
UNC10415667 + 10μM FBXO22 FIST
UNC8732
UNC8732 + 10μM FBXO22 FIST

Supplementary Fig. 5 | Cryo-EM analysis of the SCF^{FBXO22}-UNC10415667^{C}-NSD2 PWWP1 complex and additional assays with UNC10415667 and its derivatives. a) Representative micrograph. **b)** Representative 2D class averages. **c)** Processing pipeline. **d)** Local resolution estimation of local refined map. **e-g)** GSFSC curves of local refined map and viewing angle distributions. **h)** Close-up view of UNC10088^{C} (orange) recruitment of NSD2 PWWP1 (yellow) to FBXO22 via FBXO22 C326 (gray). **i)** Close-up view of UNC10415667^{C} (orange) recruitment of NSD2 PWWP1 (yellow) to FBXO22 via FBXO22 C326 (gray). **j)** Competitive inhibition of the TR-FRET signal upon addition of UNC10415667 (IC_{50} = 159 ± 24 nM), UNC10415667 in the presence of FBXO22 FIST (IC_{50} = 85 ± 4 nM), or UNC8732 in the presence or absence of FBXO22 FIST (IC_{50} ~ 550 nM), similar to Fig. 2H. Curve fits of UNC8732 titrations with or without the FBXO22 FIST domain are repeated from Fig 2H for comparison. Error bars: standard error of the mean. Source data are provided as a source data file.

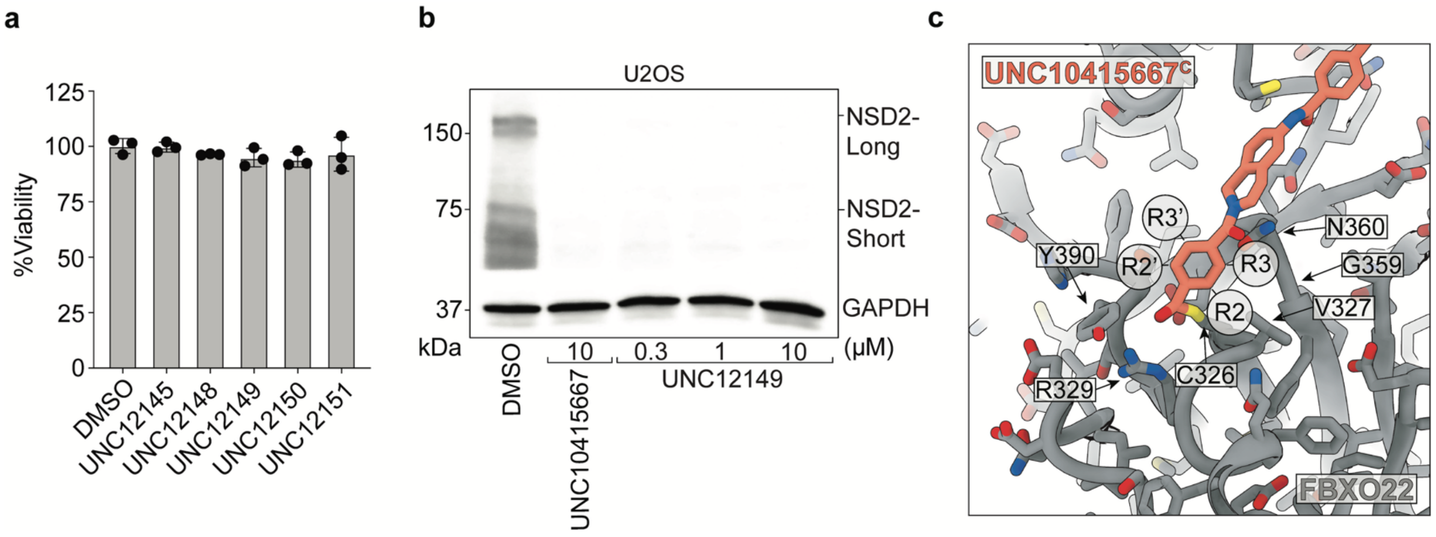

Supplementary Fig. 6 | Validation of UNC10415667-, UNC12149-mediated NSD2 degradation in cells and structural modeling. a) Representative bar graph of percent (%) cell viability of U2OS cells treated with benzaldehyde derivatives inspired by UNC10415667. Error bars: standard deviation. **b)** Immunoblot of NSD2 degradation in U2OS cells after 6 hr treatment with UNC10415667 or UNC12149 versus DMSO, supporting NSD2 degradation from HiBit data. GAPDH is used as a loading control. **c)** Close-up view of SCF^{FBXO22}-UNC10415667-NSD2 structure highlighting the possible R2 (R2') and R3 (R3') positions (Figure 4E), depending on the orientation of benzene ring, and neighboring FBXO22 residues. Source data are provided as a source data file.

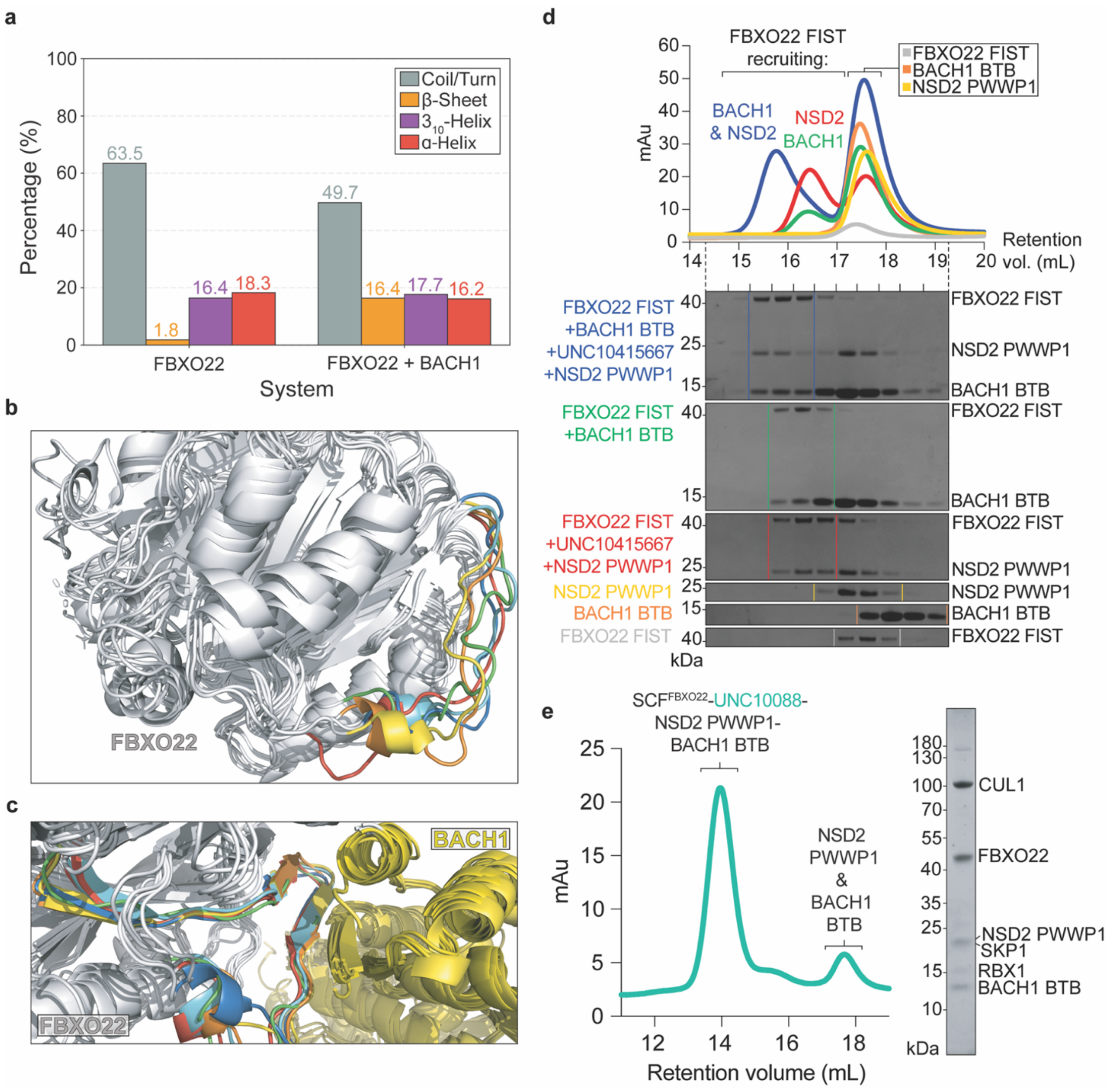

Supplementary Fig. 7 | Formation of the SCF^{FBXO22}-UNC10088^{C}-NSD2 PWWP1-BACH1 BTB complex for structural studies. a-c) Secondary structure analysis of the FBXO22 loop interfacing with BACH1 reveals pronounced conformational changes that occur specifically upon BACH1 binding. In the absence of BACH1, the loop remains structurally unchanged (a-b). However, when BACH1 is present, the interacting loop undergoes a clear transition marked by the formation of β-sheet elements, a trend consistently observed across all five independent simulations (a,c). **d)** Gel filtration profiles (top) of individual proteins (FBXO22 FIST (gray), NSD2 (yellow), BACH1(orange)), subcomplexes (FBXO22 FIST-UNC10415667-NSD2 (red) or FBXO22 FIST-BACH1 BTB (green)), or FBXO22 FIST-UNC10415667-NSD2-BACH1 (blue). Representative Coomassie-stained SDS-PAGE gels of sizing fractions (bottom). Filtration profiles

from Figure 5A are repeated here for clarity. Data are representative of n=2 independent experiments. **e)** Gel filtration profile of sample containing the assembled SCF^{FBXO22}-UNC10088^{C}-NSD2 PWWP1-BACH1 BTB complex (left) used for structural determination by cryo-EM and its representative SYPRO-stained SDS-PAGE gel (right). Source data are provided as a source data file.

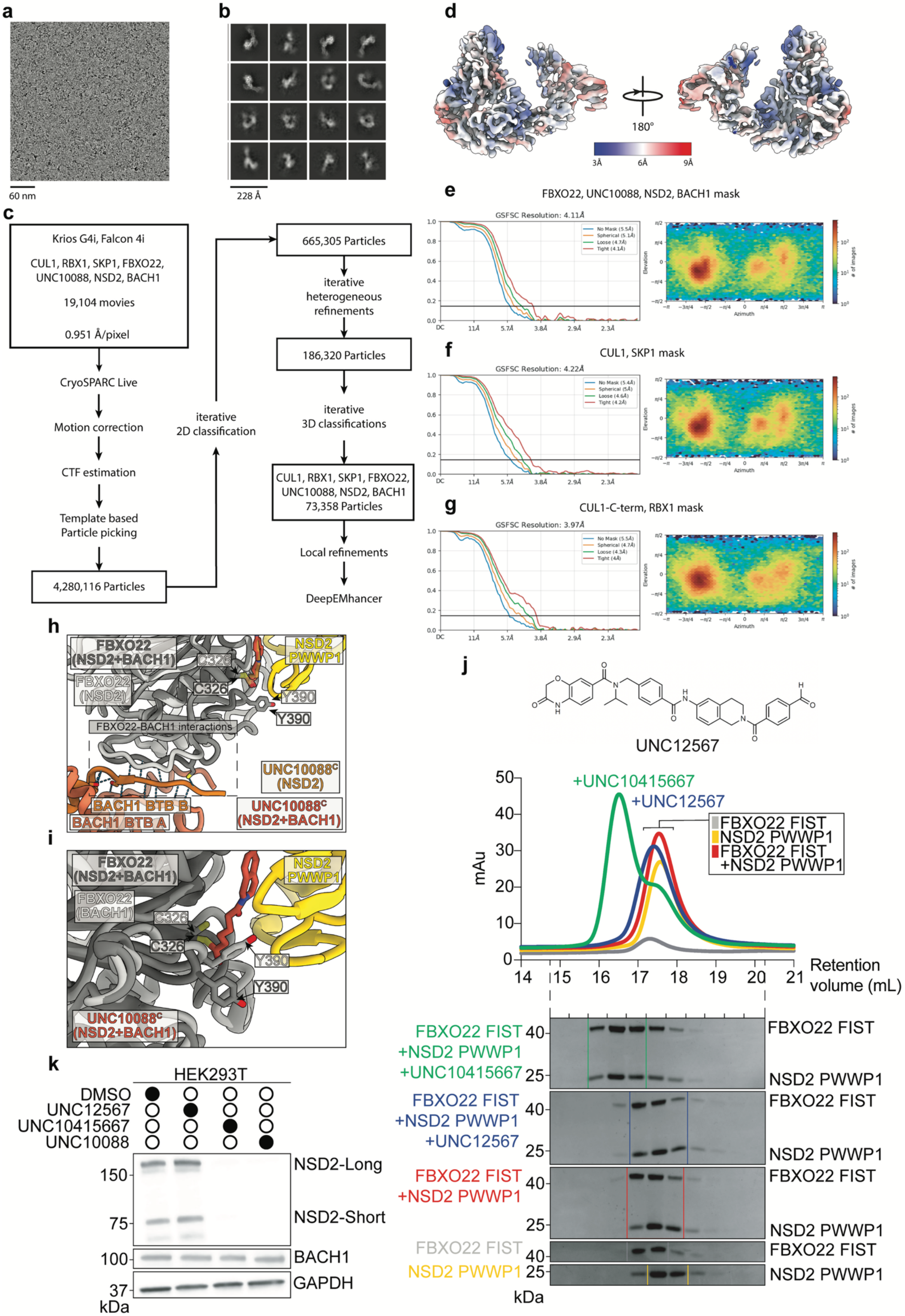

a
60 nm
b
228 Å
c
Krios G4i, Falcon 4i
CUL1, RBX1, SKP1, FBXO22, UNC10088, NSD2, BACH1
19,104 movies
0.951 Å/pixel
CryoSPARC Live
Motion correction
CTF estimation
Template based Particle picking
4,280,116 Particles
iterative 2D classification
665,305 Particles
iterative heterogeneous refinements
186,320 Particles
iterative 3D classifications
CUL1, RBX1, SKP1, FBXO22, UNC10088, NSD2, BACH1 73,358 Particles
Local refinements
DeepEMhancer
d
180°
3Å
6Å
9Å
e
FBXO22, UNC10088, NSD2, BACH1 mask
GSFSC Resolution: 4.11Å
f
CUL1, SKP1 mask
GSFSC Resolution: 4.22Å
g
CUL1-C-term, RBX1 mask
GSFSC Resolution: 3.97Å
h
FBXO22 (NSD2+BACH1)
FBXO22 (NSD2)
NSD2 PWWP1
C326
Y390
FBXO22-BACH1 interactions
UNC10088c (NSD2)
BACH1 BTB B
BACH1 BTB A
UNC10088c (NSD2+BACH1)
i
FBXO22 (NSD2+BACH1)
FBXO22 (BACH1)
NSD2 PWWP1
C326
Y390
UNC10088c (NSD2+BACH1)
j
UNC12567
+UNC10415667
+UNC12567
FBXO22 FIST
NSD2 PWWP1
FBXO22 FIST +NSD2 PWWP1
mAu
Retention volume (mL)
FBXO22 FIST +NSD2 PWWP1 +UNC10415667
FBXO22 FIST +NSD2 PWWP1 +UNC12567
FBXO22 FIST +NSD2 PWWP1
FBXO22 FIST
NSD2 PWWP1
kDa
k
HEK293T
DMSO
UNC12567
UNC10415667
UNC10088
NSD2-Long
NSD2-Short
BACH1
GAPDH
kDa

Supplementary Fig. 8 | Cryo-EM analysis of the SCF^{FBXO22}-UNC10088^{C}-NSD2 PWWP1-BACH1 BTB complex. a) Representative micrograph. **b)** Representative 2D class averages. **c)** Processing pipeline. **d)** Local resolution estimation of local refined map. **e-g)** GSFSC curves of local refined map and viewing angle distributions. **h-i)** Structural comparison of SCF^{FBXO22}-UNC10088^{C}-NSD2 PWWP1-BACH1 BTB with either (h) SCF^{FBXO22}-UNC10088^{C}-NSD2 PWWP1 or (i) SCF^{FBXO22}-BACH1 BTB (PDB: 8UA3)[13]. Close-up views of FBXO22 demonstrate the differences in its configurations of the BACH1 interacting region and Y390-containing loop. **j)** Gel filtration profiles (top) and related Coomassie-stained SDS-PAGE gels (bottom) demonstrate that the negative control compound of UNC10415667 (UNC12567) cannot facilitate NSD2 recruitment (blue) compared to UNC10415667 (green). Individual proteins were subjected to SEC as controls as indicated. n=1 independent experiment. **k)** Immunoblots revealing the degradation of NSD2 when 10 μM of UNC10088 or UNC10415667 are added to HEK293T cells for 6 hours. In contrast, UNC12567 addition did not disrupt the levels of NSD2. BACH1 levels also remained constant upon treatment with compounds. Representative images of n=3 independent experiments. Source data are provided as a source data file.

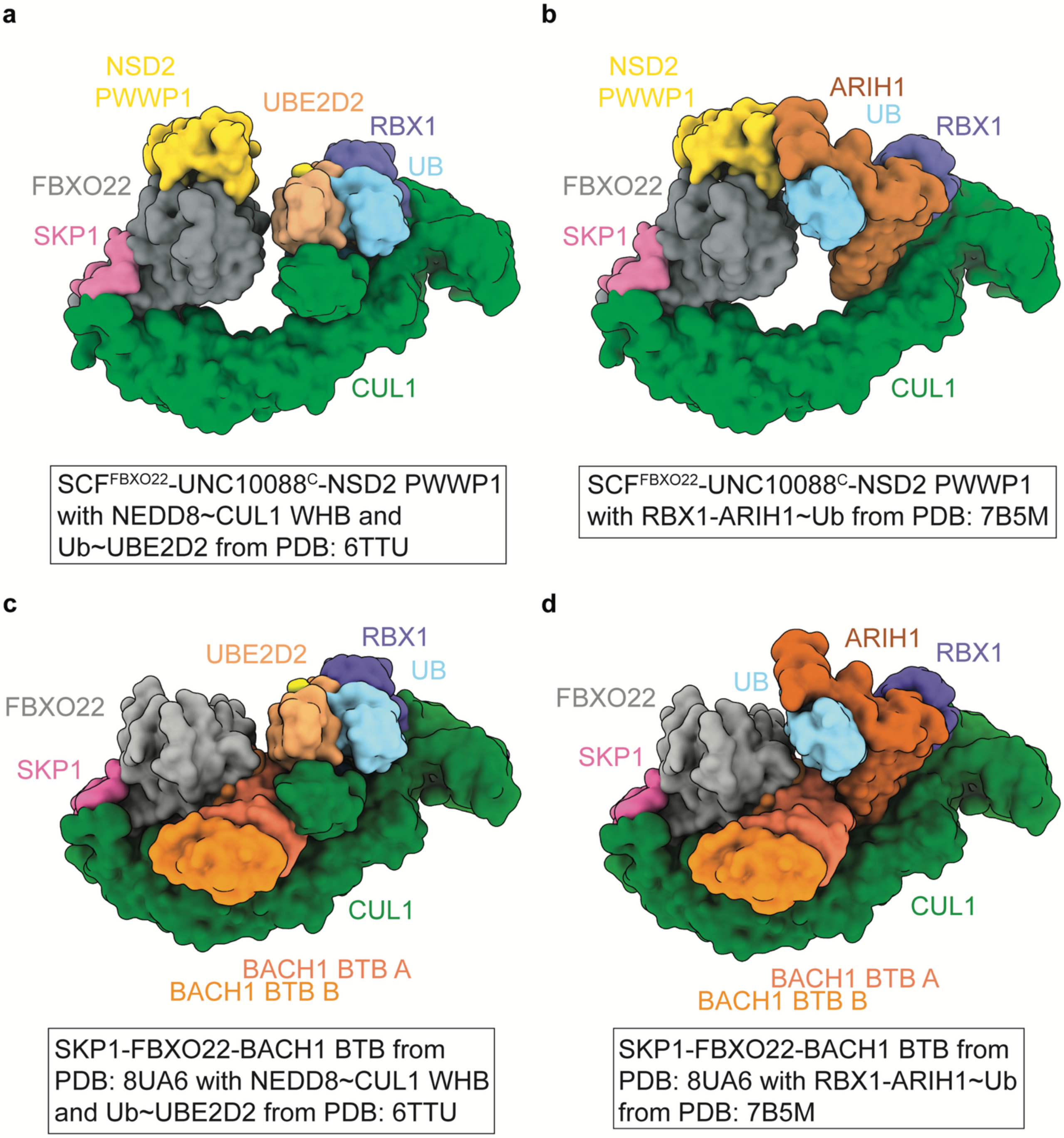

Supplementary Fig. 9 | Structural models of aligning SCF^{FBXO22}-UNC10088-NSD2 (A-B) or SCF^{FBXO22}-BACH1 BTB (C-D) structures with active SCF structures using either UBE2D2 (A,C) or ARIH1 (B,D) as the UCE. a) Overlay of the SCF^{FBXO22}-UNC10088^{C}-NSD2 cryo-EM model surface with NEDD8~CUL1 WHB and Ub~UBE2D2 from an active NEDD8~CUL1-RBX1 N89R-SKP1-monomeric-βTRCPΔD-IκBα-Ub~UBE2D2 complex (PDB: 6TTU)[26]. **b)** Overlay of the SCF^{FBXO22}-UNC10088^{C}-NSD2 cryo-EM model surface with RBX1-ARIH1~Ub from an active CUL1-RBX1-SKP2-CKSHS1-p27~Ub~ARIH1 complex (PDB: 7B5M)[27]. **c)** Overlay of the FBXO22-BACH1 BTB cryo-EM model surface (PDB:8UA6) with NEDD8~CUL1 WHB and Ub~UBE2D2 from an active NEDD8~CUL1-RBX1 N89R-SKP1-monomeric-βTRCPΔD-IκBα-Ub~UBE2D2 complex (PDB: 6TTU)[26]. **d)** Overlay of the FBXO22-BACH1 BTB cryo-EM model surface (PDB:8UA6) with RBX1-ARIH1~Ub from an active CUL1-RBX1-SKP2-CKSHS1-p27~Ub~ARIH1 complex (PDB: 7B5M)[27].

General Chemistry Procedures

Reactions were carried out using conventional glassware. All reagents and solvents were used as received unless otherwise stated. Reagents were of 95% purity or greater, and solvents were reagent grade unless otherwise stated. Any anhydrous solvents used were purchased as “anhydrous” grade and used without further drying. “Room” or ambient temperature varied between 20-25 °C. Analytical thin layer chromatography (TLC) was carried out using glass plates pre-coated with silica gel (Merck) impregnated with fluorescent indicator (254 nm). TLC plates were visualized by illumination with a 254 nm UV lamp. Analytical LCMS data for all compounds were acquired using an Agilent 1260 Infinity II system with the UV detector set to 254 nm. Samples were injected (<25 µL) onto an Agilent ZORBAX Eclipse Plus C18, 600 Bar, 4.6 x 50 mm, 1.8 µM column at 25 °C. Mobile phases A (H_2O + 0.1% acetic acid) and B (MeCN + 0.1% acetic acid) were used with a linear gradient from 10% to 100% B in 5 min, followed by a flush at 100% B for another 2 minutes at a flow rate of 1 mL/min. Mass spectra (MS) data were acquired in positive ion mode using an Agilent 6110 single quadrupole mass spectrometer with an electrospray ionization (ESI) source. Normal phase column chromatography was performed with a Teledyne Isco CombiFlash®Rf 200 using RediSep®Rf SILICA columns with the UV detector set to 254 nm and 280 nm. Reverse phase column chromatography was performed with a Teledyne Isco CombiFlash®Rf 200 using C18 RediSep®Rf Gold columns with the UV detector set to 220 nm and 254 nm. Mobile phases A (H_2O + 0.1% TFA) and B (MeOH or MeCN) were used. Preparative HPLC was performed as follows unless otherwise noted: Preparative HPLC was performed using an Agilent Prep 1200 series with the UV detector set to 220 nm and 254 nm. Samples were injected onto either a Phenomenex Luna 250 x 30 mm (5 µm) C18 column, a Phenomenex Luna 250 x 50mm (10 µm) C18 column, a Phenomenex Luna 80 x 40mm (3 µm) C18 column, or a Phenomenex Luna 75 x 30 mm (5 µm) C18 column at rt. Mobile phases A (H_2O + 0.1% TFA or H_2O + 0.1% formic acid) and B (MeOH or MeCN) were used with a flow rate of 20 mL/min for the larger column and 15 mL/min for the smaller column. Analytical LCMS (at 254 nm) was used to establish the purity of targeted compounds. All compounds that were evaluated in biochemical and biophysical assays had >95% purity as determined by LCMS or NMR (spectra provided in NMR Spectra Section).

Nuclear Magnetic Resonance Spectroscopy (NMR)

^{1}H and ^{13}C NMR spectra were obtained on a Bruker AVANCE NEO 400MHz Nanobay spectrometer equipped with a 5mm iProbe or on a Bruker AVANCE NEO 500 MHz spectrometer equipped with a 5mm Prodigy CryoProbe. Chemical shifts are reported in ppm (δ) relative to

residual protons in deuterated solvent peaks, and coupling constants are reported in Hz. CDCl3 referenced at 7.26 (^{1}H), DMSO-d6 referenced at 2.50 (^{1}H), acetone-d6 referenced at 2.05 (^{1}H), and MeOH-d4 referenced at 3.31 (^{1}H). All compounds that were evaluated in biochemical, biophysical and cellular assays had >95% purity as determined by ^{1}H NMR and LCMS (spectra provided in NMR Spectra Section).

Abbreviations Used

ACN/MeCN Acetonitrile

$Bi(OTf)_3$ Bismuth(III) trifluoromethanesulfonate

DCM Dichloromethane

DIPEA N,N-Diisopropylethylamine

DMAP 4-Dimethylaminopyridine

DMF N,N-Dimethylformamide

DMSO Dimethylsulfoxide

EDC 1-Ethyl-3-(3-dimethylaminopropyl)carbodiimide

ESI Electrospray Ionization

EtOAc Ethyl acetate

EtOH Ethanol

HATU Hexafluorophosphate Azabenzotriazole Tetramethyl Uronium

HCl Hydrochloric acid

HOAt 1-Hydroxy-7-azabenzotriazole

HPLC High-Perfomance Liquid Chromatography

IPA Isopropanol

K_3PO_4 Tripotassium phosphate

LCMS Liquid Chromatography-Mass Spectrometry

$LiOH{\cdot}H_2O$ Lithium hydroxide monohydrate

MeOH Methanol

MeOD Deuterated methanol

$NaBH_4$ Sodium borohydride

$NaHCO_3$ Sodium bicarbonate

NMR Nuclear Magnetic Resonance

NMI 1-Methylimidazole

Pd/C Palladium on carbon

Pd(dppf)Cl_2·DCM [1,1′-Bis(diphenylphosphino)ferrocene]dichloropalladium(II), complex with dichloromethane

PTSA p-Toluenesulfonic acid

PTSA·H_2O p-Toluenesulfonic acid monohydrate

TBME Methyl tert-butyl ether

TCFH N,N,N',N'-tetramethylchloroformamidinium hexafluorophosphate

TFA Trifluoroacetic acid

THF Tetrahydrofuran

TLC Thin Layer Chromatography

9-BBN 9-borabicyclo[3.3.1]nonane

Synthetic Procedures

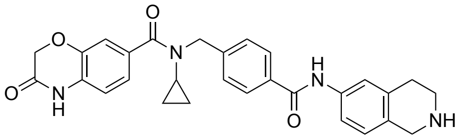

1

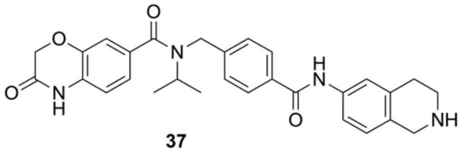

37

Intermediate **1** and **37** were prepared according to previously published procedures[9] and their respective spectroscopic signatures (^{1}H NMR and LCMS) were found to be consistent with values reported therein.

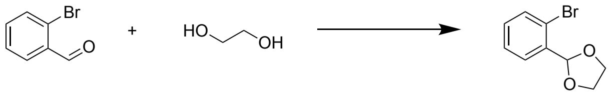

2

2-(2-Bromophenyl)-1,3-dioxolane (2)

To a multi-neck round bottom flask connected with Dean Stark apparatus was added 2-bromobenzaldehyde (1.00 eq, 3.10 mL, 27.0 mmol), anhydrous toluene (50.0 mL) followed by ethylene glycol (1.20 eq, 1.80 mL, 32.4 mmol) and PTSA·H_2O (0.006 eq, 31.0 mg, 0.162 mmol) at room temperature under inert atmosphere. The reaction was allowed to stir for 16 hours at 125 °C. After completion of the reaction monitored by TLC, the reaction mixture was cooled to room temperature, quenched with sodium bicarbonate solution (100 mL), and extracted with DCM (3 × 30 mL). Combined organic layers were dried over sodium sulfate, filtered, and evaporated under reduced pressure. The crude product was purified by normal phase flash column chromatography (5-10% ethyl acetate in petroleum ether) to afford **2-(2-bromophenyl)-1,3-dioxolane (2)** (2.94 g, 12.1 mmol, yield 45%) as a colorless liquid. The product was used without further purification.

^{1}H-NMR (400 MHz, $CDCl_3$): δ 7.63 (dd, *J* = 7.80, 2.00 Hz, 1H), 7.59 (dd, *J* = 7.80, 1.20 Hz, 1H), 7.36 (td, *J* = 7.40, 1.20 Hz, 1H), 7.25 (td, *J* = 7.60, 2.00 Hz, 1H), 4.21-4.08 (m, 4H).
LCMS (ESI, +ve mode): Expected *m/z* for $[C_9H_{10}BrO_2]^+$ [M+H] 230.1, found 230.9.

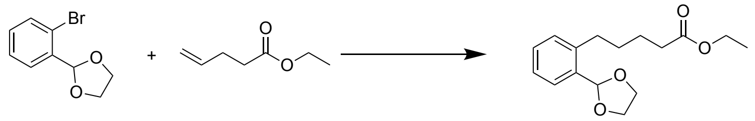

2 3

Ethyl 5-(2-(1,3-dioxolan-2-yl)phenyl)pentanoate (3)

To a stirred solution of ethyl pent-4-enoate (1.10 eq, 615 mg, 4.80 mmol) in anhydrous THF (4.0 mL) in a sealed tube maintained under inert atmosphere was slowly added 9-BBN (0.5 M in THF) (1.50 eq, 13.0 mL, 6.55 mmol) at 0 °C. The Reaction was allowed to slowly reach the room temperature and stirred for 4 hours. To the above solution was added anhydrous DMF (35.0 mL), Pd(dppf)Cl_2·DCM (0.03 eq, 107 mg, 0.131 mmol), 2-(2-bromophenyl)-1,3-dioxolane (2) (1.00 eq, 0.67 mL, 4.37 mmol) followed by potassium carbonate (2.00 eq, 1.205 g, 8.73 mmol) at room temperature under inert atmosphere. The reaction mixture was heated to 50 °C and stirred for 16 hours. After completion of the reaction monitored by TLC and LCMS, the reaction mixture was poured into water and extracted with ethyl acetate (3 × 30 mL). Combined organic layers were washed with brine (50 mL), dried over sodium sulfate, filtered, and evaporated under reduced pressure. The crude product was purified by normal phase flash column chromatography (5-10% ethyl acetate in petroleum ether) to afford **ethyl 5-[2-(1,3-dioxolan-2-yl)phenyl]pentanoate (3)** (917 mg, 3.10 mmol, yield 71%) as a clear resin.
^{1}HNMR (400 MHz, $CDCl_3$): δ 7.57 (dd, *J* = 1.60, 7.60 Hz, 1H), 7.31-7.28 (m, 1H), 7.26-7.19 (m, 2H), 6.00 (s, 1H), 4.18-4.12 (m, 4H), 4.08-4.06 (m, 2H), 2.77 (t, *J* = 7.20 Hz, 2H), 2.36 (t, *J* = 7.20 Hz, 2H), 1.54-1.48 (m, 4H), 1.27 (t, *J* = 7.20 Hz, 3H).
LCMS (ESI, +ve mode): Expected *m/z* for $[C_{16}H_{23}O_4]^+$ [M+H] 279.3, found 279.0.

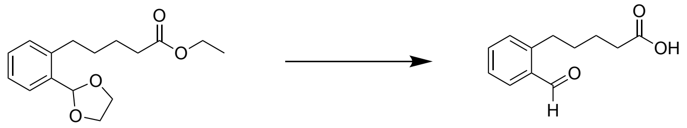

3 4

5-(2-Formylphenyl)pentanoic acid (4)

To a stirred solution of ethyl 5-[2-(1,3-dioxolan-2-yl)phenyl]pentanoate (3) (1.00 eq, 319 mg, 1.08 mmol) in THF:EtOH:water (2:1:1, 4.0 mL) was added lithium hydroxide monohydrate (3.00 eq, 136 mg, 3.23 mmol) at room temperature. The reaction was stirred at room temperature for 2 hours. After completion of the reaction monitored by TLC, solvent was removed partially under

reduced pressure. The residual aqueous layer was washed with TBME (2 × 20 mL) to remove nonpolar impurities. The aqueous layer was acidified with 1.5 N HCl at 0-5 °C and extracted with ethyl acetate (3 × 20 mL). Combined organic layers were dried over sodium sulfate, filtered, and concentrated under reduced pressure to afford **5-(2-formylphenyl)pentanoic acid (4)** (156 mg, 0.673 mmol, yield 62%) as a pale brown solid.

^{1}H-NMR (400 MHz, DMSO-d_6): δ 10.26 (s, 1H), 7.84 (dd, J = 1.20, 7.60 Hz, 1H), 7.55-7.50 (m, 1H), 7.42-7.38 (m, 1H), 7.29 (d, J = 7.60 Hz, 1H), 3.07 (t, J = 6.80 Hz, 2H), 2.44-2.39 (m, 2H), 1.91-1.64 (m, 4H).

LCMS (ESI, -ve mode): Expected m/z for $[C_{12}H_{13}O_3]^-$ [M-H] 205.2, found 205.2.

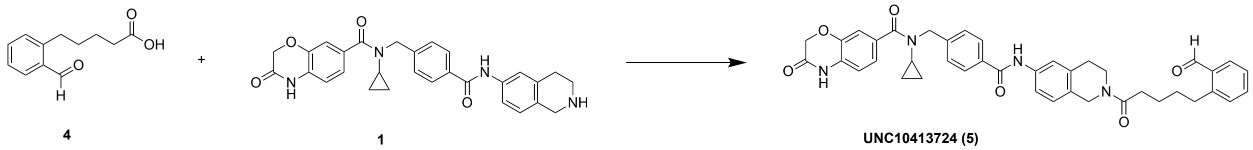

4 1 UNC10413724 (5)

N-Cyclopropyl-N-(4-((2-(5-(2-formylphenyl)pentanoyl)-1,2,3,4-tetrahydroisoquinolin-6-yl)carbamoyl)benzyl)-3-oxo-3,4-dihydro-2H-benzo[b][1,4]oxazine-7-carboxamide (UNC10413724, or 5)

To a stirred solution of *N*-cyclopropyl-3-oxo-*N*-[[4-(1,2,3,4-tetrahydroisoquinolin-6-ylcarbamoyl)phenyl]methyl]-4H-1,4-benzoxazine-7-carboxamide;hydrochloride (1) (1.10 eq, 158 mg, 0.267 mmol) in anhydrous DMF (2.0 mL) in a reaction flask was added NMI (3.00 eq, 60.0 mg, 0.727 mmol) at room temperature under inert atmosphere. To the above solution was added 5-(2-formylphenyl)pentanoic acid (4) (1.00 eq, 50.0 mg, 0.242 mmol) followed by TCFH (1.10 eq, 75.0 mg, 0.267 mmol) at room temperature under nitrogen atmosphere. The reaction mixture was stirred at the room temperature for 1 hour. After completion of the reaction monitored by LCMS, the reaction was concentrated under reduced pressure and the crude product was purified by prep-HPLC (5-100% ACN in H_2O + 0.1% formic acid) to afford ***N*-cyclopropyl-*N*-[[4-[[2-[5-(2-formylphenyl)pentanoyl]-3,4-dihydro-1H-isoquinolin-6-yl]carbamoyl]phenyl]methyl]-3-oxo-4H-1,4-benzoxazine-7-carboxamide (UNC10413724, or 5)** (75.0 mg, 0.109 mmol, yield 45%) as an off-white solid.

^{1}H-NMR (400 MHz, DMSO-d_6): δ 10.88 (s, 1H), 10.27 (s, 1H), 10.18 (s, 1H), 7.94 (d, J = 8.40 Hz, 2H), 7.83 (d, J = 7.60 Hz, 1H), 7.66-7.53 (m, 3H), 7.46-7.35 (m, 4H), 7.20-7.15 (m, 3H), 6.93 (d, J = 8.00 Hz, 1H), 4.72 (s, 2H), 4.62-4.56 (m, 4H), 3.67 (t, J = 6.00 Hz, 2H), 3.05-3.04 (m, 2H), 2.86-2.73 (m, 3H), 2.51-2.44 (m, 2H), 1.60-1.59 (m, 4H), 0.56-0.49 (m, 4H).

LCMS (ESI, +ve mode): Expected m/z for $[C_{41}H_{41}N_4O_6]^+$ [M+H] 685.8, found 685.1.

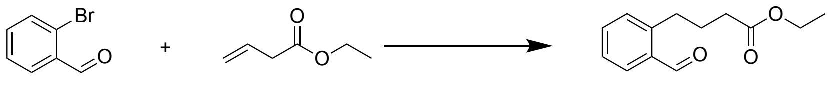

6

4-(2-formylphenyl)butanoate (6)

To a solution of ethyl but-3-enoate (1.10 eq, 407 mg, 3.57 mmol) in anhydrous THF (2.0 mL) at 0 °C was added 9-BBN solution (0.5 M in THF) (1.51 eq, 9.80 mL, 4.90 mmol). The reaction mixture was allowed to reach the room temperature and stirred for 4 hours. Then, the reaction was diluted with anhydrous DMF (35.0 mL), followed by the addition of Pd(dppf)Cl_2·CH_2Cl_2 (0.0300 eq, 79.0 mg, 0.0973 mmol), 2-bromobenzaldehyde (1.00 eq, 600 mg, 3.24 mmol), and potassium carbonate (2.00 eq, 895 mg, 6.49 mmol). The reaction was heated at 50 °C for 16 hours. After completion of the reaction monitored by LCMS, the reaction mixture was quenched with water (150 mL) and extracted with EtOAc (3 × 30 mL). The combined organic layers were washed with water and brine, dried over sodium sulfate, filtered, and concentrated *in vacuo*. The crude product was purified by normal phase flash column chromatography (0-10% EtOAc in petroleum ether) to afford **ethyl 4-(2-formylphenyl)butanoate (6)** (600 mg, 2.46 mmol, 76% yield) as a clear oil. The product was used without further purification.

^{1}H-NMR (400 MHz, DMSO-*d*$_6$): δ 10.26 (s, 1H), 7.85 (dd, *J* = 7.60, 1.60 Hz, 1H), 7.63-7.59 (m, 1H), 7.47-7.45 (m, 1H), 7.36 (d, *J* = 7.60 Hz, 1H), 4.05 (q, *J* = 7.20 Hz, 2H), 3.05-3.01 (m, 2H), 2.35 (t, *J* = 7.20 Hz, 2H), 1.82-1.75 (m, 2H), 1.18 (t, *J* = 7.20 Hz, 3H).

LCMS (ESI, +ve mode): Expected *m/z* for $[C_{13}H_{17}O_3]^+$ [M+H] 221.2, found: 221.1.

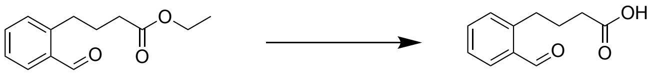

6 7

4-(2-Formylphenyl)butanoic acid (7)

To a stirred solution of ethyl 4-(2-formylphenyl)butanoate (6) (1.00 eq, 100 mg, 0.409 mmol) in 1,4-dioxane (2.0 mL) was added 20% aq. sulfuric acid (5.0 mL) at room temperature. The reaction mixture was stirred at 100 °C for 4 hours. After completion of the reaction monitored by LCMS, the solvent was evaporated, diluted with water (10 mL), and extracted with EtOAc (3 × 20 mL). The combined organic layers were washed with water once, dried over sodium sulfate, filtered, and concentrated *in vacuo* to afford **4-(2-formylphenyl)butanoic acid (7)** (65 mg, 0.237 mmol, 58% yield) as a pale-yellow resin.

LCMS (ESI, -ve mode): Expected *m/z* for $[C_{11}H_{11}O_3]^-$ [M-H] 191.2, found 191.2. The product was used without further purification.

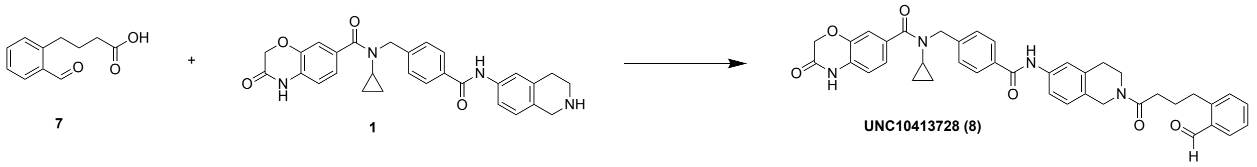

N-Cyclopropyl-N-(4-((2-(4-(2-formylphenyl)butanoyl)-1,2,3,4-tetrahydroisoquinolin-6-yl)carbamoyl)benzyl)-3-oxo-3,4-dihydro-2H-benzo[b][1,4]oxazine-7-carboxamide (UNC10413728, or 8)

To a stirred solution of 4-(2-formylphenyl)butanoic acid (7) (1.00 eq, 60 mg, 0.241 mmol) and *N*-cyclopropyl-3-oxo-*N*-[[4-(1,2,3,4-tetrahydroisoquinolin-6-ylcarbamoyl)phenyl]methyl]-4H-1,4-benzoxazine-7-carboxamide (1) (1.00 eq, 125 mg, 0.241 mmol) in anhydrous DMF (3.0 mL) was added DIPEA (5.00 eq, 0.21 mL, 1.20 mmol) and HATU (1.20 eq, 110 mg, 0.289 mmol) at 25 °C under N_2 atmosphere. The resulting reaction mixture was stirred at room temperature for 16 hours. After completion of the reaction monitored by LCMS, the reaction mixture was concentrated *in vacuo* and was purified by prep-HPLC (5-100% ACN in H_2O + 0.1% formic acid) to afford ***N*-cyclopropyl-*N*-[[4-[[2-[4-(2-formylphenyl)butanoyl]-3,4-dihydro-1H-isoquinolin-6-yl]carbamoyl]phenyl]methyl]-3-oxo-4H-1,4-benzoxazine-7-carboxamide (UNC10413728, or 8)** (10.0 mg, 0.0144 mmol, 6% yield) as an off-white solid.

^{1}H-NMR (400 MHz, DMSO-d_6): δ 10.88 (br s, 1H), 0.32 (s, 1H), 10.18 (s, 1H), 7.94 (d, *J* = 8.40 Hz, 2H), 7.84 (d, *J* = 7.60 Hz, 1H), 7.67-7.53 (m, 3H), 7.46-7.35 (m, 4H), 7.20-7.15 (m, 3H), 6.93 (d, *J* = 8.00 Hz, 1H), 4.72 (s, 2H), 4.62-4.58 (m, 4H), 3.70-3.65 (m, 2H), 2.87-2.84 (m, 2H), 2.80-2.73 (m, 3H), 2.53-2.50 (m, 2H), 1.84-1.78 (m, 2H), 0.56-0.49 (m, 4H).

LCMS (ESI, +ve mode): Expected *m/z* for $[C_{40}H_{39}N_4O_6]^+$ [M+H] 671.7, found: 671.0.

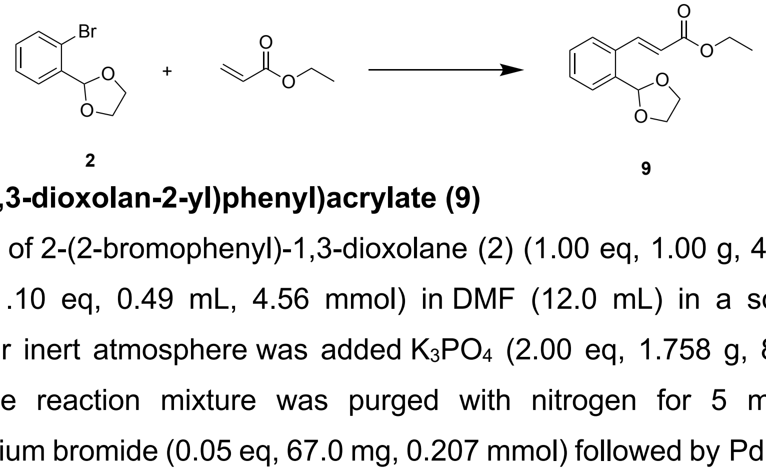

Ethyl (E)-3-(2-(1,3-dioxolan-2-yl)phenyl)acrylate (9)

To a suspension of 2-(2-bromophenyl)-1,3-dioxolane (2) (1.00 eq, 1.00 g, 4.15 mmol) and ethyl prop-2-enoate (1.10 eq, 0.49 mL, 4.56 mmol) in DMF (12.0 mL) in a screw cap glass vial maintained under inert atmosphere was added K_3PO_4 (2.00 eq, 1.758 g, 8.29 mmol) at room temperature. The reaction mixture was purged with nitrogen for 5 minutes, and added tetrabutylammonium bromide (0.05 eq, 67.0 mg, 0.207 mmol) followed by Pd(dppf)Cl_2·DCM (0.05 eq, 169 mg, 0.207 mmol). The reaction mixture was stirred at 120 °C for 4 hours and then allowed

to stand at room temperature for 12 hours. After completion of the reaction monitored by TLC, the reaction was diluted with water and extracted with EtOAc (2 × 60 mL). Combined organic extracts were washed with water once, brine once, dried over sodium sulfate, filtered, and concentrated under reduced pressure. The crude product was purified by normal phase flash column chromatography (10-13% EtOAc in petroleum) to afford **ethyl (*E*)-3-[2-(1,3-dioxolan-2-yl)phenyl]prop-2-enoate (9)** (900 mg, 3.44 mmol, yield 83%) as a pale-yellow liquid.

^{1}H-NMR (400 MHz, DMSO-d_6): δ 8.06 (d, J = 16.00 Hz, 1H), 7.84-7.82 (m, 1H), 7.58-7.56 (m, 1H), 7.47-7.44 (m, 2H), 6.54 (d, J = 16.00 Hz, 1H), 5.99 (s, 1H), 4.20 (q, J = 7.20 Hz, 2H), 4.11-4.00 (m, 4H), 1.27 (t, J = 6.80 Hz, 3H).

LCMS (ESI, +ve mode): Expected *m/z* for $[C_{14}H_{17}O_4]^+$ [M+H] 249.3, found 249.1.

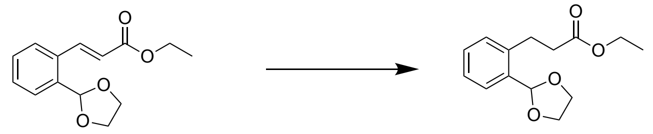

9 10

Ethyl 3-(2-(1,3-dioxolan-2-yl)phenyl)propanoate (10)

To a stirred solution of ethyl (*E*)-3-[2-(1,3-dioxolan-2-yl)phenyl]prop-2-enoate (9) (1.00 eq, 300 mg, 1.15 mmol) in MeOH (4.0 mL) was added cobalt(II) chloride hexahydrate (0.30 eq, 82.0 mg, 0.344 mmol) at 0 °C under N_2 atmosphere. The reaction mixture was stirred at room temperature for 30 minutes. Next, a solution of $NaBH_4$ (5.00 eq, 217 mg, 5.74 mmol) in DMF (3.0 mL) was added dropwise at 0 °C. The reaction was stirred at room temperature for 0.5 hours. After completion of the reaction monitored by TLC, MeOH was removed *in vacuo*. The residual DMF layer was diluted with water and extracted with EtOAc (2 × 50 mL). Combined organic layers were washed with water once, brine once, dried over sodium sulfate, filtered, and concentrated under reduced pressure. The crude product was purified by normal phase column chromatography (8% EtOAc in petroleum ether) to afford **ethyl 3-[2-(1,3-dioxolan-2-yl)phenyl]propanoate (10)** (280 mg, 1.05 mmol, yield 92%) as a colorless liquid.

^{1}H-NMR (400 MHz, DMSO-d_6): δ 7.48-7.46 (m, 1H), 7.33-7.29 (m, 1H), 7.25-7.21 (m, 2H), 5.92 (s, 1H), 4.09-3.95 (m, 6H), 2.96 (t, J = 8.00 Hz, 2H), 2.59 (t, J = 7.60 Hz, 2H), 1.17 (t, J = 6.80 Hz, 3H).

LCMS (ESI, +ve mode): Expected *m/z* for $[C_{14}H_{19}O_4]^+$ [M+H] 251.3 found 251.1.

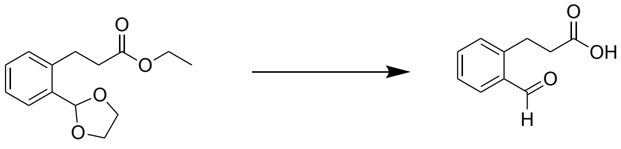

10 11

3-(2-Formylphenyl)propanoic acid (11)

To a stirred solution of ethyl 3-[2-(1,3-dioxolan-2-yl)phenyl]propanoate (10) (1.00 eq, 275 mg, 1.03 mmol) in THF:EtOH:water (2:1:1, 4.0 mL) was added lithium hydroxide monohydrate (4.00 eq, 173 mg, 4.13 mmol) at 0 °C. The reaction mixture was stirred at room temperature for 2 hours. After completion of the reaction monitored by TLC, solvent was removed partially under reduced pressure at 40 °C. The Residual aqueous layer was washed with hexane (10 mL) to remove non polar impurities. The aqueous layer was acidified with 1.5 N HCl at 0 °C and extracted with EtOAc (2 × 25 mL). Combined organic layer was washed with water once, dried over sodium sulfate, filtered, and concentrated *in vacuo* to afford **3-(2-formylphenyl)propanoic acid (11)** (140 mg, 0.739 mmol, yield 72%) as an off-white solid, which was used without further purification.

^{1}H-NMR (400 MHz, DMSO-d_6): δ 12.19 (br s, 1H), 10.23 (s, 1H), 7.86 (dd, *J* = 1.20, 7.60 Hz, 1H), 7.62-7.58 (m, 1H), 7.48-7.40 (m, 2H), 3.24 (t, *J* = 7.60 Hz, 2H), 2.56-2.51 (m, 2H).

LCMS (ESI, -ve mode): Expected *m/z* for $[C_{10}H_9O_3]^-$ [M-H] 177.2, found 177.1.

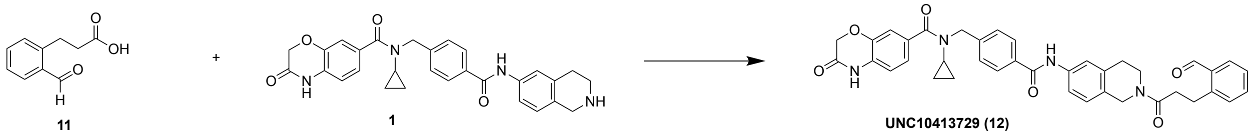

11 1 UNC10413729 (12)

N-Cyclopropyl-N-(4-((2-(3-(2-formylphenyl)propanoyl)-1,2,3,4-tetrahydroisoquinolin-6-yl)carbamoyl)benzyl)-3-oxo-3,4-dihydro-2H-benzo[b][1,4]oxazine-7-carboxamides (UNC10413729, or 12)

To a stirred solution of *N*-cyclopropyl-3-oxo-*N*-[[4-(1,2,3,4-tetrahydroisoquinolin-6-ylcarbamoyl)phenyl]methyl]-4H-1,4-benzoxazine-7-carboxamide (1) (1.00 eq, 146 mg, 0.264 mmol) in DMF (2.0 mL) was added NMI (6.00 eq, 0.13 mL, 1.58 mmol) followed by 3-(2-formylphenyl)propanoic acid (11) (1.00 eq, 50.0 mg, 0.264 mmol) and TCFH (1.10 eq, 81.0 mg, 0.290 mmol) at 0 °C. The reaction was stirred under N_2 atmosphere at room temperature for 1 hour. After completion of the reaction monitored by LCMC, the reaction was concentrated *in vacuo* and purified by prep-HPLC (5-100% ACN in H_2O + 0.1% formic acid) to afford ***N*-cyclopropyl-*N*-[[4-[[2-[3-(2-formylphenyl)propanoyl]-3,4-dihydro-1H-isoquinolin-6-**

yl]carbamoyl]phenyl]methyl]-3-oxo-4H-1,4-benzoxazine-7-carboxamide (UNC10413729, or 12) (5.0 mg, 0.00760 mmol, yield 3%) as an off-white solid.

^{1}H-NMR (400 MHz, DMSO-d_6): δ 10.89 (s, 1H), 10.27 (s, 1H), 10.18 (s, 1H), 7.94 (d, J = 7.60 Hz, 2H), 7.85 (t, J = 7.20 Hz, 1H), 7.64-7.56 (m, 3H), 7.45-7.44 (m, 4H), 7.20-7.10 (m, 3H), 6.94-6.92 (m, 1H), 4.72-4.58 (m, 6H), 3.67-3.62 (m, 2H), 3.33-3.27 (m, 2H), 2.80-2.68 (m, 5H), 0.56-0.48 (m, 4H).

LCMS (ESI, +ve mode): Expected *m/z* for $[C_{39}H_{37}N_4O_6]^+$ [M+H] 657.7, found 657.0.

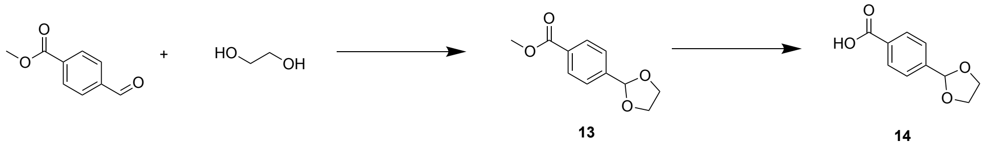

13 14

4-(1,3-dioxolan-2-yl)benzoic acid (14)

To a scintillation vial was added **methyl 4-formylbenzoate** (100.0 mg, 1.0 Eq, 609.2 µmol), ethane-1,2-diol (189.1 mg, 170.0 µL, 5.0 Eq, 3.046 mmol), PTSA (11.7 mg, 90% Wt, 0.1 Eq, 60.92 µmol), and Toluene (3.0 mL). The reaction was allowed to stir for 16 hours at room temperature. The reaction mixture was concentrated with a steady nitrogen blow. The resulting crude product was purified by normal phase flash chromatography (0-100% EtOAc in Hexane). The pure product was collected and concentrated *in vacuo* to give methyl 4-(1,3-dioxolan-2-yl)benzoate (13) (94.3 mg, 453 µmol, 74.3 %) as a clear oil, which was used right away. To a scintillation vial charged with a stir bar was added 4-(1,3-dioxolan-2-yl)benzoate (13) (94.3 mg, 453 µmol, 74.3 %) in THF (1.0 mL). LiOH·H_2O (54.2 mg, 5 Eq, 2.26 mmol) was dissolved in H_2O (1.0 mL) and the solution was added drop-wise into the scintillation vial. The reaction was stirred at room temperature for 16 hours. The reaction mixture was quenched with 1M HCl (pH was adjusted to 6) and was extracted with DCM (3 x 30 mL). The organic layers were combined and washed with brine once (50 mL), dried over sodium sulfate, filtered, and concentrated *in vacuo* to provide **4-(1,3-dioxolan-2-yl)benzoic acid (14)** (76.7 mg, 395 µmol, 64.8 % over 2 steps) as an off-white solid.

^{1}H NMR (400 MHz, DMSO-d$_6$) δ 13.05 (s, 1H),7.95 (d, J = 8.4 Hz, 2H), 7.54 (d, J = 8.4 Hz, 2H), 5.80 (s, 1H),4.07-3.94 (m, 4H)

LCMS (ESI, +ve mode): Expected *m/z* for $[C_{10}H_{11}O_4]^+$ [M+H] 195.1, found 195.2.

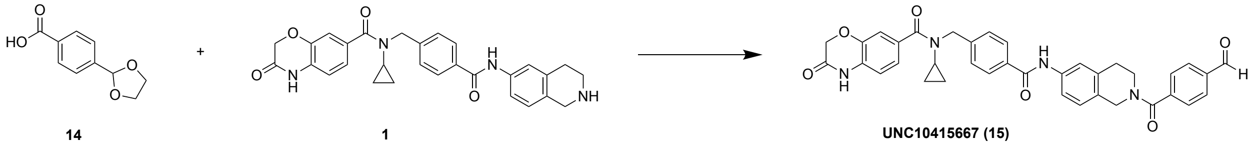

14 1 UNC10415667 (15)

N-cyclopropyl-N-(4-((2-(4-formylbenzoyl)-1,2,3,4-tetrahydroisoquinolin-6-yl) carbamoyl) benzyl)-3-oxo-3,4-dihydro-2H-benzo[b] [1,4] oxazine-7-carboxamide (UNC10415667, or 15)

To a scintillation vial charged with a stir bar was added 4-(1,3-dioxolan-2-yl) benzoic acid (14) (50.0 mg, 250 μmol, 1.0 Eq), DMF (0.5 mL), HATU (140 mg, 380 μmol, 1.5 Eq), and DIPEA (100 mg, 770 μmol, 3.0 Eq). The mixture was left to stir at room temperature for 10 minutes. To the vial was then added N-cyclopropyl-3-oxo-N-(4-((1,2,3,4-tetrahydroisoquinolin-6-yl) carbamoyl) benzyl)-3,4-dihydro-2H-benzo[b] [1,4] oxazine-7-carboxamide (1) (127.0 mg, 250 μmol, 1.0 Eq). The reaction was stirred at room temperature for 3 hours. After completion of the reaction monitored by TLC and LCMS, the reaction mixture was poured in to ice cold water. The separated solid was filtered and dried to get the pure intermediate as an off-white solid, which was used right away. To a scintillation vial charged with a stir bar was added the intermediate (50.0 g, 70 μmol, 1.0 Eq) followed by formic acid (0.5 mL). The reaction was allowed to stir at room temperature for 8 hours. After completion of the reaction, the reaction mixture was poured in to water, and the product was extracted with DCM (3 x 50 mL). The organic layers were combined and washed with $NaHCO_3$ once (30 mL), dried over sodium sulfate, filtered, and concentrated *in vacuo* to provide the crude compound. The crude compound was purified by prep-HPLC (5-100% ACN in H_2O + 0.1% formic acid). The pure fractions were collected, suspended in water, flash frozen and lyophilized to dryness to give **N-cyclopropyl-N-(4-((2-(4-formylbenzoyl)-1,2,3,4-tetrahydroisoquinolin-6-yl) carbamoyl) benzyl)-3-oxo-3,4-dihydro-2H-benzo[b] [1,4] oxazine-7-carboxamide (UNC10415667, or 15)** (17 mg, 27.06 μmol, 10.7% over 2 steps) as a white solid.

^{1}H NMR (400 MHz, DMSO-d6) δ 10.63 (s, 1H), 10.09 (s, 1H), 9.98 (s, 1H), 7.99 (d, J=7.6 Hz, 2H), 7.94 (d, J=8.0 Hz, 2H), 7.65 (d, J= 7.6 Hz, 3H), 7.57 (d, J= 7.6 Hz, 1H),7.43 (d, J=8.0 Hz, 2H), 7.15 (d, J= 8.0 Hz, 2H), 7.10 (s, 1H), 6.94 (d, J= 8 Hz, 1H), 4.71 (s, 2H), 4.65 (s, 2H), 4.59 (s, 2H), 3.68 (s, 2H), 2.79 (s 2H), 2.78 (t, J= 4.0 Hz, 1H), 1.27 (d, J= 12.8 Hz, 2H), 0.57 (d, J= 6.8 Hz, 2H)
LCMS (ESI, +ve mode): Expected *m/z* for $[C_{37}H_{33}N_4O_6]^+$ [M+H] 629.2, found 629.2

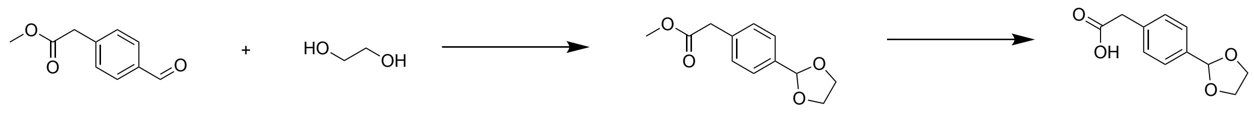

16 17 18

2-(4-(1,3-dioxolan-2-yl) phenyl) acetic acid (18)

To a scintillation vial was added methyl 2-(4-formylphenyl) acetate (16) (0.30 g, 1.68 mmol, 1.0 Eq), ethane-1,2-diol (0.20 g, 3.36 mmol, 2.0 Eq), $Bi(OTf)_3$ (0.022 g, 0.033 mmol, 0.02 Eq), and Toluene (4.5 mL). The reaction was allowed to stir for 3 hours at 100°C. After completion of the

reaction, the reaction mixture was poured into water and was extracted with EtOAc (3 x 50 mL). The organic layers were combined and washed with brine once (50 mL), dried over sodium sulfate, filtered, and concentrated *in vacuo* to provide the crude compound. The resulting crude compound was purified by normal phase flash column chromatography (0-100% EtOAc in Hexane). The pure product was collected and concentrated *in vacuo* to give methyl 2-(4-(1,3-dioxolan-2-yl) phenyl) acetate (0.10 g, 0.45 mmol, 27%) (17) as a clear oil, which was used right away. To a stirred solution of methyl 2-(4-(1,3-dioxolan-2-yl) phenyl) acetate (0.10 g, 0.45 mmol, 1.0 Eq) (17) in THF (1 mL), was added lithium hydroxide (0.038 g, 0.90 mmol, 2.0 Eq) in H_2O (1 mL). Reaction mixture was stirred at room temperature for 2 hours. After completion of the reaction, the reaction mixture was quenched with citric acid (pH = 6) and was extracted with 10% MeOH in DCM (3 x 30 mL). The organic layers were combined and washed with brine (50 mL), dried over sodium sulfate, filtered, and concentrated to provide **2-(4-(1,3-dioxolan-2-yl)phenyl) acetic acid (18)** (80.0 mg, 384 μmol, 23.0% over 2 steps) as an off-white solid. The product was used without further purification.
LCMS (ESI, +ve mode): Expected *m/z* for $[C_{11}H_{13}O_4]^+$ [M+H] 209.1, found 209.2.

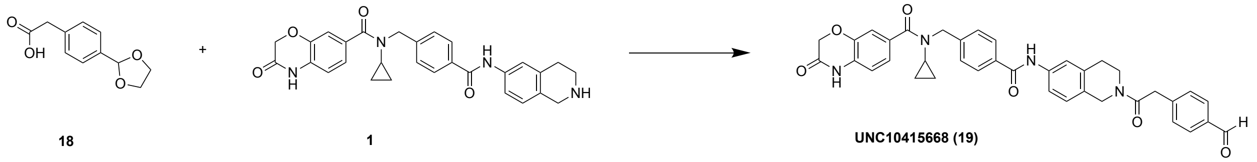

18 1 UNC10415668 (19)

N-cyclopropyl-N-(4-((2-(2-(4-formylphenyl) acetyl)-1,2,3,4-tetrahydroisoquinolin-6-yl) carbamoyl) benzyl)-3-oxo-3,4-dihydro-2H-benzo[b][1,4]oxazine-7-carboxamide (UNC10415668, or 19)

To a scintillation vial charged with a stir bar was added 2-(4-(1,3-dioxolan-2-yl) phenyl) acetic acid (18) (0.08 g, 0.38 mmol, 1.0 Eq), DMF (0.8 mL), HATU (0.22 g, 0.57 mmol, 1.5 Eq), and DIPEA (0.149 g, 1.15 mmol, 3.0 Eq). The mixture was left to stir at room temperature for 15 minutes. To the vial was then added N-cyclopropyl-3-oxo-N-(4-((1,2,3,4-tetrahydroisoquinolin-6-yl) carbamoyl) benzyl)-3,4-dihydro-2H-benzo[b] [1,4] oxazine-7-carboxamide (0.190 g, 0.38 mmol, 1.0 Eq) (1). The reaction was stirred at room temperature for 2 hours. After completion of the reaction monitored by TLC and LCMS, the reaction mixture was poured in to ice cold water. The separated solid was filtered and dried to get the pure intermediate as an off-white solid, which was used right away. To a scintillation vial charged with a stir bar was added the intermediate (0.045 g, 0.07 mmol, 1.0 Eq) followed by formic acid (0.5 mL). The reaction was allowed to stir at room temperature for 4 hours. After completion of the reaction, the reaction mixture was poured in to water, and the product was extracted with DCM (3 x 50 mL). The organic layers were combined

and washed with $NaHCO_3$ once (30 mL), dried over sodium sulfate, filtered, and concentrated *in vacuo* to provide the crude compound. The crude compound was purified by prep-HPLC (5-100% ACN in H_2O + 0.1% formic acid). The pure fractions were collected, suspended in water, flash frozen and lyophilized to dryness to give **N-cyclopropyl-N-(4-((2-(2-(4-formylphenyl) acetyl)-1,2,3,4-tetrahydroisoquinolin-6-yl) carbamoyl) benzyl)-3-oxo-3,4-dihydro-2H-benzo[b] [1,4] oxazine-7-carboxamide (UNC10415668, or 19)** (10.0 mg, 15.57 µmol, 7.83% over 2 steps) as a white solid.

^{1}H NMR (400 MHz, DMSO-d6) δ 10.60 (s, 1H), 9.99 (s, 1H), 9.95 (s, 1H), 7.94 (d, J=8.0 Hz, 2H), 7.84 (d, J=8.0 Hz, 2H), 7.61 (s, 1H), 7.55 (d, J= 8.0 Hz, 1H), 7.49 (d, J= 8.0 Hz, 2H),7.43 (d, J=8.0 Hz, 2H), 7.16-7.11 (m, 3H), 6.95 (d, J= 8.0 Hz, 1H), 4.71 (s, 2H), 4.65 (s, 2H), 4.59 (s, 2H), 3.94 (s, 2H), 3.74 (s 2H), 2.82-2.78 (m, 3H), 0.58 (d, J= 6.0 Hz, 2H),0.52 (s, 2H)

LCMS (ESI, +ve mode): Expected *m/z* for $[C_{38}H_{35}N_4O_6]^+$ [M+H] 643.2, found 643.2

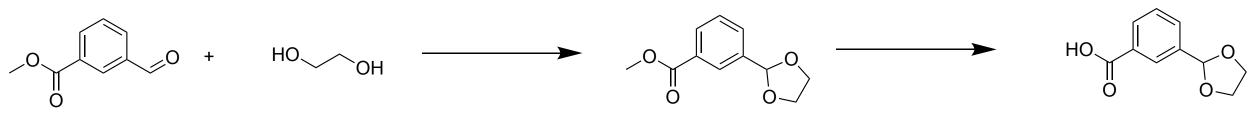

20 21

3-(1,3-dioxolan-2-yl) benzoic acid (21)

To a scintillation vial was added methyl 3-formylbenzoate (0.50 g, 3.00 mmol, 1.0 Eq), ethane-1,2-diol (0.37 g, 6.09 mmol, 2.0 Eq), Bi(OTf)3 (0.04 g, 0.06 mmol, 0.02 Eq), and Toluene (5.0 mL). The reaction was allowed to stir for 12 hours at room temperature. After completion of the reaction, the reaction mixture was poured into water and was extracted with EtOAc (3 x 50 mL). The organic layers were combined and washed with brine once (50 mL), dried over sodium sulfate, filtered, and concentrated *in vacuo* to provide the crude compound. The resulting crude compound was purified by normal phase flash column chromatography (0-100% EtOAc in Hexane). The pure product was collected and concentrated to afford methyl 3-(1,3-dioxolan-2-yl) benzoate (20) (0.30 g, 1.44 mmol, 47%) as a clear oil, which was used right away. To a stirred solution of methyl 3-(1,3-dioxolan-2-yl) benzoate (20) (0.30 g, 1.44 mmol, 1.0 Eq) in THF (1 mL), was added lithium hydroxide (0.12 g, 2.88 mmol, 2.0 Eq) in H_2O (1 mL). The reaction mixture was stirred at room temperature for 3 h. After completion of the reaction, the reaction mixture was quenched with citric acid (pH = 6) and was extracted with EtOAc (3 x 30 mL). The organic layers were combined and washed with brine (50 mL), dried over sodium sulfate, filtered, and concentrated to provide **3-(1,3-dioxolan-2-yl) benzoic acid (21)** (22.0 mg, 113.37 µmol, 36.7% over 2 steps) as a white solid.

^{1}H NMR (400 MHz, DMSO-d6) δ 13.07 (s, 1H), 8.01 (s, 1H),7.96 (d, J=7.6 Hz, 1H), 7.76 (d, J=7.6 Hz, 1H), 7.68 (d, J=7.6 Hz, 1H), 5.81 (s, 1H),4.11-4.06 (m, 2H), 3.99-3.94 (m, 2H)

LCMS (ESI, +ve mode): Expected *m/z* for $[C_{10}H_{11}O_4]^+$ [M+H] 195.1, found 195.2.

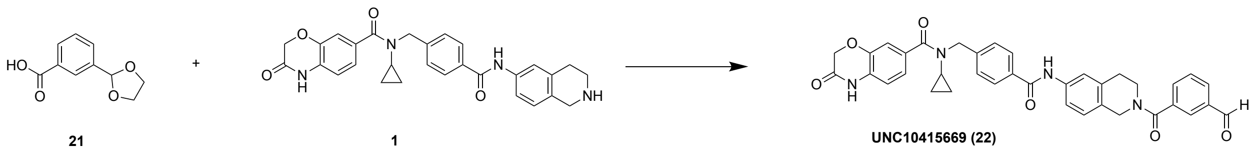

21 1 UNC10415669 (22)

N-cyclopropyl-N-(4-((2-(3-formylbenzoyl)-1,2,3,4-tetrahydroisoquinolin-6-yl) carbamoyl) benzyl)-3-oxo-3,4-dihydro-2H-benzo[b][1,4]oxazine-7-carboxamide (UNC10415669, or 22)

To a scintillation vial charged with a stir bar was added 3-(1,3-dioxolan-2-yl) benzoic acid (21) (0.08 g, 0.38 mmol, 1.0 Eq), DMF (1.0 mL), HATU (0.30 g, 0.77 mmol, 1.5 Eq), and DIPEA (0.3 mL, 1.54 mmol, 3.0 Eq). The mixture was left to stir at room temperature for 10 minutes. To the vial was then added N-cyclopropyl-3-oxo-N-(4-((1,2,3,4-tetrahydroisoquinolin-6-yl) carbamoyl) benzyl)-3,4-dihydro-2H-benzo[b] [1,4] oxazine-7-carboxamide (1) (0.10 g, 0.20 mmol, 0.4 Eq). The reaction was stirred at room temperature for 4 hours. After completion of the reaction monitored by TLC and LCMS, the reaction mixture was poured in to ice cold water and extracted with EtOAc (3 x 30 mL). The organic layers were combined and washed with brine (50 mL), dried over sodium sulfate, filtered and concentrated to get the crude intermediate. The resulting crude intermediate was purified by normal phase flash column chromatography (0-10% MeOH in DCM) to give N-(4-((2-(3-(1,3-dioxolan-2-yl) benzoyl)-1,2,3,4-tetrahydroisoquinolin-6-yl) carbamoyl) benzyl)-N-cyclopropyl-3-oxo-3,4-dihydro-2H-benzo[b] [1,4] oxazine-7-carboxamide (0.070 g, 0.148 mmol, 20%) as an off-white solid. The intermediate was used right away. To a scintillation vial charged with a stir bar was added the intermediate (0.070 g, 0.148 mmol, 1.0 Eq), followed by formic acid (1.0 mL). The reaction was allowed to stir at room temperature for 2 hours. After completion of the reaction, the reaction mixture was poured in to water, and the product was extracted with DCM (3 x 50 mL). The organic layers were combined and washed with $NaHCO_3$ once (30 mL), dried over sodium sulfate, filtered, and concentrated *in vacuo* to provide the crude compound. The crude compound was purified by prep-HPLC (5-100% ACN in H_2O + 0.1% formic acid). The pure fractions were collected, suspended in water, flash frozen and lyophilized to dryness to give **N-cyclopropyl-N-(4-((2-(3-formylbenzoyl)-1,2,3,4-tetrahydroisoquinolin-6-yl) carbamoyl) benzyl)-3-oxo-3,4-dihydro-2H-benzo[b] [1,4]oxazine-7-carboxamide (UNC10415669, or 22)** (20.0 mg, 31.84 µmol, 4.20% over 2 steps) as a white solid.

^{1}H NMR (400 MHz, DMSO-d6) δ 10.57 (s, 1H), 10.08 (s, 1H), 9.98 (s, 1H), 8.01 (d, J=1.2 Hz, 1H), 7.99-7.93 (m, 3H), 7.78 (d, J=7.6 Hz, 1H), 7.70 (t, J= 7.2 Hz, 1H), 7.64 (s, 1H), 7.56 (d, J= 8.0 Hz, 1H),7.44 (d, J=8.0 Hz, 2H), 7.17-7.10 (m, 3H), 6.95 (d, J= 8.0 Hz, 1H), 4.71 (s, 2H), 4.68 (s, 2H),

4.59 (s, 2H), 3.71 (s, 2H), 2.89 (t, J= 5.6 Hz, 2H), 2.79-2.76 (m, 1H), 0.58-0.54 (m, 2H), 0.52 (d, J= 2.8 Hz, 2H)

LCMS (ESI, +ve mode): Expected *m/z* for $[C_{37}H_{33}N_4O_6]^+$ [M+H] 629.2, found 629.0

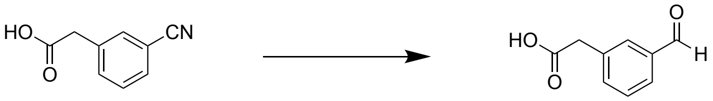

23

2-(3-formylphenyl) acetic acid (23)

To a stirred solution of 2-(3-cyanophenyl) acetic acid (0.10 g, 0.62 mmol, 1.0 Eq) in Formic acid (1.0 mL) was added Raney nickel (0.05 g). The reaction mixture was stirred at 80°C for 5 hours. Reaction progress was monitored by TLC and LCMS. After completion of the reaction monitored by TLC, the reaction mixture was filtered through celite. The filtrate was concentrated to afford **2-(3-formylphenyl) acetic acid (23)** (50.0 mg, 304.79 µmol, 49%) as a pale-yellow resin. The product was used without further purification.

^{1}H NMR (400 MHz, DMSO-d6) δ 9.99 (s, 1H), 7.79 (s, 2H), 7.61-7.53 (m, 2H), 3.77 (s, 2H)

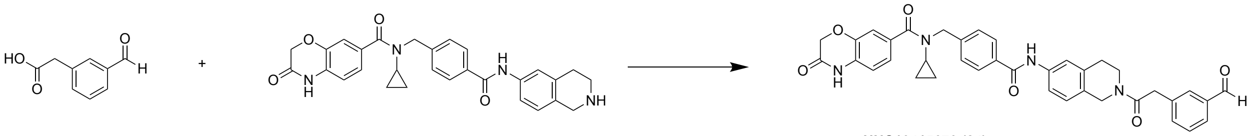

23 1 UNC10415670 (24)

N-cyclopropyl-N-(4-((2-(2-(3-formylphenyl) acetyl)-1,2,3,4-tetrahydroisoquinolin-6-yl) carbamoyl) benzyl)-3-oxo-3,4-dihydro-2H-benzo[b] [1,4] oxazine-7-carboxamide (UNC10415670, or 24)

To a scintillation vial charged with a stir bar was added 2-(3-formylphenyl) acetic acid (23) (0.05 g, 0.30 mmol, 1.0 Eq), DMF (1.0 mL), HATU (0.17 g, 0.45 mmol, 1.5 Eq), and DIPEA (0.15 mL, 0.91 mmol, 3.0 Eq). The mixture was left to stir at room temperature for 15 minutes. To the vial was then added N-cyclopropyl-3-oxo-N-(4-((1,2,3,4-tetrahydroisoquinolin-6-yl) carbamoyl) benzyl)-3,4-dihydro-2H-benzo[b] [1,4] oxazine-7-carboxamide (1) (0.060 g, 0.12 mmol, 0.4 Eq). The reaction was stirred at room temperature for 4 hours. After completion of the reaction monitored by TLC and LCMS, the reaction was poured in to water and was extracted with 10 % MeOH in DCM (3 x 50 mL). The organic layers were combined and washed with brine once (30 mL), dried over sodium sulfate, filtered, and concentrated to get the crude compound. The crude product was purified by prep-HPLC (5-100% ACN in H_2O + 0.1% formic acid) to give **N-cyclopropyl-N-(4-((2-(2-(3-formylphenyl) acetyl)-1,2,3,4-tetrahydroisoquinolin-6-yl)**

carbamoyl) benzyl)-3-oxo-3,4-dihydro-2H-benzo[b] [1,4] oxazine-7-carboxamide (UNC10415670, or 24) (8.0 mg, 12.46 µmol, 5.0%) as a white solid.

^{1}H NMR (400 MHz, DMSO-d6) δ 10.62 (d, J= 5.2 Hz, 1H), 10.0 (s, 1H), 9.95 (s, 1H), 7.94 (d, J=8.4 Hz, 2H), 7.78 (d, J=8.4 Hz, 2H), 7.61-7.53 (m, 4H), 7.43 (d, J= 8.4 Hz, 2H), 7.17-7.10 (m, 3H), 6.96-6.94 (m, 1H), 4.71 (s, 2H), 4.65 (s, 2H), 4.59 (s, 2H), 3.94 (s, 2H), 3.76 (s 2H), 2.82-2.76 (m, 3H), 0.58 (d, J= 4.4 Hz, 2H),0.52 (s, 2H)

LCMS (ESI, +ve mode): Expected *m/z* for $[C_{38}H_{35}N_4O_6]^+$ [M+H] 643.2, found 643.0

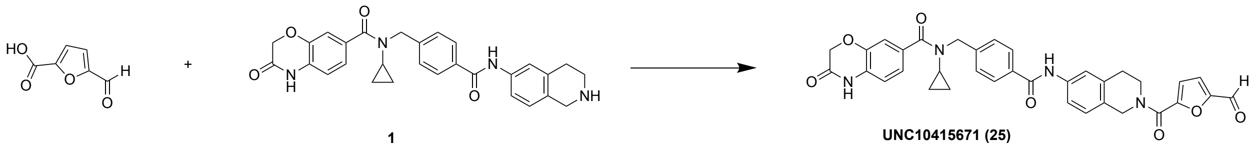

1

UNC10415671 (25)

N-cyclopropyl-N-(4-((2-(5-formylfuran-2-carbonyl)-1,2,3,4-tetrahydroisoquinolin-6-yl) carbamoyl) benzyl)-3-oxo-3,4-dihydro-2H-benzo[b] [1,4] oxazine-7-carboxamide (UNC10415671, or 25)

To a scintillation vial charged with a stir bar was added 5-formylfuran-2-carboxylic acid (0.021 g, 0.15 mmol, 1.5 Eq), DMF (1.0 mL), HATU (0.057 g, 0.15 mmol, 1.5 Eq), and DIPEA (0.051 g, 0.30 mmol, 3.0 Eq). The mixture was left to stir at room temperature for 15 minutes. To the vial was then added N-cyclopropyl-3-oxo-N-(4-((1,2,3,4-tetrahydroisoquinolin-6-yl) carbamoyl) benzyl)-3,4-dihydro-2H-benzo[b] [1,4] oxazine-7-carboxamide (1) (0.060 g, 0.12 mmol, 0.4 Eq). The reaction was stirred at room temperature for 1 hour. The reaction was poured into water to give the solid as the crude product. The crude product was purified by prep-HPLC (5-100% ACN in H_2O + 0.1% formic acid) to give **N-cyclopropyl-N-(4-((2-(5-formylfuran-2-carbonyl)-1,2,3,4-tetrahydroisoquinolin-6-yl) carbamoyl) benzyl)-3-oxo-3,4-dihydro-2H-benzo[b] [1,4] oxazine-7-carboxamide (UNC10415671, or 25)** (15.0 mg, 24.26 µmol, 24.0%) as a white solid.

^{1}H NMR (400 MHz, DMSO-d6) δ 10.62 (s, 1H), 9.99 (s, 1H), 9.74 (s, 1H), 7.94 (d, J=8.0 Hz, 2H), 7.66 (s, 1H), 7.60-7.56 (m, 2H), 7.44 (d, J= 8.4 Hz, 2H), 7.20-7.14 (m, 3H), 7.11 (s, 1H), 6.95 (d, J=8.0 Hz, 1H), 4.79 (s, 2H), 4.65 (s, 2H), 4.71 (s, 2H), 4.59 (s, 2H), 3.89 (t, J= 5.6 Hz, 2H), 2.95 (t, J= 5.6 Hz, 2H), 2.78 (d, J= 3.2 Hz, 1H), 0.58 (d, J= 6.0 Hz, 2H), 0.52 (s, 2H),

LCMS (ESI, +ve mode): Expected *m/z* for $[C_{35}H_{31}N_4O_7]^+$ [M+H] 619.2, found 619.0

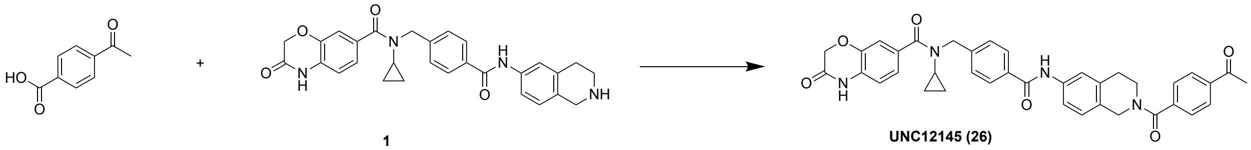

1

UNC12145 (26)

N-(4-((2-(4-acetylbenzoyl)-1,2,3,4-tetrahydroisoquinolin-6-yl)carbamoyl)benzyl)-N-cyclopropyl-3-oxo-3,4-dihydro-2H-benzo[b][1,4]oxazine-7-carboxamide (UNC12145, or 26)

To a scintillation vial was added 4-acetylbenzoic acid (12.9 mg, 1.3 Eq, 78.5 μmol), N-cyclopropyl-3-oxo-N-(4-((1,2,3,4-tetrahydroisoquinolin-6-yl)carbamoyl)benzyl)-3,4-dihydro-2H-benzo[b][1,4]oxazine-7-carboxamide (30.0 mg, 1 Eq, 60.4 μmol), EDC (23.2 mg, 2 Eq, 121 μmol), DMAP (14.8 mg, 2 Eq, 121 μmol), and DMF (1.0 mL). The reaction mixture was stirred at room temperature for 24 hours. After completion of the reaction, the reaction mixture was concentrated with a steady nitrogen blow. The resulting crude product was purified by reverse phase flash chromatography (10-100% ACN in neutral H_2O). The crude fractions were collected and concentrated *in vacuo*. The crude product was purified by prep-HPLC (5-100% ACN in H_2O + 0.05% TFA) to give **N-(4-((2-(4-acetylbenzoyl)-1,2,3,4-tetrahydroisoquinolin-6-yl)carbamoyl)benzyl)-N-cyclopropyl-3-oxo-3,4-dihydro-2H-benzo[b][1,4]oxazine-7-carboxamide (UNC12145, or 26)** (22.85 mg, 35.55 μmol, 58.8 %) as a white solid.

^{1}H NMR (500 MHz, DMSO) δ 10.87 (s, 1H), 10.19 (s, 0H), 8.04 (d, 2H), 7.94 (d, *J* = 7.7 Hz, 2H), 7.66 (s, 1H), 7.63 – 7.41 (m, 5H), 7.25 (d, *J* = 8.4 Hz, 1H), 7.18 (d, *J* = 8.0 Hz, 1H), 7.14 (s, 1H), 6.92 (d, *J* = 8.0 Hz, 1H), 4.74 (d, 3H), 4.61 (s, 2H), 4.49 (s, 1H), 3.95 – 3.49 (m, 2H), 2.87 (d, 2H), 2.79 (s, 1H), 2.62 (s, 3H), 0.54 (s, 2H), 0.47 (s, 2H).

LCMS (ESI, +ve mode): Expected *m/z* for $[C_{38}H_{35}N_4O_6]^+$ [M+H] 643.2, found 643.3

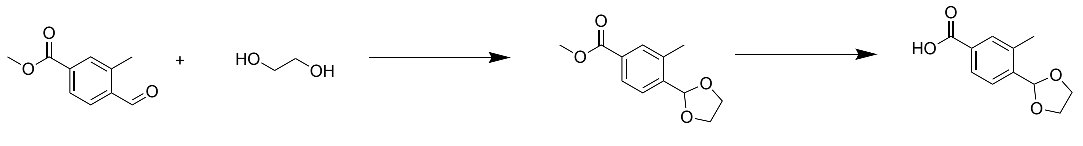

27 28

4-(1,3-dioxolan-2-yl)-3-methylbenzoic acid (28)

To a scintillation vial was added methyl 4-formyl-3-methylbenzoate (50.0 mg, 1 Eq, 281 μmol), ethane-1,2-diol (87.1 mg, 78.5 μL, 5.0 Eq, 1.40 mmol), PTSA (4.83 mg, 0.1 Eq, 28.1 μmol), and Toluene (3.0 mL). The reaction was allowed to stir for 16 hours at room temperature. After completion of the reaction, the reaction mixture was concentrated with a steady nitrogen blow. The resulting crude product was purified by normal phase chromatography (0-100% EtOAc in Hexane) to give methyl 4-(1,3-dioxolan-2-yl)-3-methylbenzoate (27) (62.8 mg, 0.25 mmol, 91 %) as a clear oil, which was used right away. To a stirred solution of methyl 4-(1,3-dioxolan-2-yl)-3-methylbenzoate (27) (62.8 mg, 0.25 mmol, 91 %) in THF (1 mL), was added lithium hydroxide (33.8 mg, 5 Eq, 1.41 mmol) in H_2O (1 mL). The reaction mixture was stirred at room temperature for 16 hours. After completion of the reaction, the reaction mixture was quenched with 1M HCl (pH was adjusted to 6) and was extracted with DCM (3 x 30 mL). The organic layers were

combined and washed with brine once (50 mL), dried over sodium sulfate, filtered, and concentrated *in vacuo* to provide the **4-(1,3-dioxolan-2-yl)-3-methylbenzoic acid (28)** (28.7 mg, 138 µmol, 44.4 % over 2 steps) as an off-white solid. The product was used without further purification.

LCMS (ESI, +ve mode): Expected *m/z* for $[C_{11}H_{13}O_4]^+$ [M+H] 209.1, found 209.2.

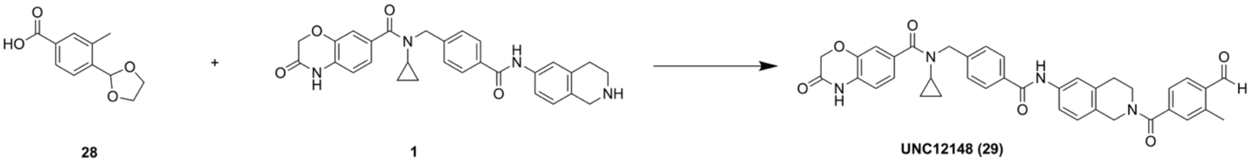

28 1 UNC12148 (29)

N-cyclopropyl-N-(4-((2-(4-formyl-3-methylbenzoyl)-1,2,3,4-tetrahydroisoquinolin-6-yl)carbamoyl)benzyl)-3-oxo-3,4-dihydro-2H-benzo[b][1,4]oxazine-7-carboxamide (UNC12148, or 29)

To a scintillation vial charged with a stir bar was added 4-(1,3-dioxolan-2-yl)-3-methylbenzoic acid (28) (25.2 mg, 1.0 Eq, 121 µmol, DMF (1.0 mL), HATU (59.7 mg, 1.3 Eq, 157 µmol), and DIPEA (109 mg, 147 µL, 7 Eq, 846 µmol). The mixture was left to stir at room temperature for 60 minutes. To the vial was then added N-cyclopropyl-3-oxo-N-(4-((1,2,3,4-tetrahydroisoquinolin-6-yl) carbamoyl) benzyl)-3,4-dihydro-2H-benzo[b] [1,4] oxazine-7-carboxamide (1) (60.0 mg, 121 µmol, 1.0 Eq). The reaction was stirred at room temperature for 24 hours. After completion of the reaction, the reaction mixture was concentrated with a steady nitrogen blow. The resulting crude product was purified by reverse phase flash chromatography (10-100% ACN in neutral H_2O). The pure fraction was collected and concentrated *in vacuo*. The intermediate N-(4-((2-(4-(1,3-dioxolan-2-yl)-3-methylbenzoyl)-1,2,3,4-tetrahydroisoquinolin-6-yl)carbamoyl)benzyl)-N-cyclopropyl-3-oxo-3,4-dihydro-2H-benzo[b][1,4]oxazine-7-carboxamide (43.1 mg, 121 µmol, 51.9%) was obtained as an off-white solid. The intermediate was dissolved in formic acid (1.0 mL) and allowed to stir at room temperature for 24 hours. After completion of the reaction, the product was concentrated with a steady nitrogen blow. The product was purified by prep-HPLC (5-100% ACN in neutral H_2O) to give **N-cyclopropyl-N-(4-((2-(4-formyl-3-methylbenzoyl)-1,2,3,4-tetrahydroisoquinolin-6-yl)carbamoyl)benzyl)-3-oxo-3,4-dihydro-2H-benzo[b][1,4]oxazine-7-carboxamide (UNC12148, or 29)** (3.97 mg, 6.18 µmol, 5.11% over 2 steps) as a yellow solid.

^{1}H NMR (500 MHz, MeOD) δ 10.31 (s, 1H), 7.93 (d, *J* = 7.7 Hz, 3H), 7.75 – 7.33 (m, 6H), 7.26 (t, *J* = 8.7 Hz, 1H), 7.22 – 7.14 (m, 2H), 6.96 (d, *J* = 8.1 Hz, 1H), 4.82 (s, 2H), 4.62 (s, 2H), 4.57 (d, *J* = 10.3 Hz, 2H), 3.78 (d, 2H), 2.95 (d, 2H), 2.82 (s, 1H), 2.72 (d, *J* = 6.3 Hz, 2H), 2.43 (s, 1H), 0.65 (s, 2H), 0.56 (s, 2H).

LCMS (ESI, +ve mode): Expected *m/z* for $[C_{38}H_{35}N_4O_6]^+$ [M+H] 643.2, found 643.3

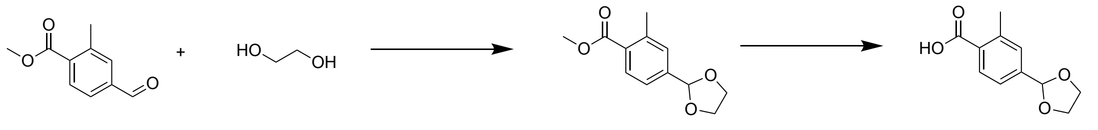

30

31

4-(1,3-dioxolan-2-yl)-2-methylbenzoic acid (31)

To a scintillation vial was added methyl 4-formyl-2-methylbenzoate (50.0 mg, 1 Eq, 281 µmol), ethane-1,2-diol (87.1 mg, 78.5 µL, 5.0 Eq, 1.40 mmol), PTSA (5.37 mg, 90% Wt, 0.1 Eq, 28.1 µmol), and Toluene (3.0 mL). The reaction was allowed to stir for 16 hours at room temperature. After completion of the reaction, the reaction mixture was concentrated with a steady nitrogen blow. The resulting crude product was purified by normal phase chromatography (0-100% EtOAc in Hexane) to give methyl 4-(1,3-dioxolan-2-yl)-2-methylbenzoate (30) (56.7 mg, 255 µmol, 90.9 %) as a clear oil, which was used right away. To a stirred solution of methyl 4-(1,3-dioxolan-2-yl)-2-methylbenzoate (30) (56.7 mg, 255 µmol, 90.9 %) in THF (1 mL), was added lithium hydroxide (30.6 mg, 5 Eq, 1.28 mmol) in H_2O (1 mL). The reaction mixture was stirred at room temperature for 16 h. After completion of the reaction, the reaction mixture was quenched with 1M HCl (pH was adjusted to 4) and was extracted with DCM (3 x 30 mL). The organic layers were combined and washed with brine once (50 mL), dried over sodium sulfate, filtered, and concentrated *in vacuo* to provide **4-(1,3-dioxolan-2-yl)-2-methylbenzoic acid (31)** (48.7 mg, 234 µmol, 83.4 % over 2 steps) as an off-white solid. The product was used without further purification. LCMS (ESI, +ve mode): Expected *m/z* for $[C_{11}H_{13}O_4]^+$ [M+H] 209.1, found 209.1.

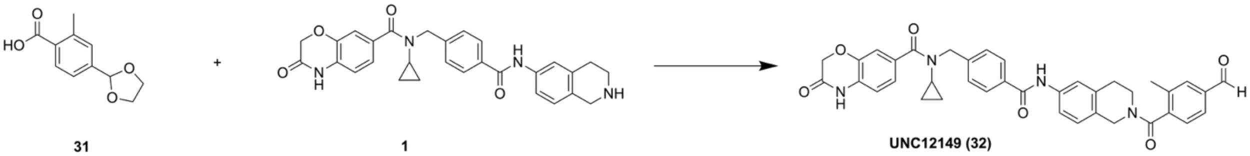

31

1

UNC12149 (32)

N-cyclopropyl-N-(4-((2-(4-formyl-2-methylbenzoyl)-1,2,3,4-tetrahydroisoquinolin-6-yl)carbamoyl)benzyl)-3-oxo-3,4-dihydro-2H-benzo[b][1,4]oxazine-7-carboxamide (UNC12149, or 32)

To a scintillation vial charged with a stir bar was added 4-(1,3-dioxolan-2-yl)-2-methylbenzoic acid (31) (16.8 mg, 1 Eq, 80.6 µmol), HATU (39.8 mg, 1.3 Eq, 105 µmol), DIPEA (72.9 mg, 98.2 µL, 7 Eq, 564 µmol), and DMF (1.0 mL). The mixture was left to stir at room temperature for 60 minutes. To the vial was then added N-cyclopropyl-3-oxo-N-(4-((1,2,3,4-tetrahydroisoquinolin-6-yl) carbamoyl) benzyl)-3,4-dihydro-2H-benzo[b] [1,4] oxazine-7-carboxamide (1) (40.0 mg, 1 Eq, 80.6 µmol). The reaction was stirred at room temperature for 16 hours. After completion of the reaction, the reaction mixture was concentrated with a steady nitrogen blow. The resulting crude

product was purified by reverse phase flash chromatography (10-100% ACN in neutral H_2O). The intermediate was collected and concentrated *in vacuo* as an off-white solid. The intermediate was used right away. The intermediate was dissolved in formic acid (1.0 mL) and allowed to stir at room temperature for 24 hours. After completion of the reaction, the product was concentrated with a steady nitrogen blow. The product was purified by prep-HPLC (5-100% ACN in neutral H_2O) to give **N-cyclopropyl-N-(4-((2-(4-formyl-2-methylbenzoyl)-1,2,3,4-tetrahydroisoquinolin-6-yl)carbamoyl)benzyl)-3-oxo-3,4-dihydro-2H-benzo[b][1,4]oxazine-7-carboxamide (UNC12149, or 32)** (15.45 mg, 24.04 µmol, 29.8% over 2 steps) as a white solid. ^{1}H NMR (400 MHz, $CDCl_3$) δ 10.01 (s, 1H), 8.68 (s, 1H), 8.18 (d, *J* = 3.7 Hz, 1H), 7.84 (dd, *J* = 8.2, 2.8 Hz, 2H), 7.81 – 7.72 (m, 2H), 7.73 – 7.56 (m, 1H), 7.46 – 7.30 (m, 4H), 7.21 – 7.09 (m, 3H), 6.83 (dd, *J* = 16.5, 8.2 Hz, 1H), 5.13 – 4.81 (m, 1H), 4.77 (s, 2H), 4.62 (s, 2H), 4.30 (d, *J* = 14.9 Hz, 1H), 4.03 (d, *J* = 104.1 Hz, 1H), 3.45 (s, 1H), 2.98 (s, 1H), 2.80 (s, 1H), 2.71 – 2.58 (m, 1H), 2.40 (s, 2H), 2.30 (s, 1H), 0.63 (s, 2H), 0.51 (s, 2H).

LCMS (ESI, +ve mode): Expected *m/z* for $[C_{38}H_{35}N_4O_6]^+$ [M+H] 643.2, found 643.3.

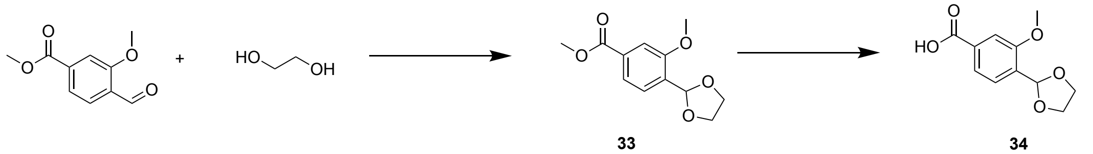

4-(1,3-dioxolan-2-yl)-3-methoxybenzoic acid (34)

To a scintillation vial was added 4-formyl-3-methoxybenzoate (67.5 mg, 1 Eq, 348 µmol), ethane-1,2-diol (108 mg, 97.2 µL, 5.0 Eq, 1.74 mmol), PTSA (6.65 mg, 90% Wt, 0.1 Eq, 34.8 µmol), and Toluene (3.0 mL). The reaction was allowed to stir for 16 hours at room temperature. After completion of the reaction, the reaction mixture was concentrated with a steady nitrogen blow. The resulting crude product was purified by normal phase chromatography (0-100% EtOAc in Hexane) to give methyl 4-(1,3-dioxolan-2-yl)-3-methoxybenzoate (33) (65.4 mg, 275 µmol, 79.0 %) as a clear oil, which was used right away. To a stirred solution of methyl 4-(1,3-dioxolan-2-yl)-3-methoxybenzoate (33) (65.4 mg, 275 µmol, 79.0 %) in THF (1 mL), was added lithium hydroxide (32.9 mg, 5 Eq, 1.37 mmol) in H_2O (1 mL). The reaction mixture was stirred at room temperature for 16 hours. After completion of the reaction, the reaction mixture was quenched with 1M HCl (pH was adjusted to 4) and was extracted with DCM (3 x 30 mL). The organic layers were combined and washed with brine once (50 mL), dried over sodium sulfate, filtered, and concentrated *in vacuo* to provide **4-(1,3-dioxolan-2-yl)-3-methoxybenzoic acid (34)** (53.7 mg, 240 µmol, 68.9 % over 2 steps) as an off-white solid. The product was used without further purification.

LCMS (ESI, +ve mode): Expected *m/z* for $[C_{11}H_{13}O_5]^+$ [M+H] 225.1, found 225.1.

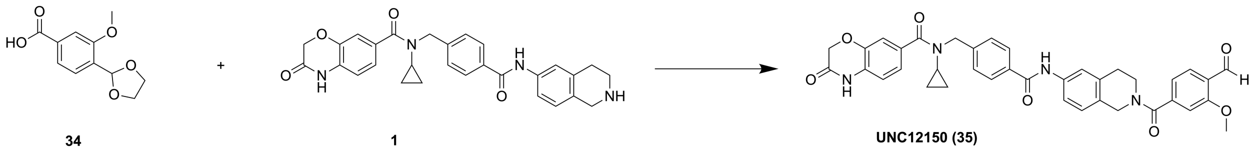

34 1 UNC12150 (35)

N-cyclopropyl-N-(4-((2-(4-formyl-3-methoxybenzoyl)-1,2,3,4-tetrahydroisoquinolin-6-yl)carbamoyl)benzyl)-3-oxo-3,4-dihydro-2H-benzo[b][1,4]oxazine-7-carboxamide (UNC12150, or 35)

To a scintillation vial charged with a stir bar was added 44-(1,3-dioxolan-2-yl)-3-methoxybenzoic acid (34) (45.15 mg, 1 Eq, 201.4 μmol), HATU (99.55 mg, 1.3 Eq, 261.8 μmol), DIPEA (182.2 mg, 246 μL, 7 Eq, 1.410 mmol), and DMF (2.0 mL). The mixture was left to stir at room temperature for 60 minutes. To the vial was then added N-cyclopropyl-3-oxo-N-(4-((1,2,3,4-tetrahydroisoquinolin-6-yl) carbamoyl) benzyl)-3,4-dihydro-2H-benzo[b] [1,4] oxazine-7-carboxamide (1) (100.0 mg, 1 Eq, 201.4 μmol). The reaction was stirred at room temperature for 16 hours. After completion of the reaction, the reaction mixture was concentrated with a steady nitrogen blow. The resulting crude product was purified by reverse phase flash chromatography (10-100% ACN in neutral H_2O). The intermediate was collected and concentrated *in vacuo* as an off-white solid. The intermediate was used right away. The intermediate was dissolved in formic acid (1.0 mL) and allowed to stir at room temperature for 3 hours. After completion of the reaction, the product was concentrated with a steady nitrogen blow. The product was purified by prep-HPLC (5-100% ACN in neutral H_2O) to give **N-cyclopropyl-N-(4-((2-(4-formyl-3-methoxybenzoyl)-1,2,3,4-tetrahydroisoquinolin-6-yl)carbamoyl)benzyl)-3-oxo-3,4-dihydro-2H-benzo[b][1,4]oxazine-7-carboxamide (UNC12150, or 35)** (16.99 mg, 25.79 μmol, 12.81% over 2 steps) as a white solid.

^{1}H NMR (500 MHz, MeOD) δ 10.45 (d, J = 0.9 Hz, 1H), 7.93 (d, J = 7.8 Hz, 2H), 7.87 (dd, J = 7.9, 2.9 Hz, 1H), 7.66 – 7.42 (m, 4H), 7.30 – 7.00 (m, 5H), 6.96 (d, J = 8.1 Hz, 1H), 4.82 (s, 2H), 4.62 (s, 2H), 4.58 (s, 2H), 4.02 – 3.95 (m, 3H), 3.92 – 3.56 (m, 2H), 3.10 – 2.86 (m, 2H), 2.82 (s, 1H), 0.65 (s, 2H), 0.56 (s, 2H).

LCMS (ESI, +ve mode): Expected *m/z* for $[C38H35N4O7]^+$ [M+H] 659.2, found 659.2

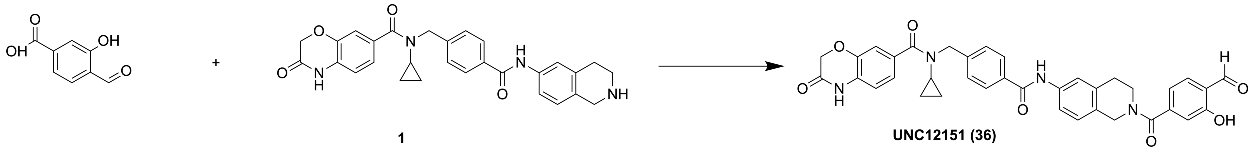

1 UNC12151 (36)

N-cyclopropyl-N-(4-((2-(4-formyl-3-hydroxybenzoyl)-1,2,3,4-tetrahydroisoquinolin-6-yl)carbamoyl)benzyl)-3-oxo-3,4-dihydro-2H-benzo[b][1,4]oxazine-7-carboxamide (UNC12151, or 36)

To a scintillation vial charged with a stir bar was added 4-formyl-3-hydroxybenzoic acid (8.36 mg, 1 Eq, 50.3 µmol), HATU (24.9 mg, 1.3 Eq, 65.4 µmol), N-ethyl-N-isopropylpropan-2-amine (45.5 mg, 61.4 µL, 7 Eq, 352 µmol), and DMF (1.0 mL). The mixture was left to stir at room temperature for 60 minutes. To the vial was then added N-cyclopropyl-3-oxo-N-(4-((1,2,3,4-tetrahydroisoquinolin-6-yl) carbamoyl) benzyl)-3,4-dihydro-2H-benzo[b] [1,4] oxazine-7-carboxamide (1) (25.0 mg, 1 Eq, 50.3 µmol). The reaction was stirred at room temperature for 24 hours. After completion of the reaction, the reaction mixture was concentrated with a steady nitrogen blow. The resulting crude product was purified by reverse phase flash chromatography (10-100% ACN in H_2O + 0.1% TFA). The product was collected and concentrated *in vacuo* as an off-white solid. The product was purified by prep-HPLC (5-100% ACN in H_2O + 0.05% TFA) to give **N-cyclopropyl-N-(4-((2-(4-formyl-3-hydroxybenzoyl)-1,2,3,4-tetrahydroisoquinolin-6-yl)carbamoyl)benzyl)-3-oxo-3,4-dihydro-2H-benzo[b][1,4]oxazine-7-carboxamide (UNC12151, or 36)** (0.65 mg, 1.0 µmol, 2.0 %) as a white solid.

^{1}H NMR (400 MHz, MeOD) δ 7.94 (d, J = 8.0 Hz, 2H), 7.62 – 7.42 (m, 5H), 7.27 – 7.14 (m, 3H), 7.00 – 6.85 (m, 3H), 4.83 (s, 2H), 4.62 (s, 2H), 4.60 (s, 2H), 3.82 (d, 2H), 3.06 – 2.87 (m, 2H), 2.82 (s, 1H), 0.65 (s, 2H), 0.56 (s, 2H).

LCMS (ESI, +ve mode): Expected *m/z* for $[C_{37}H_{33}N_4O_7]^+$ [M+H] 645.2, found 645.3

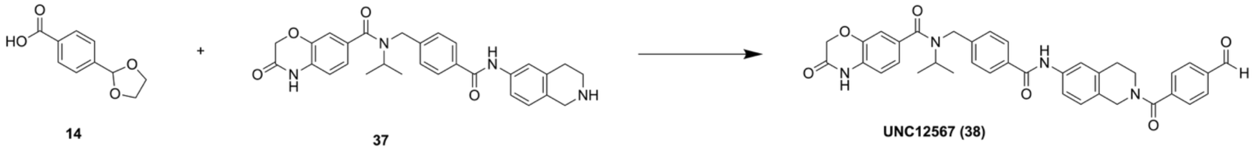

14 37 UNC12567 (38)

N-(4-((2-(4-formylbenzoyl)-1,2,3,4-tetrahydroisoquinolin-6-yl)carbamoyl)benzyl)-N-isopropyl-3-oxo-3,4-dihydro-2H-benzo[b][1,4]oxazine-7-carboxamide (UNC12567, or 38)

To a scintillation vial charged with a stir bar was added **14** (7.79 mg, 1 Eq, 40.31 µmol), HATU (19.8 mg, 1.3 Eq, 52.1 µmol), N-ethyl-N-isopropylpropan-2-amine (36.3 mg, 48.9 µL, 7 Eq, 281 µmol), and DMF (1.0 mL). The mixture was left to stir at room temperature for 30 minutes. To the vial was then added *N*-isopropyl-3-oxo-*N*-(4-((1,2,3,4-tetrahydroisoquinolin-6-yl)carbamoyl)benzyl)-3,4-dihydro-2*H*-benzo[*b*][1,4]oxazine-7-carboxamide (37) (20.0 mg, 1 Eq,

40.1 μmol). The reaction was stirred at room temperature for 24 hours. After completion of the reaction, the reaction mixture was concentrated with a steady nitrogen blow. The resulting crude intermediate was purified by reverse phase flash chromatography (10-100% ACN in neutral H_2O). The product was collected and concentrated *in vacuo* as an off-white solid. The intermediate was purified by prep-HPLC (5-100% ACN in neutral H_2O). The purified intermediate was dissolved in formic acid (1.0 mL) and allowed to stir at room temperature for 3 hours. After completion of the reaction, the product was concentrated with a steady nitrogen blow. The product was purified by prep-HPLC (5-100% ACN in neutral H_2O) to give **N-(4-((2-(4-formylbenzoyl)-1,2,3,4-tetrahydroisoquinolin-6-yl)carbamoyl)benzyl)-N-isopropyl-3-oxo-3,4-dihydro-2H-benzo[b][1,4]oxazine-7-carboxamide (UNC12567, or 38)** (6.3 mg, 10 μmol, 25 %) as a white solid.

^{1}H NMR (400 MHz, MeOD) δ 10.09 (s, 1H), 8.06 (d, *J* = 7.7 Hz, 1H), 7.92 (d, *J* = 7.9 Hz, 2H), 7.77 – 7.39 (m, 7H), 7.27 (d, *J* = 8.4 Hz, 1H), 7.17 – 6.86 (m, 3H), 4.76 (s, 2H), 4.59 (s, 4H), 4.26 (s, 1H), 4.03 (d, *J* = 5.3 Hz, 1H), 3.74 – 3.63 (m, 1H), 3.09 – 2.87 (m, 2H), 1.36 – 1.12 (s, 6H).

LCMS (ESI, +ve mode): Expected *m/z* for $[C_{37}H_{33}N_4O_7]^+$ [M+H] 631.2, found 631.2

NMR Spectra

(2)

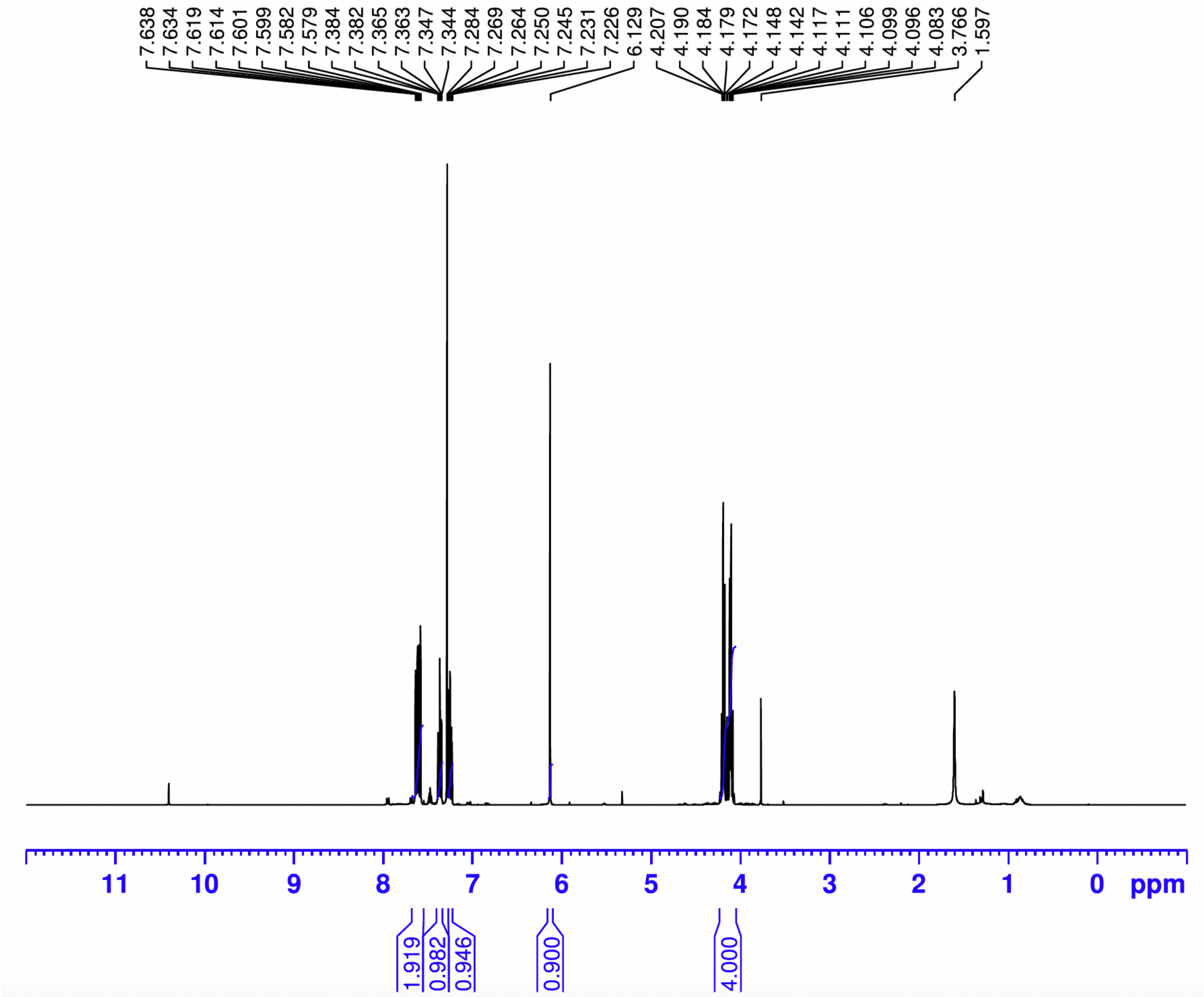

Supplementary Fig. 10 | NMR spectrum for compound 2

(3)

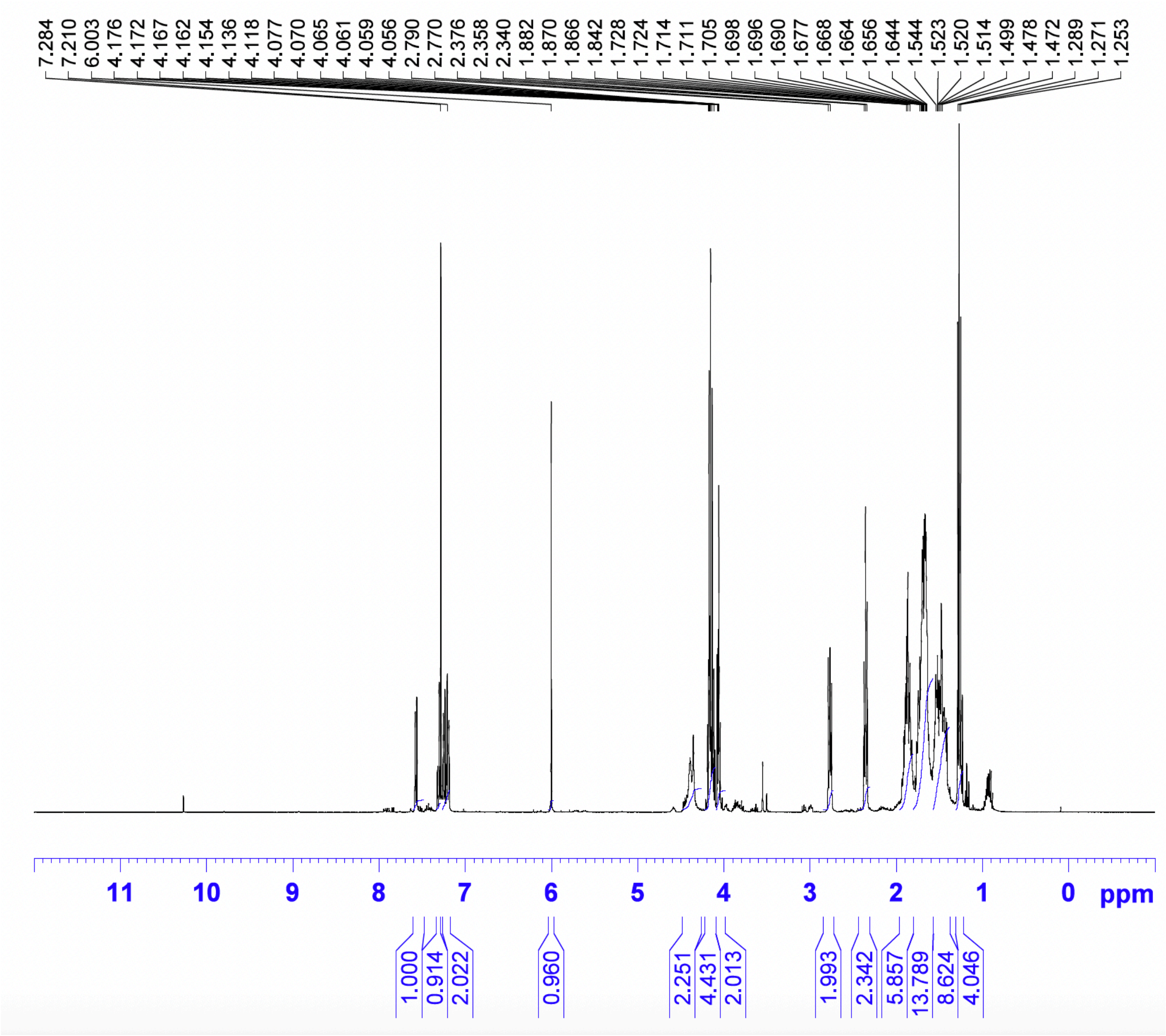

Supplementary Fig. 11 | NMR spectrum for compound 3

(4)

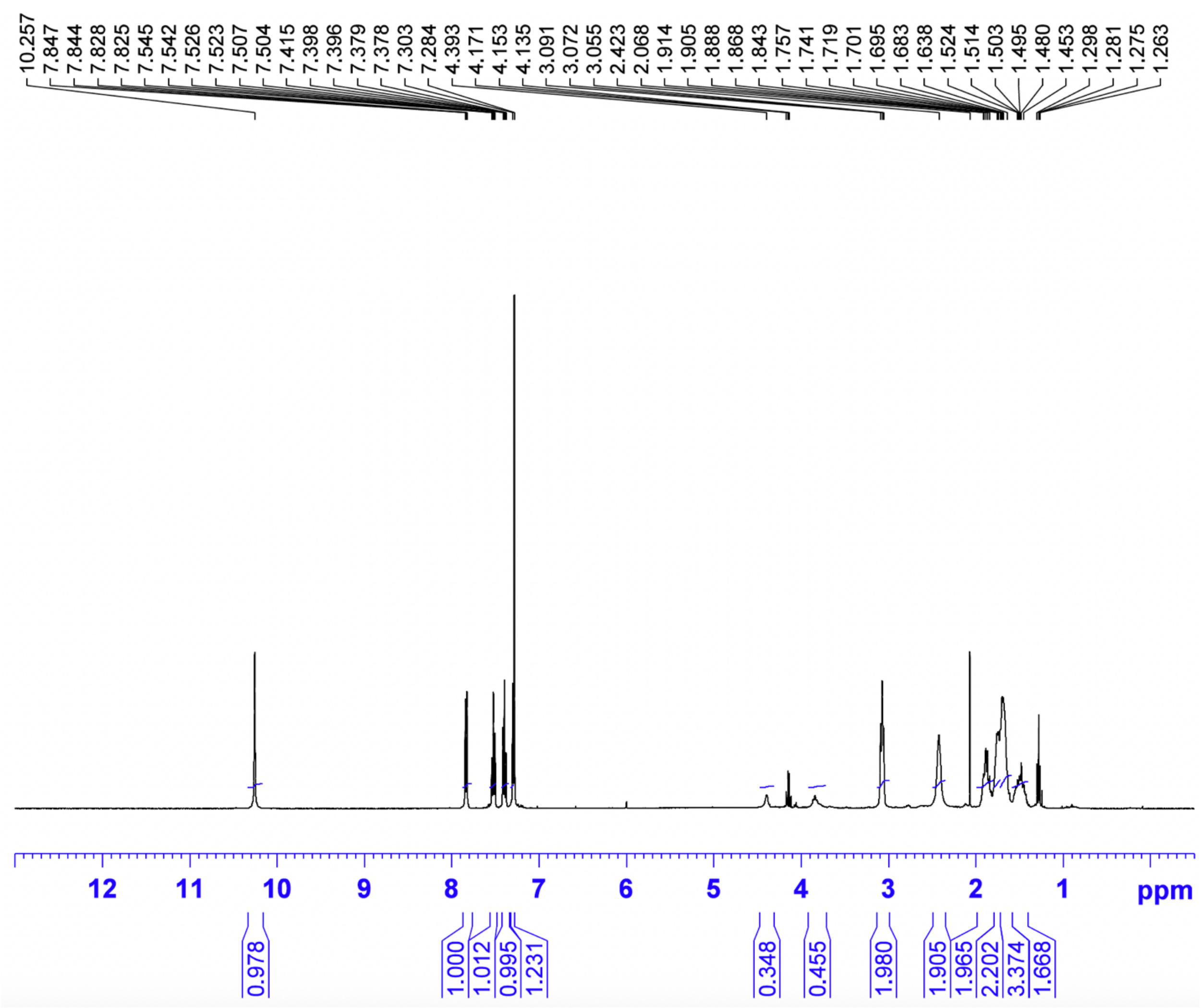

Supplementary Fig. 12 | NMR spectrum for compound 4

UNC10413724 (5)

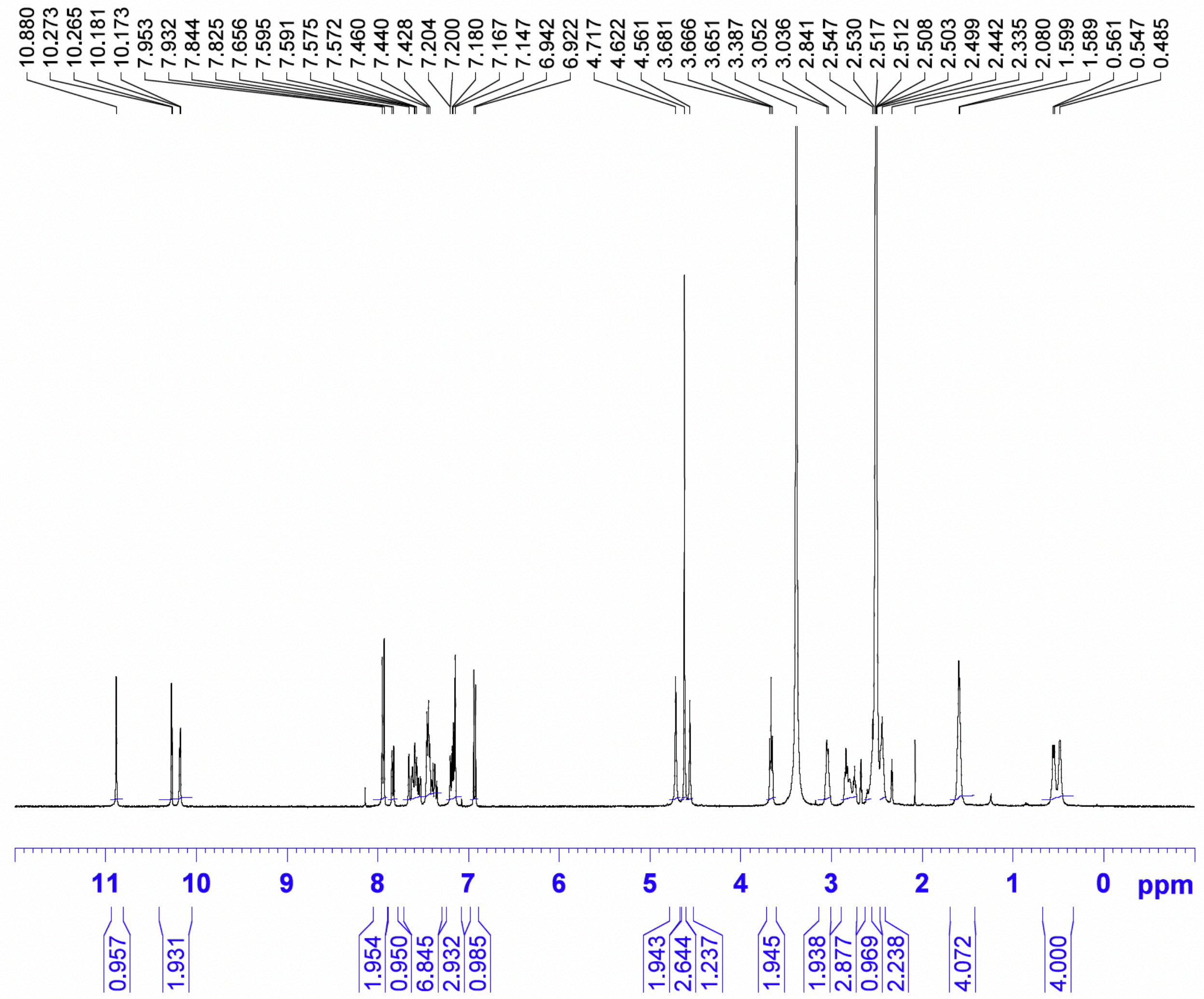

Supplementary Fig. 13 | NMR spectrum for compound 5

(6)

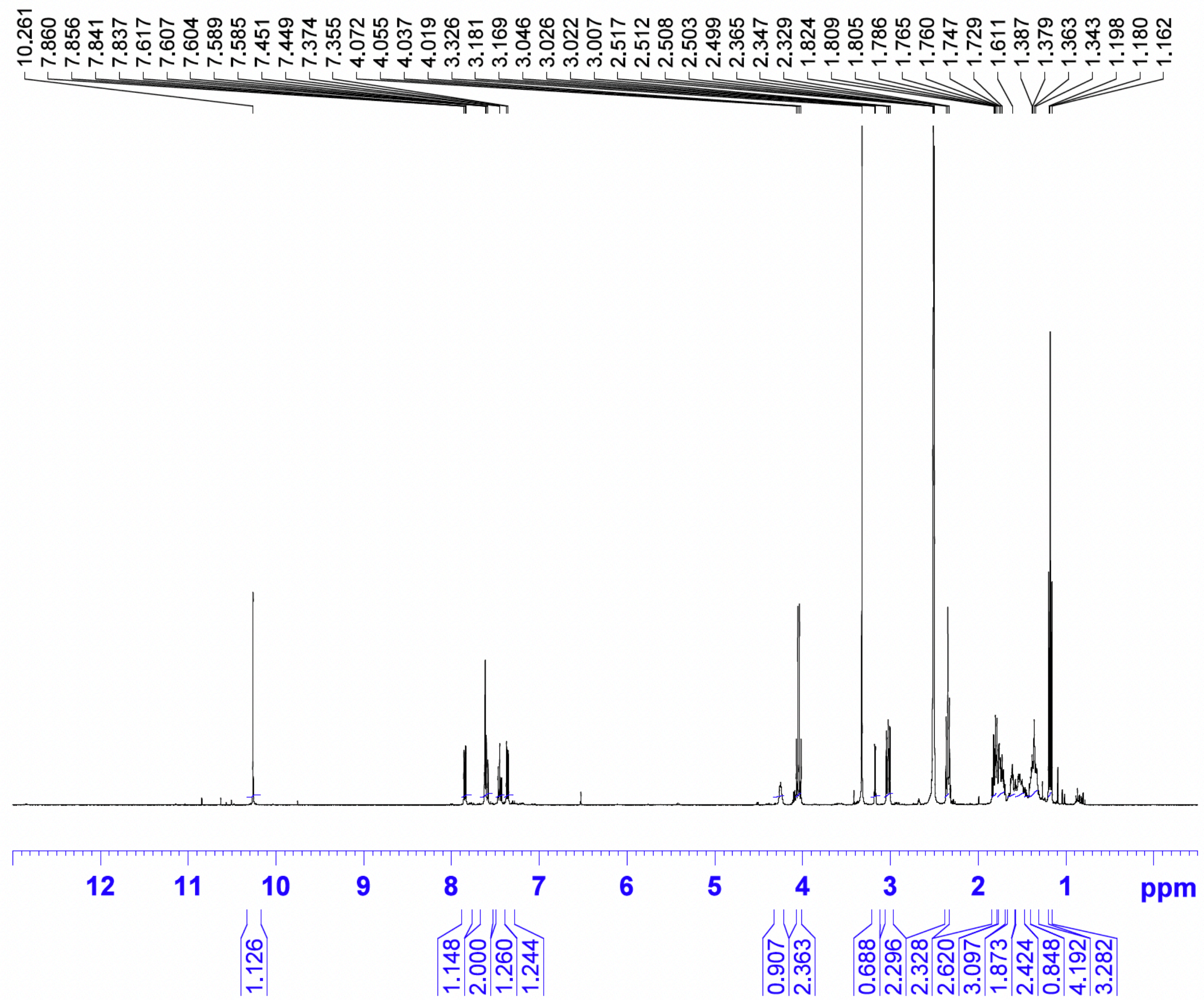

Supplementary Fig. 14 | NMR spectrum for compound 6

UNC10413728 (8)

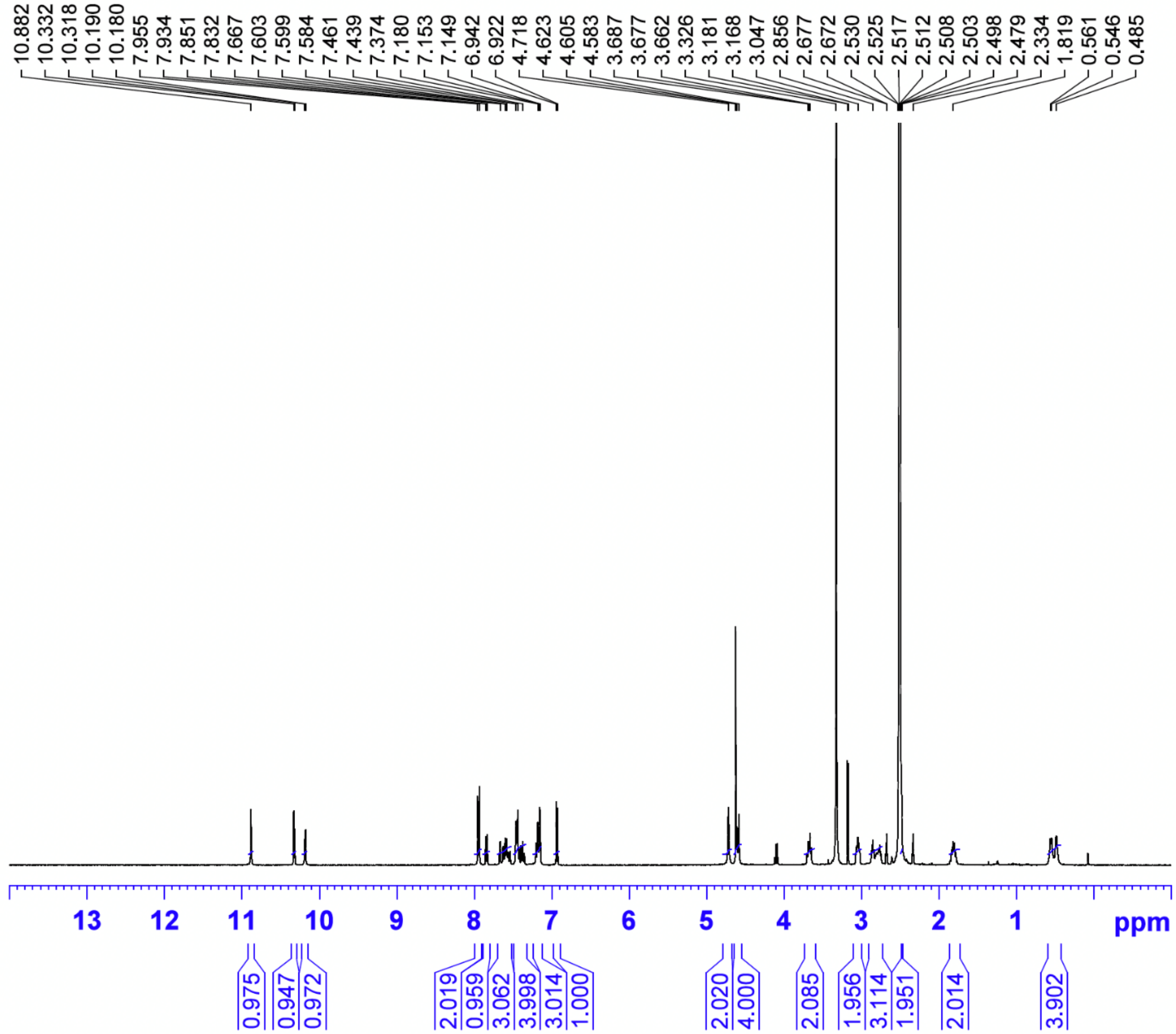

Supplementary Fig. 15 | NMR spectrum for compound 8

(9)

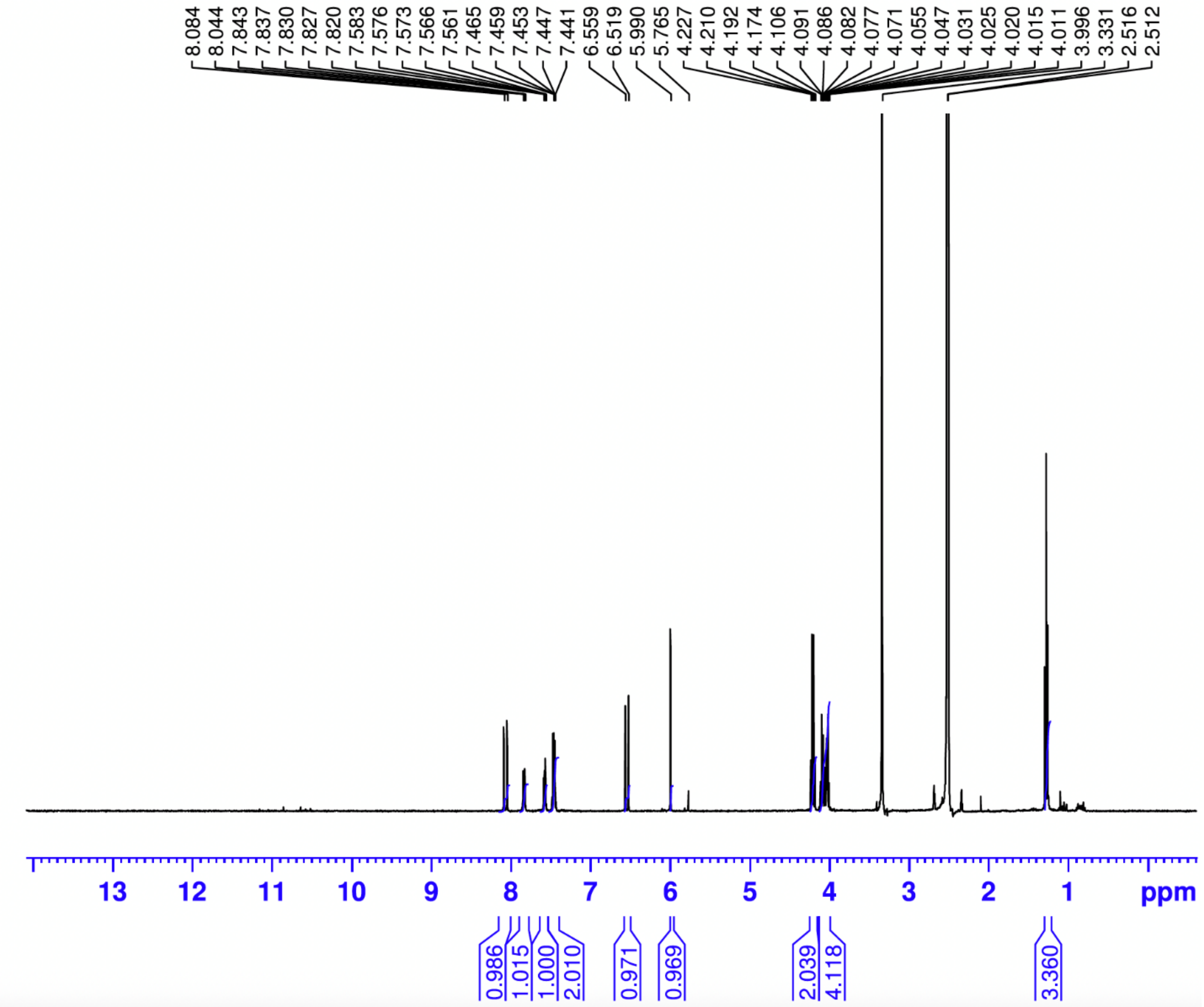

Supplementary Fig. 16 | NMR spectrum for compound 9

(10)

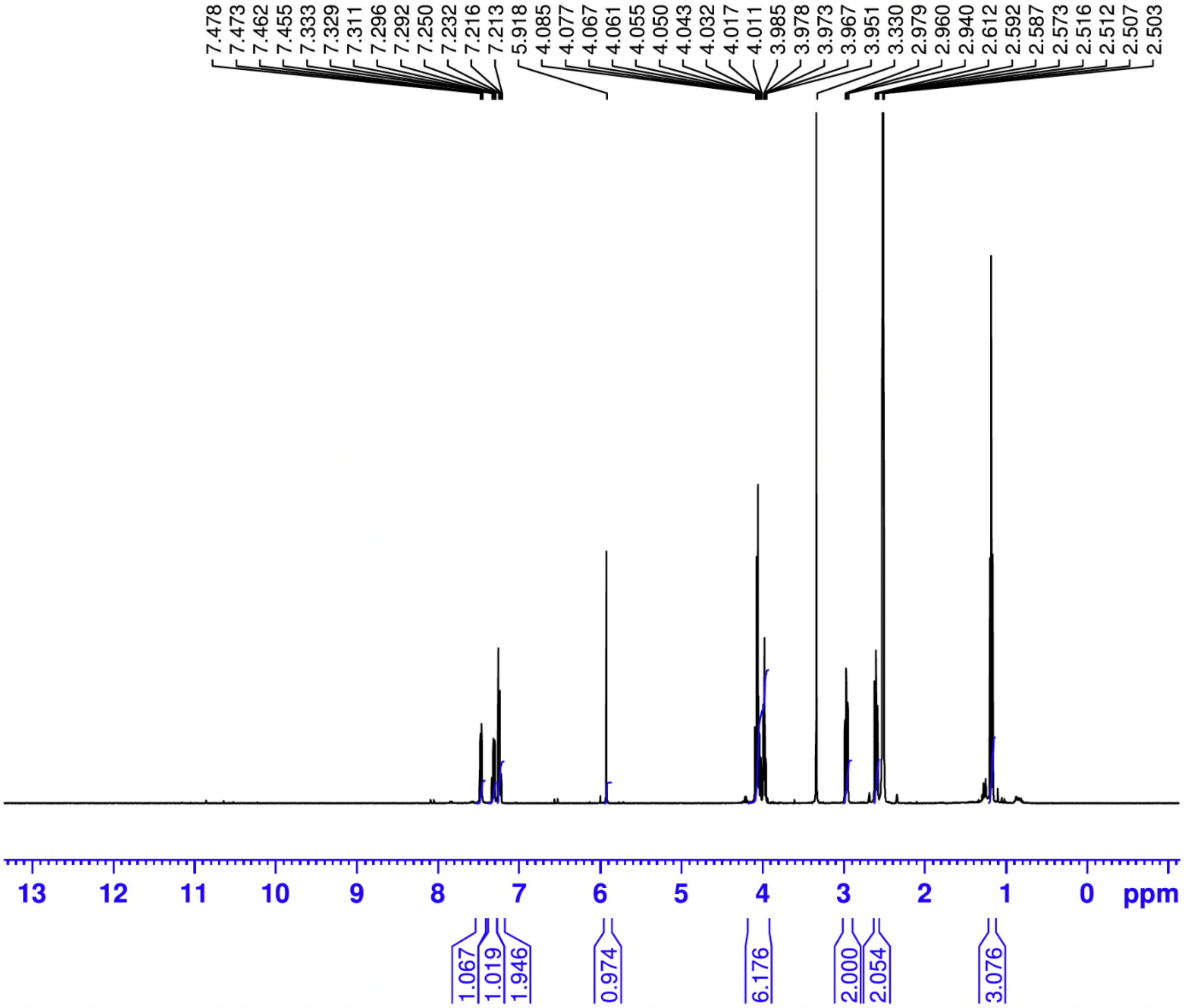

Supplementary Fig. 17 | NMR spectrum for compound 10

(11)

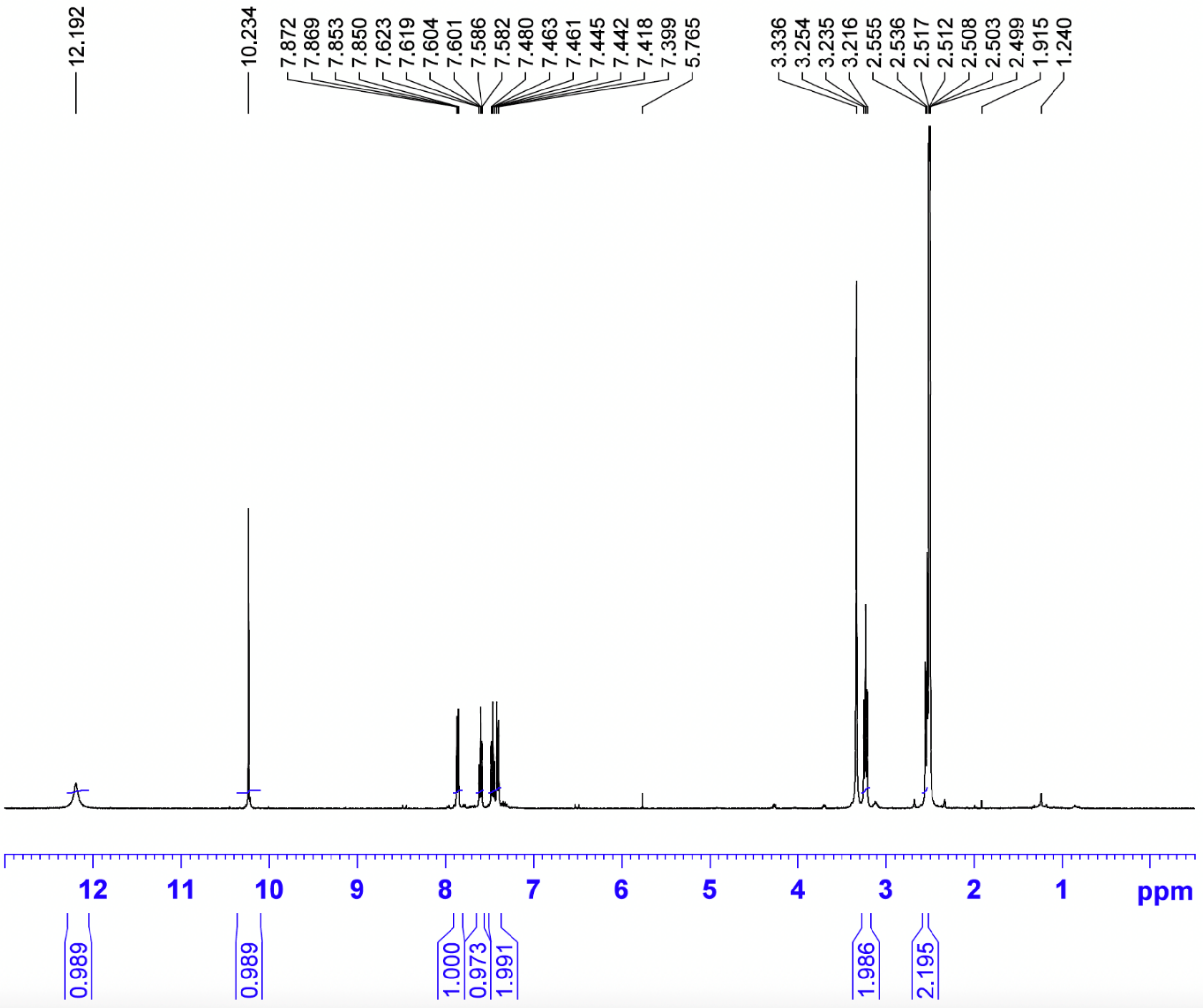

Supplementary Fig. 18 | NMR spectrum for compound 11

UNC10413729 (12)

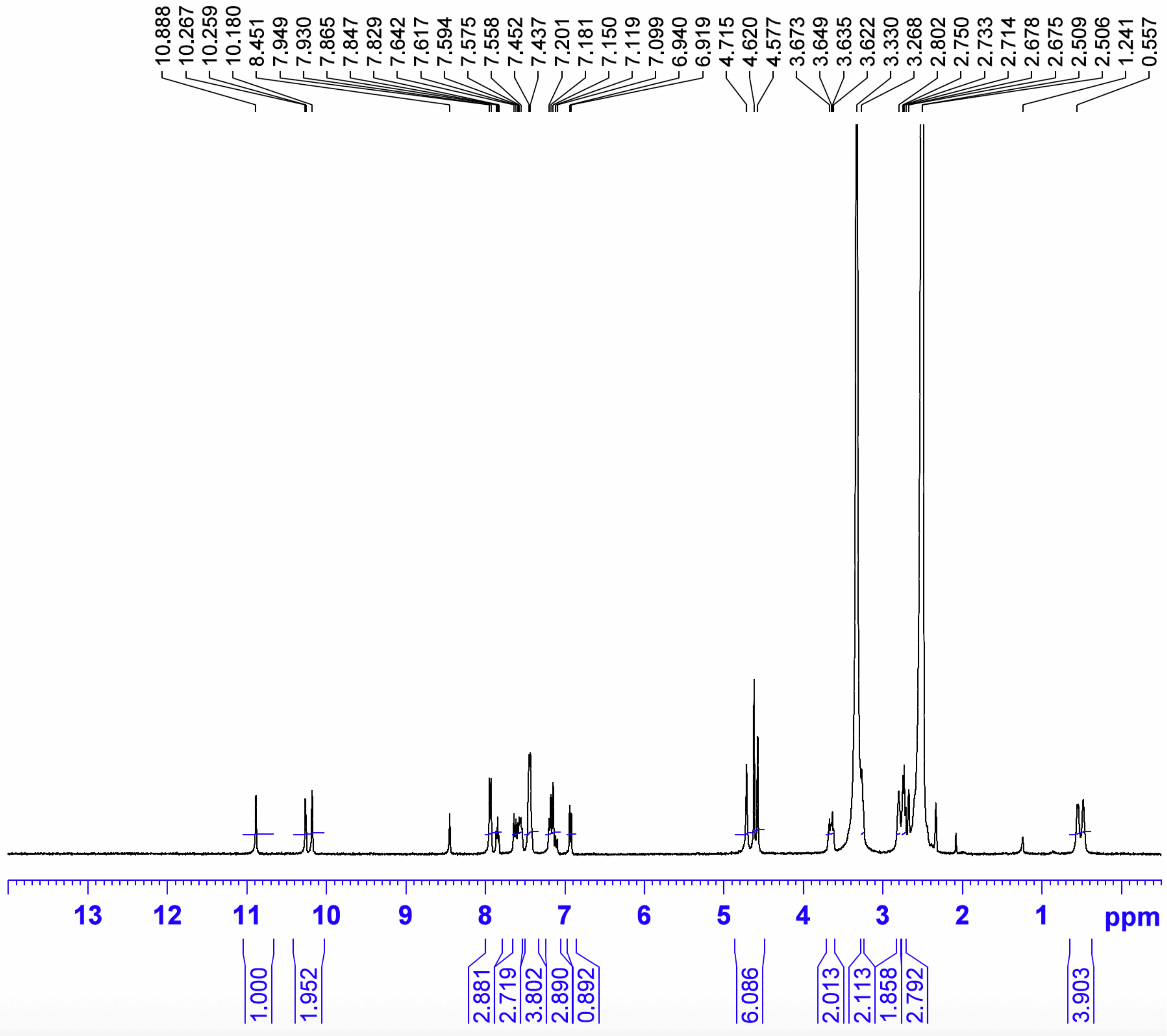

Supplementary Fig. 19 | NMR spectrum for compound 12

(14)

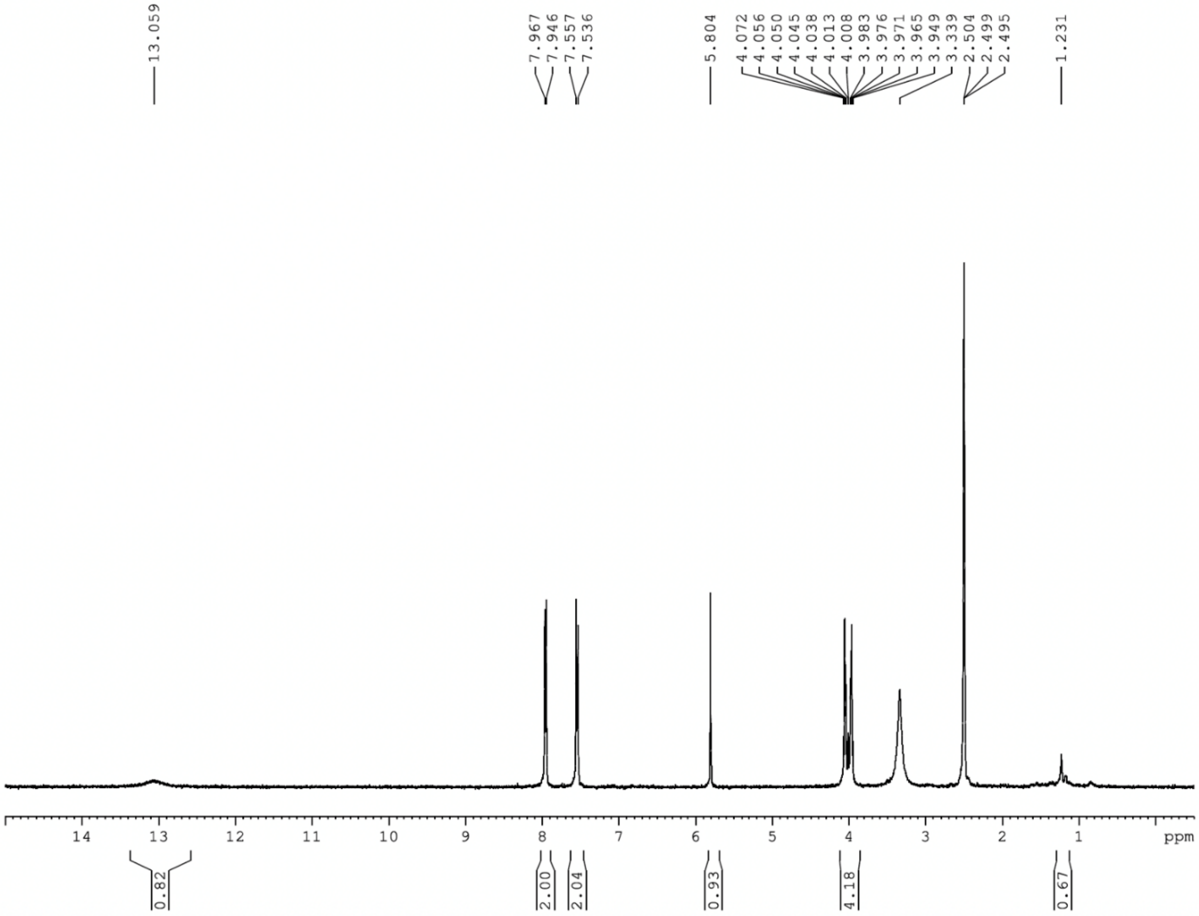

Supplementary Fig. 20 | NMR spectrum for compound 14

UNC10415667 (15)

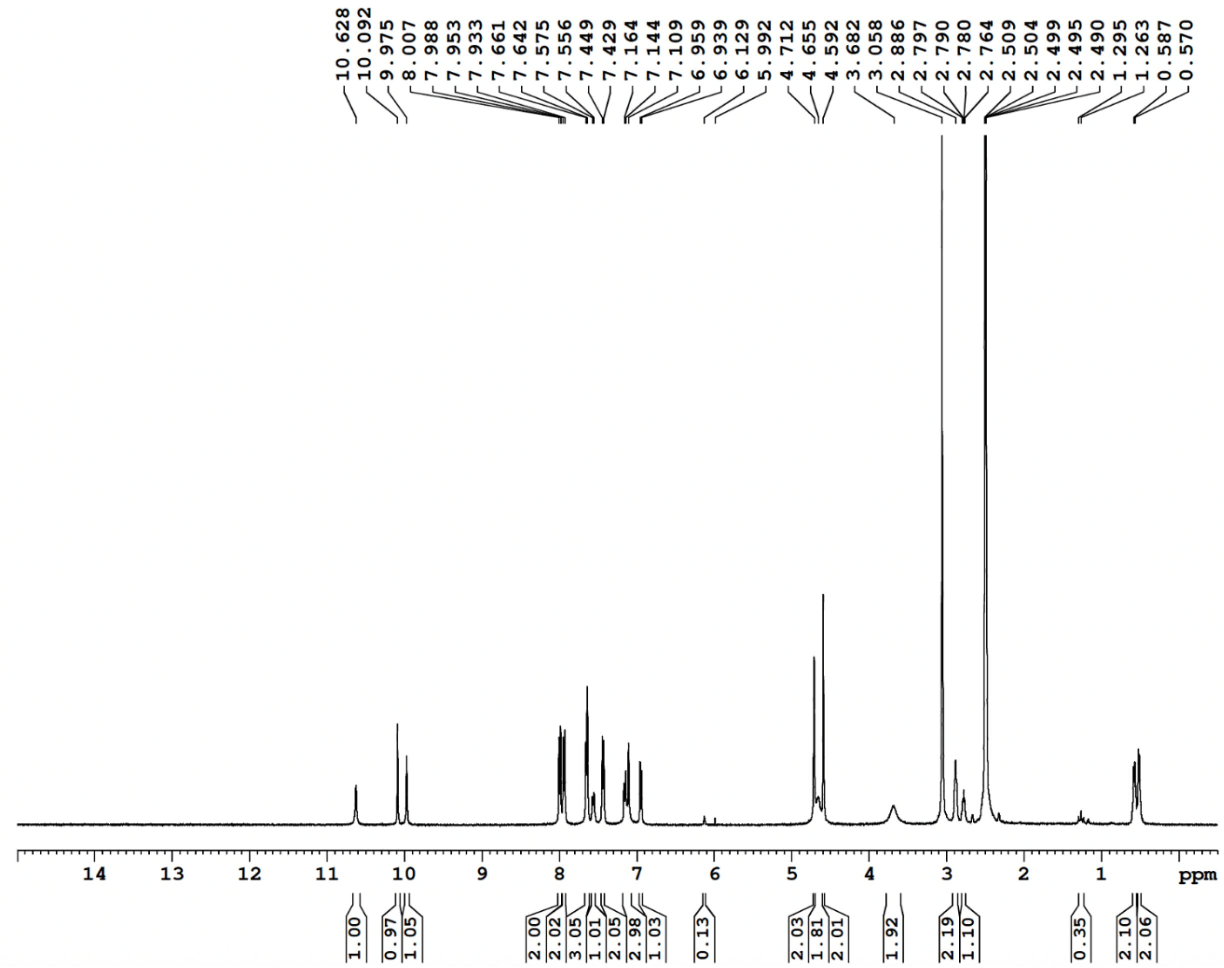

Supplementary Fig. 21 | NMR spectrum for compound 15

UNC10415668 (19)

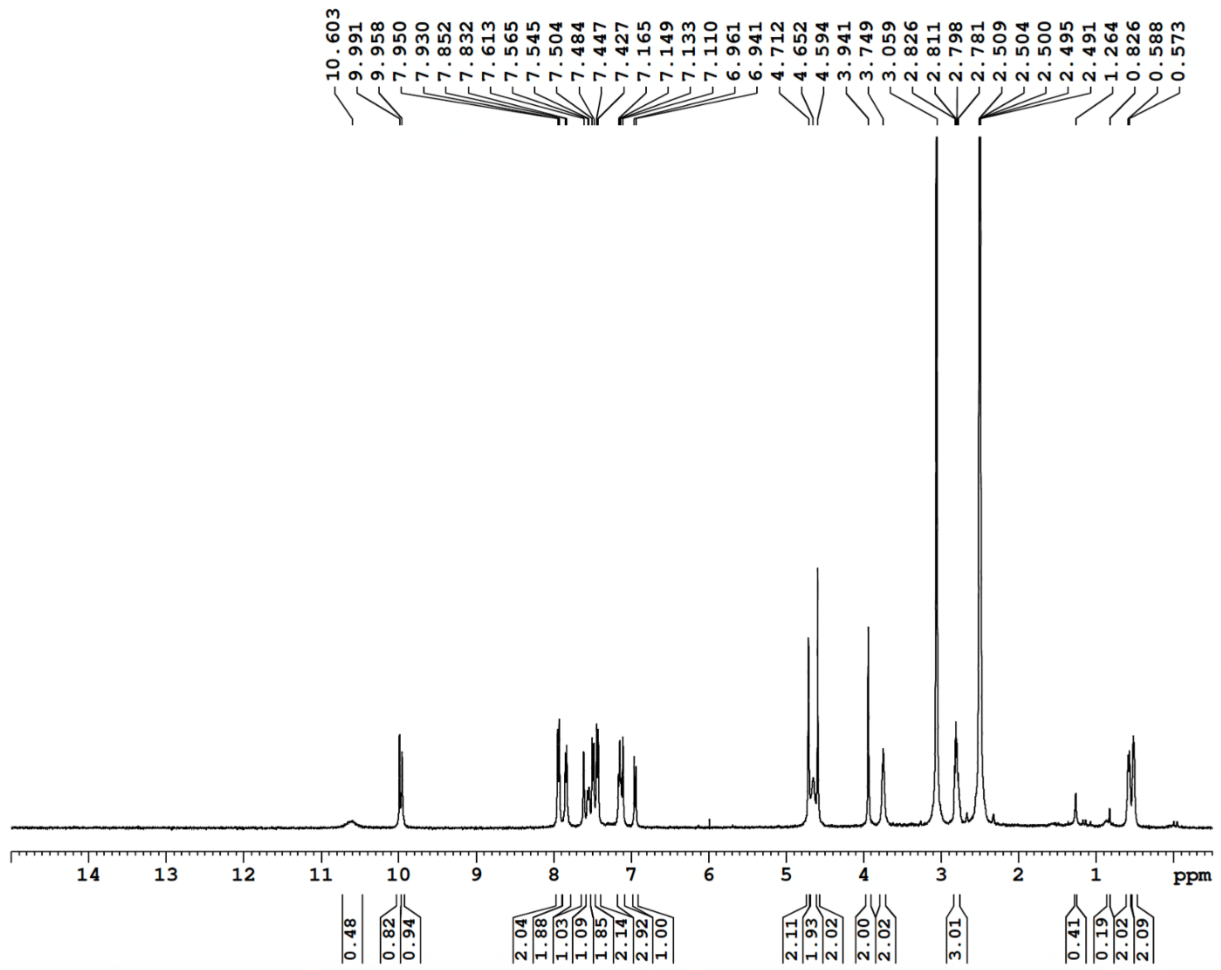

Supplementary Fig. 22 | NMR spectrum for compound 19

(21)

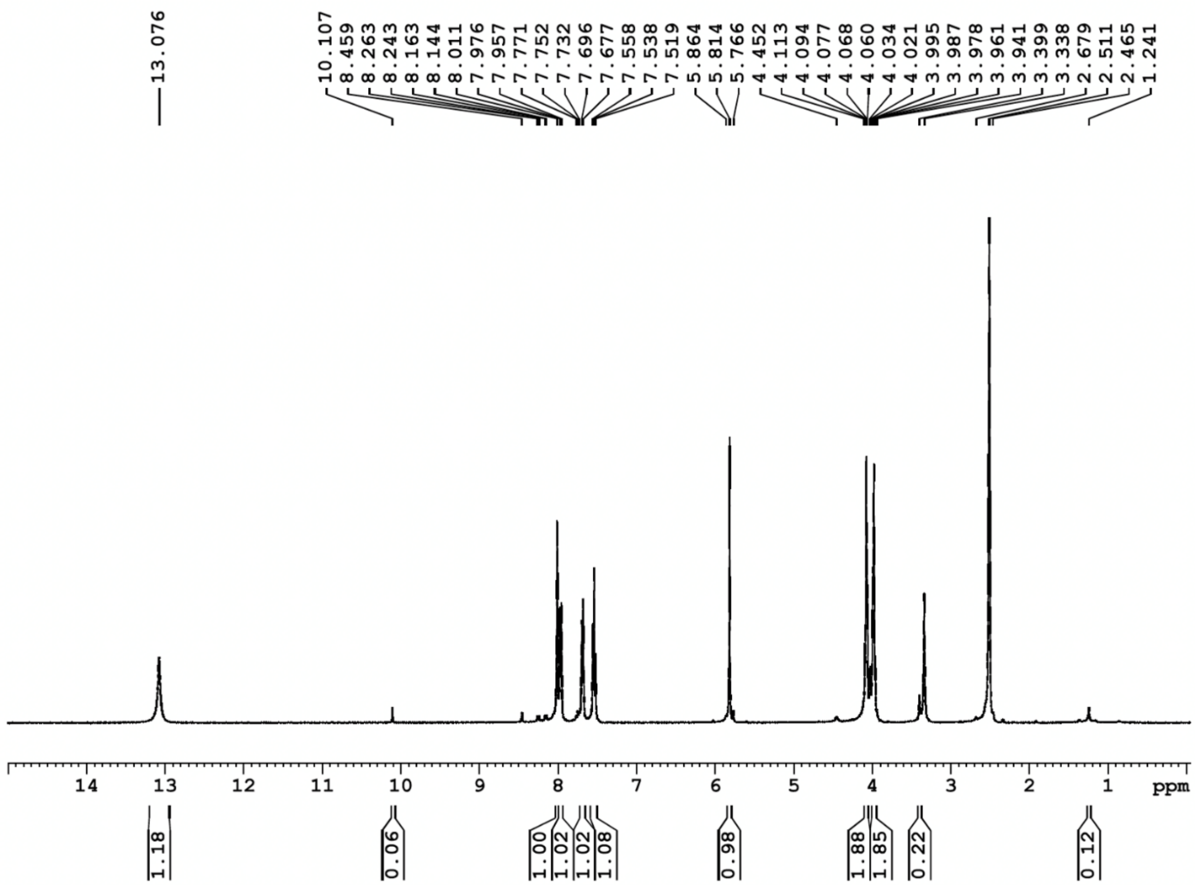

Supplementary Fig. 23 | NMR spectrum for compound 21

UNC10415669 (22)

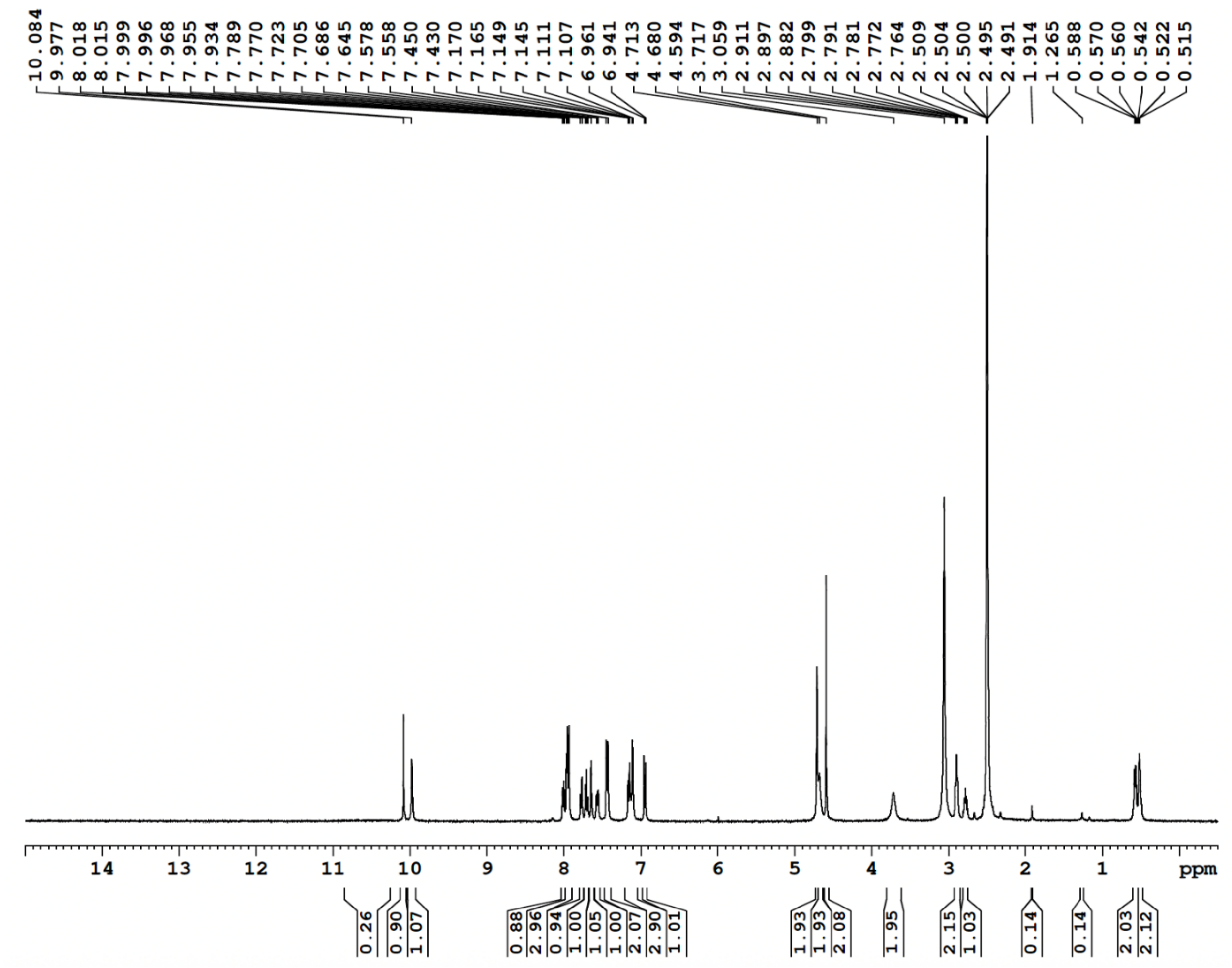

Supplementary Fig. 24 | NMR spectrum for compound 22

(23)

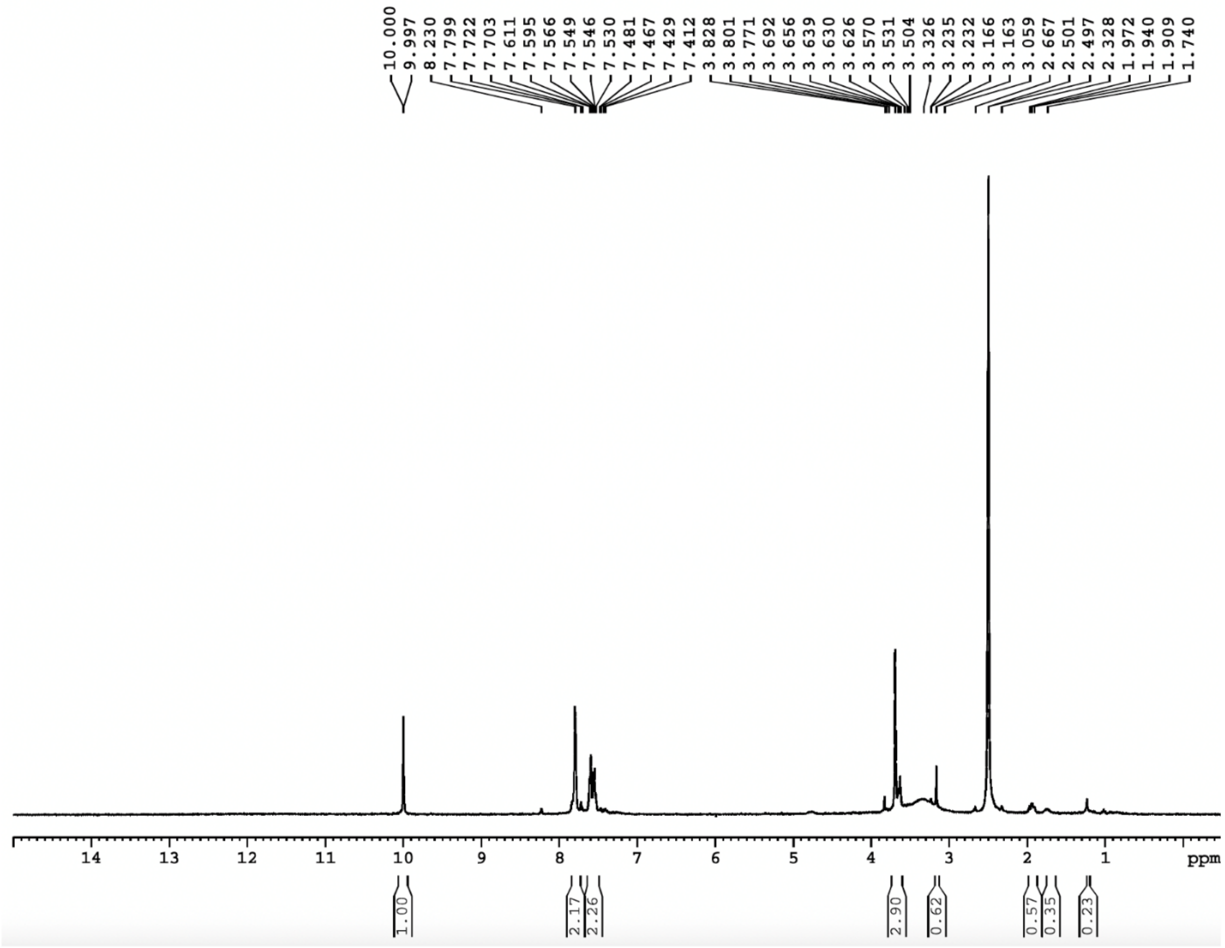

Supplementary Fig. 25 | NMR spectrum for compound 23

UNC10415670 (24)

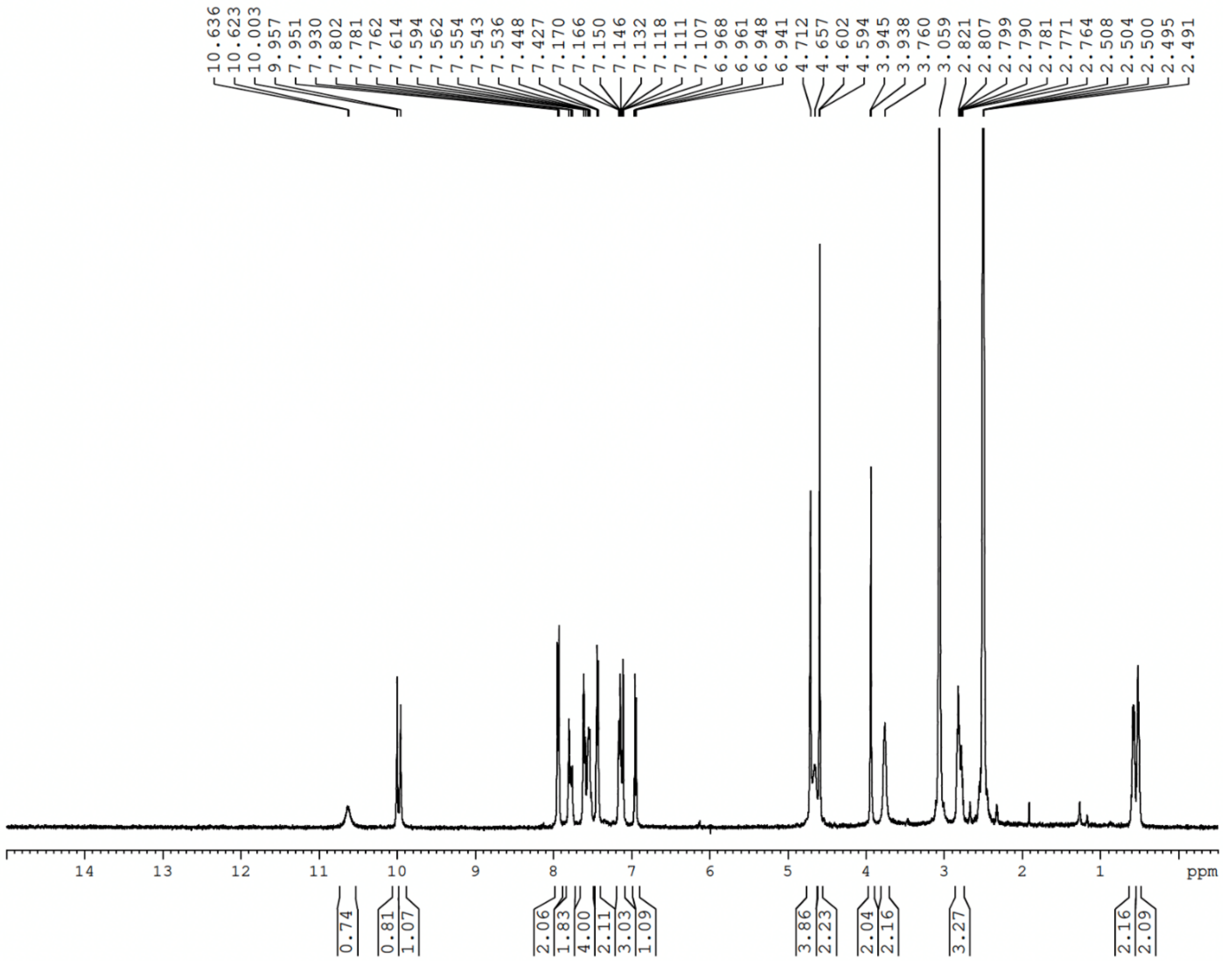

Supplementary Fig. 26 | NMR spectrum for compound 24

UNC10415671 (25)

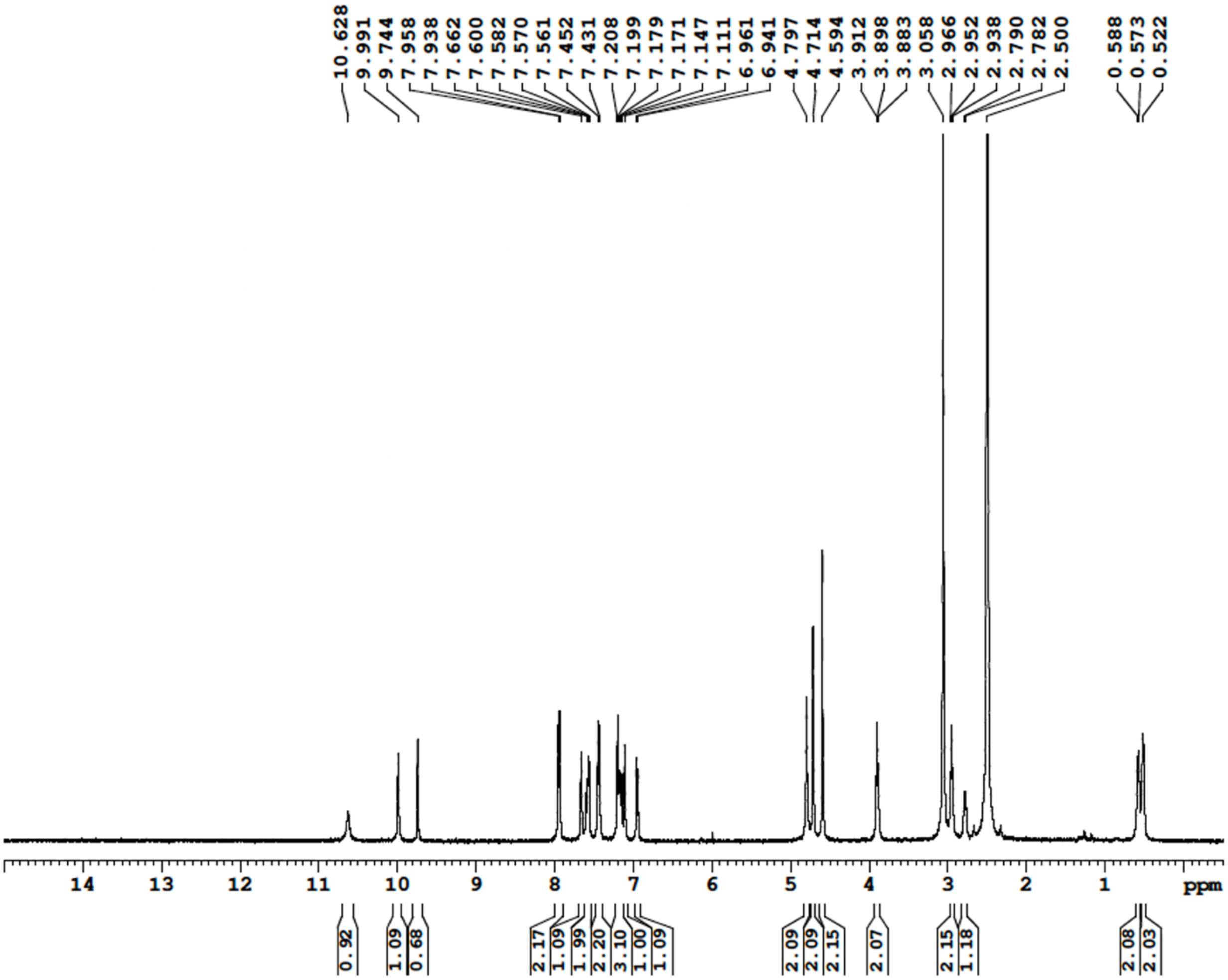

Supplementary Fig. 27 | NMR spectrum for compound 25

UNC12145 (26)

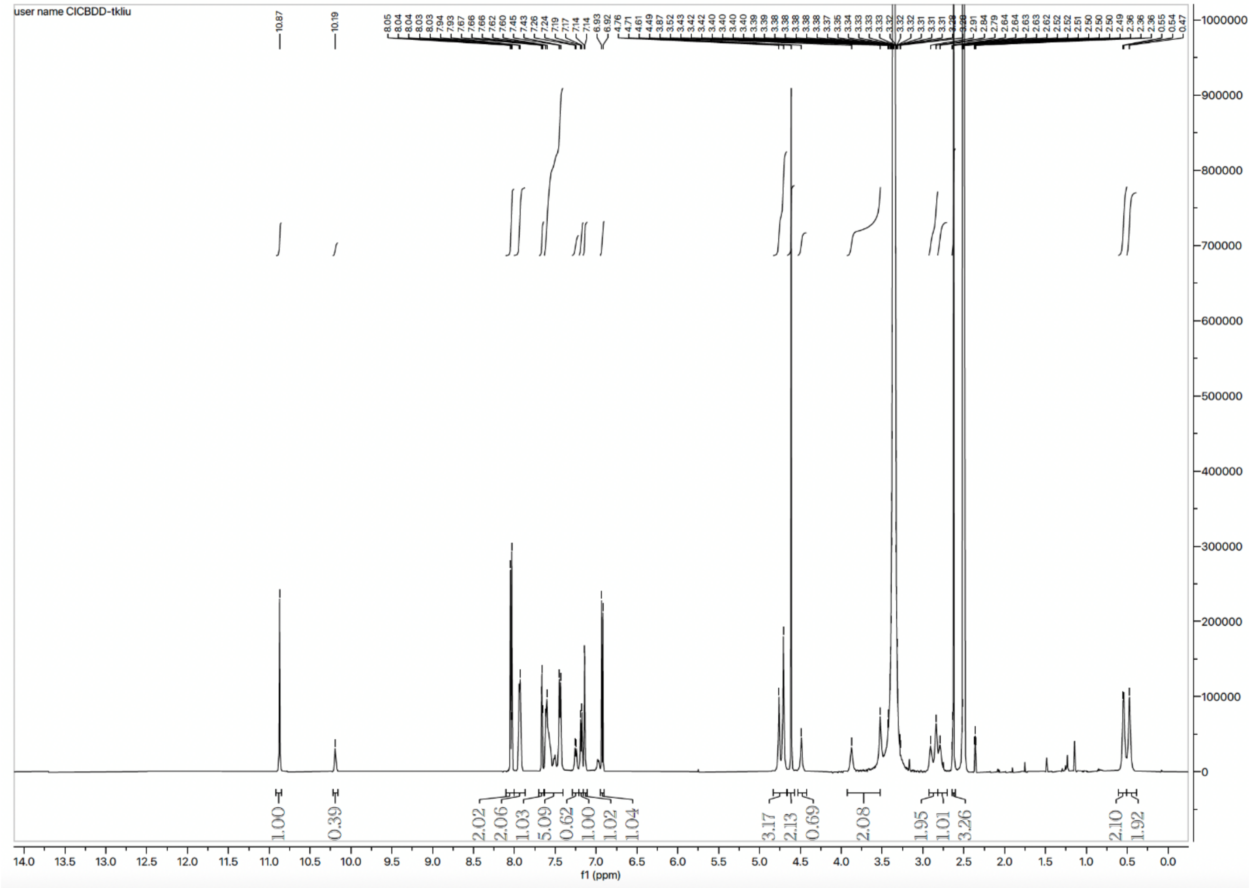

Supplementary Fig. 28 | NMR spectrum for compound 26

UNC12148 (29)

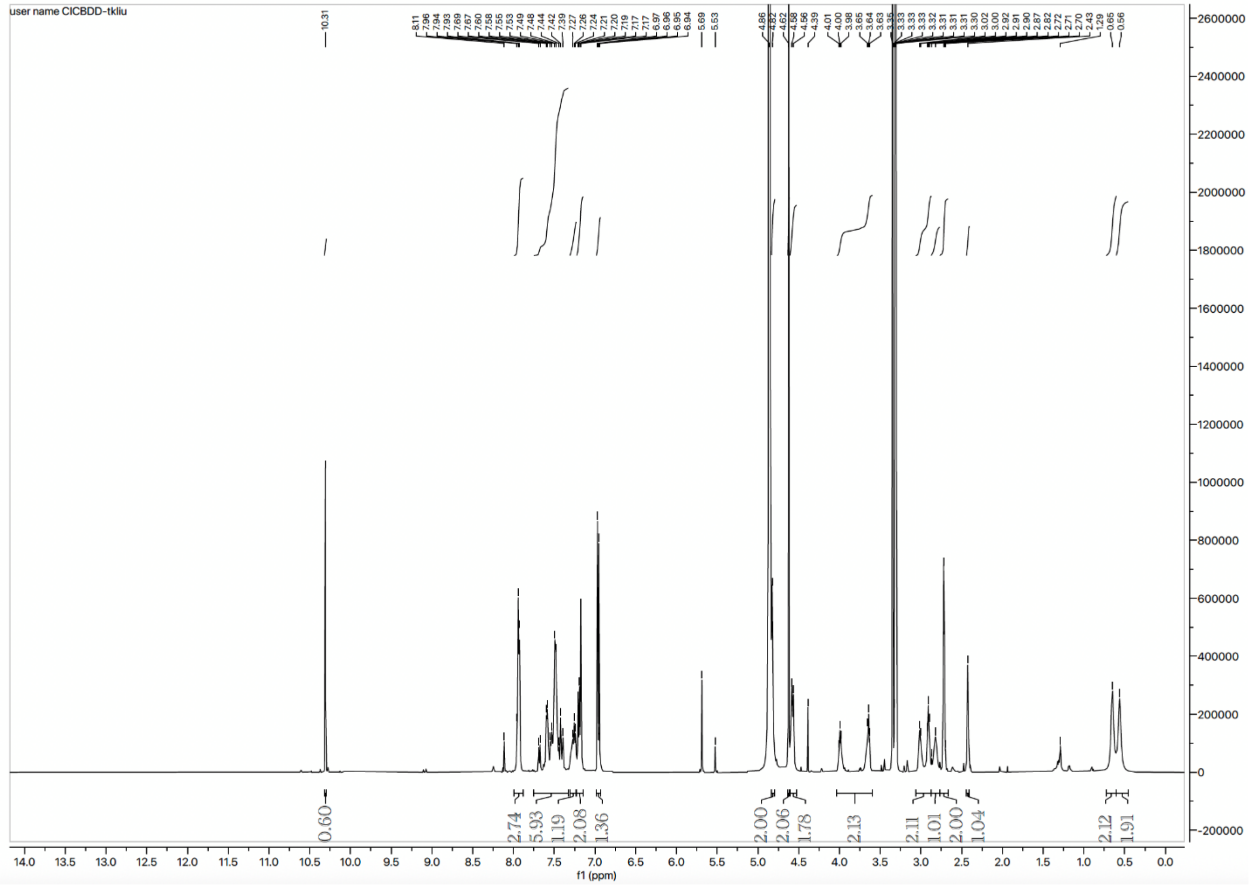

Supplementary Fig. 29 | NMR spectrum for compound 29

UNC12149 (32)

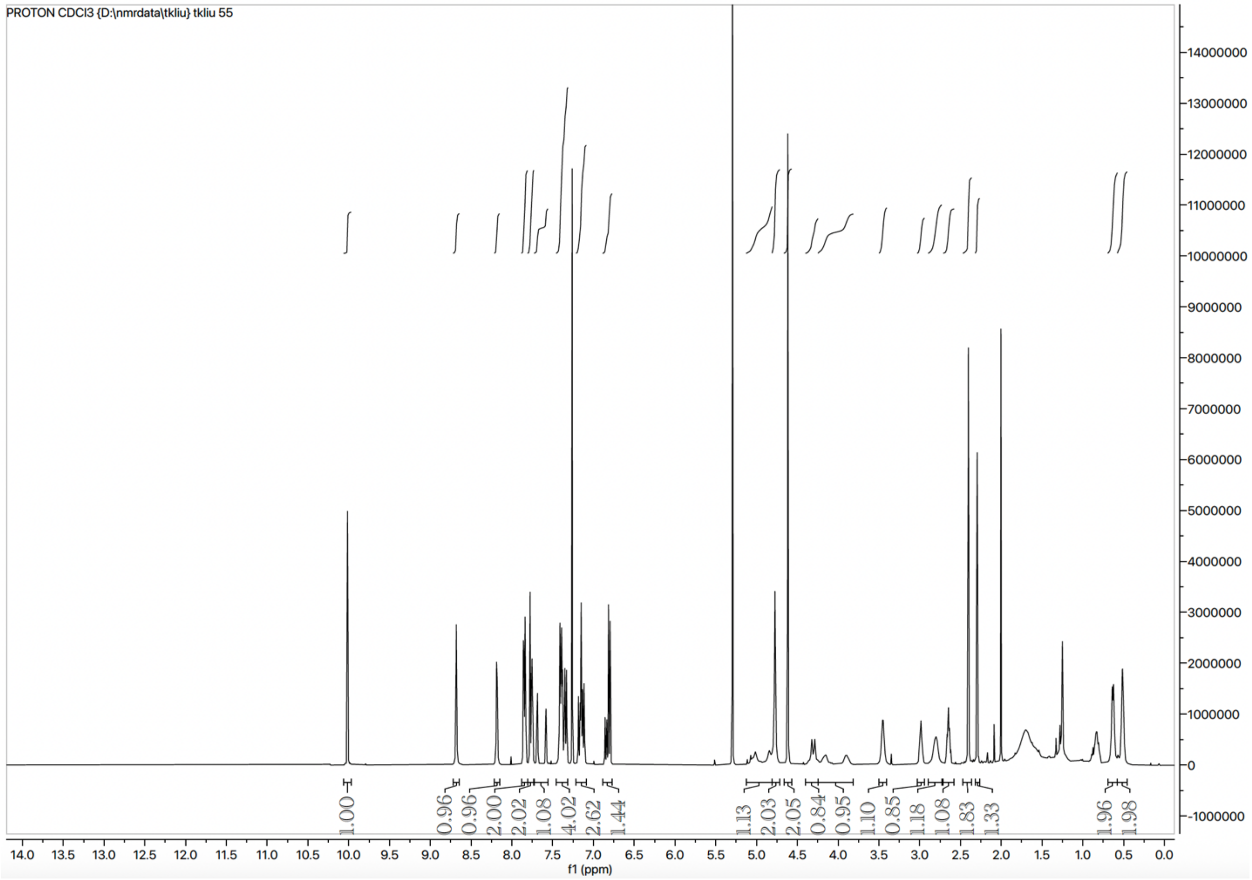

Supplementary Fig. 30 | NMR spectrum for compound 32

UNC12150 (35)

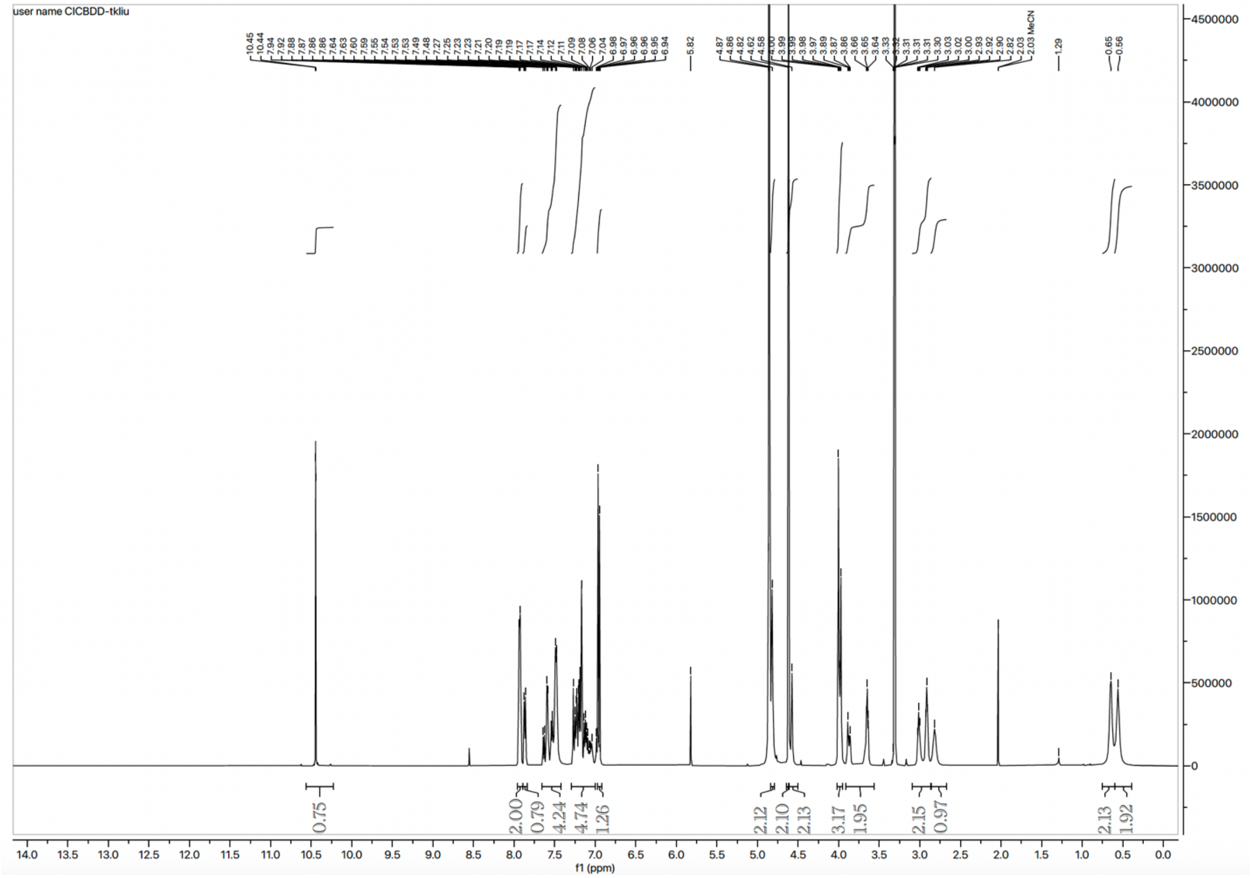

Supplementary Fig. 31 | NMR spectrum for compound 35

UNC12151 (36)

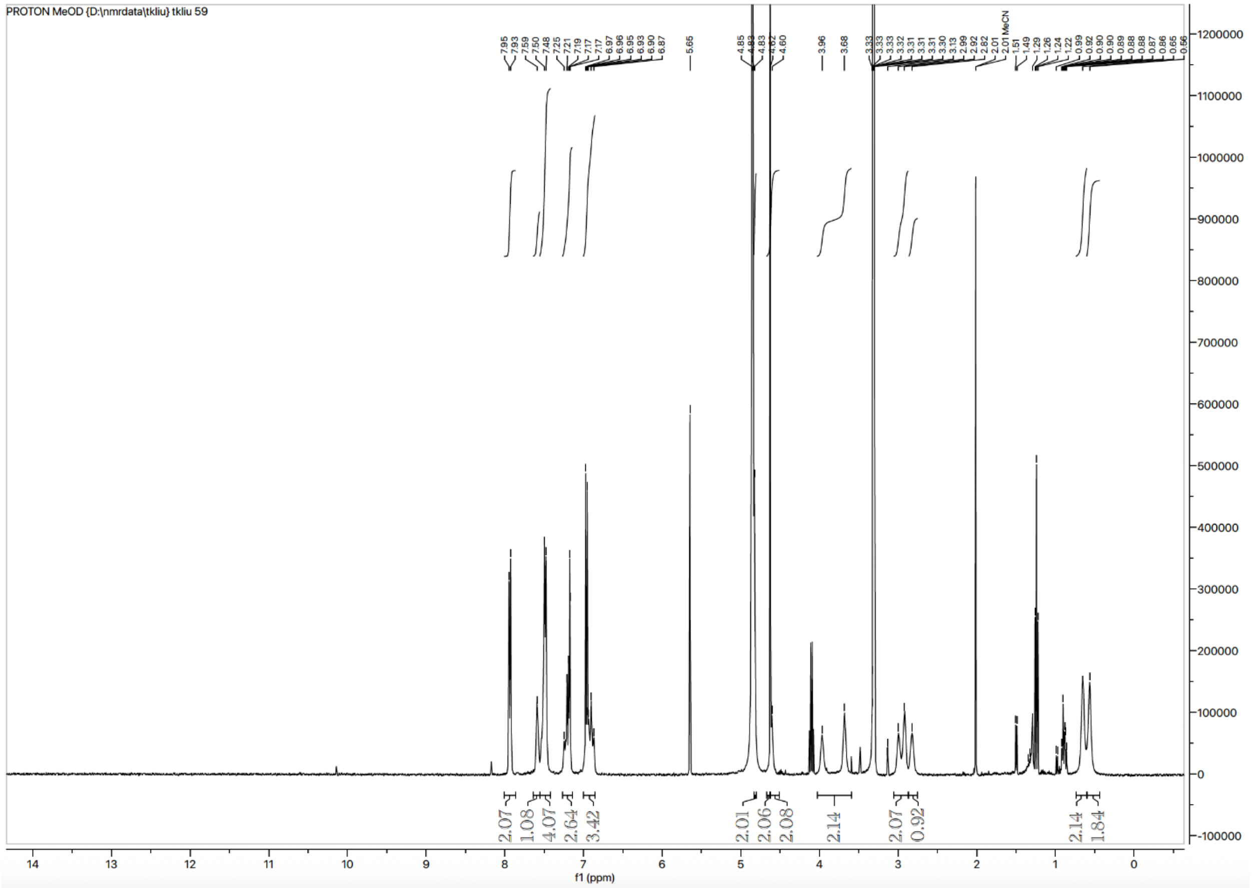

Supplementary Fig. 32 | NMR spectrum for compound 36

UNC12567 (38)

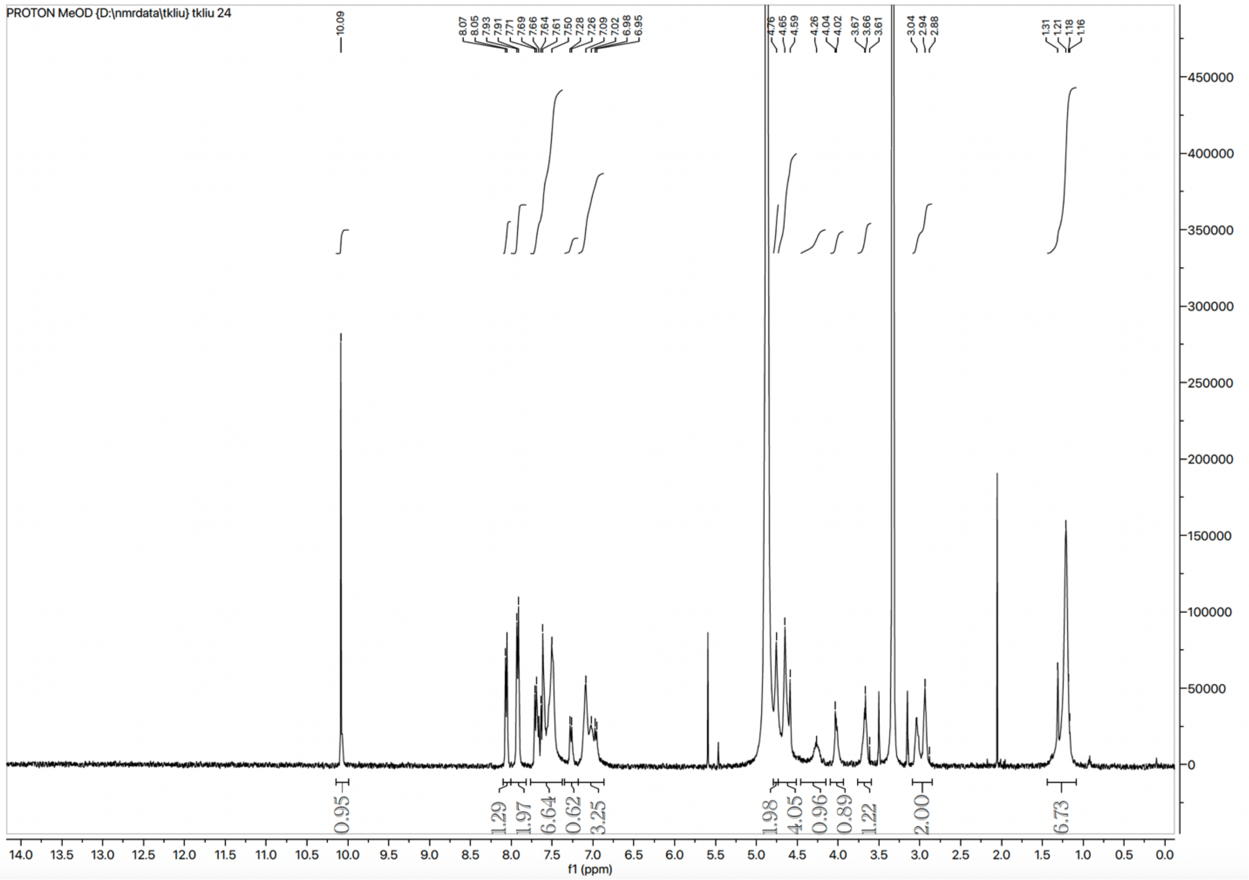

Supplementary Fig. 33 | NMR spectrum for compound 38

LCMS Spectra

(2)

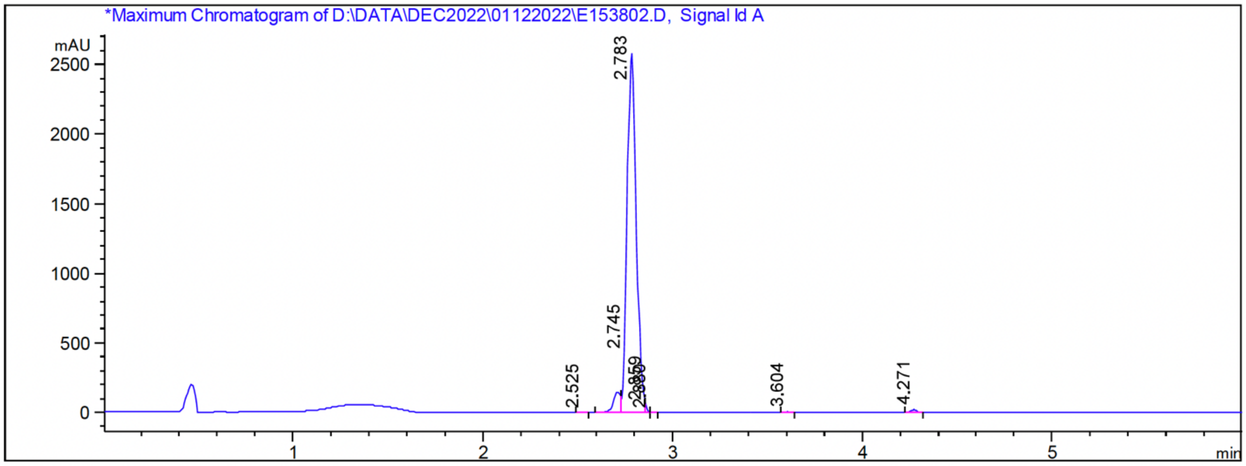

Supplementary Fig. 34 | LCMS spectrum for compound 2

(3)

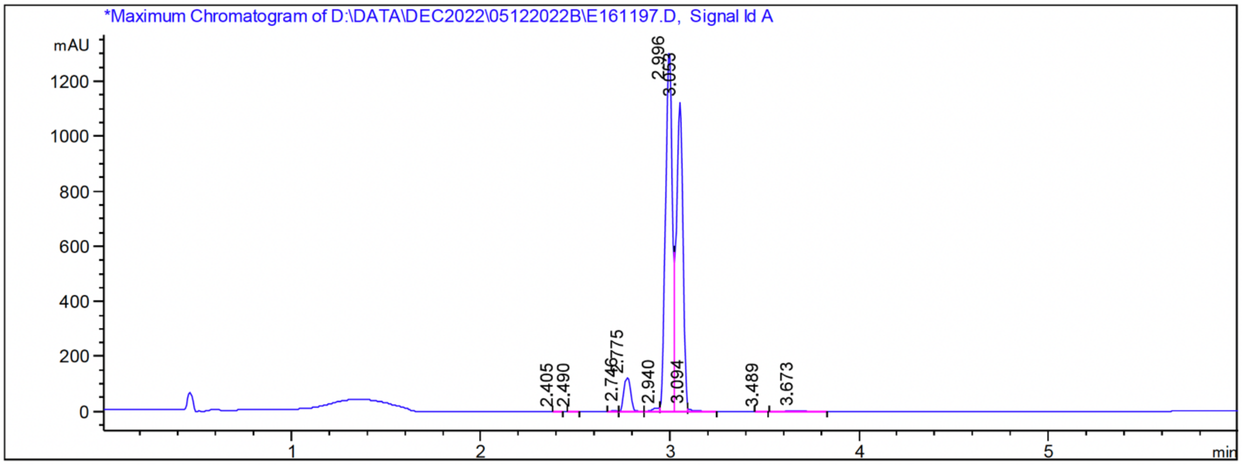

Supplementary Fig. 35 | LCMS spectrum for compound 3

(4)

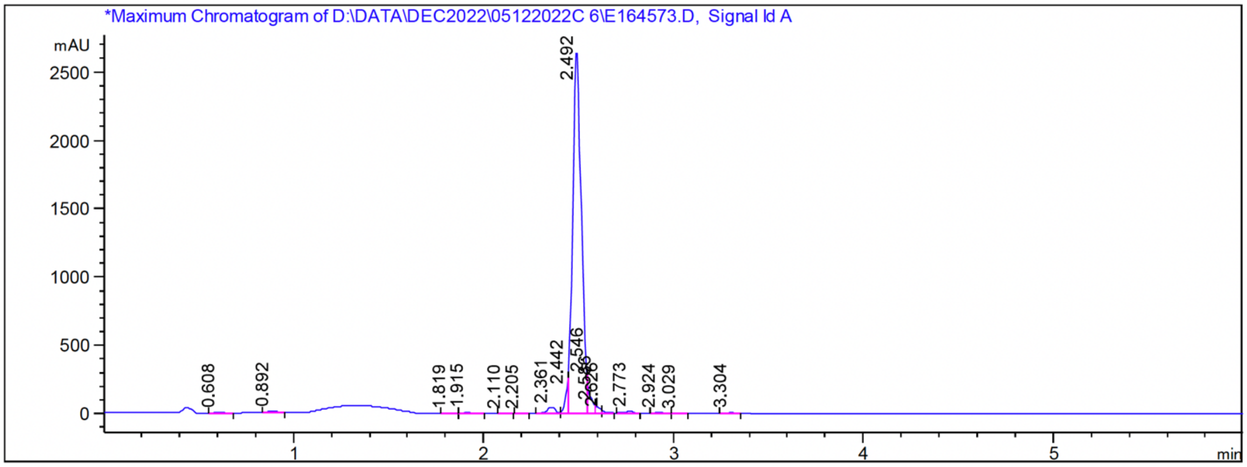

Supplementary Fig. 36 | LCMS spectrum for compound 4

UNC10413724 (5)

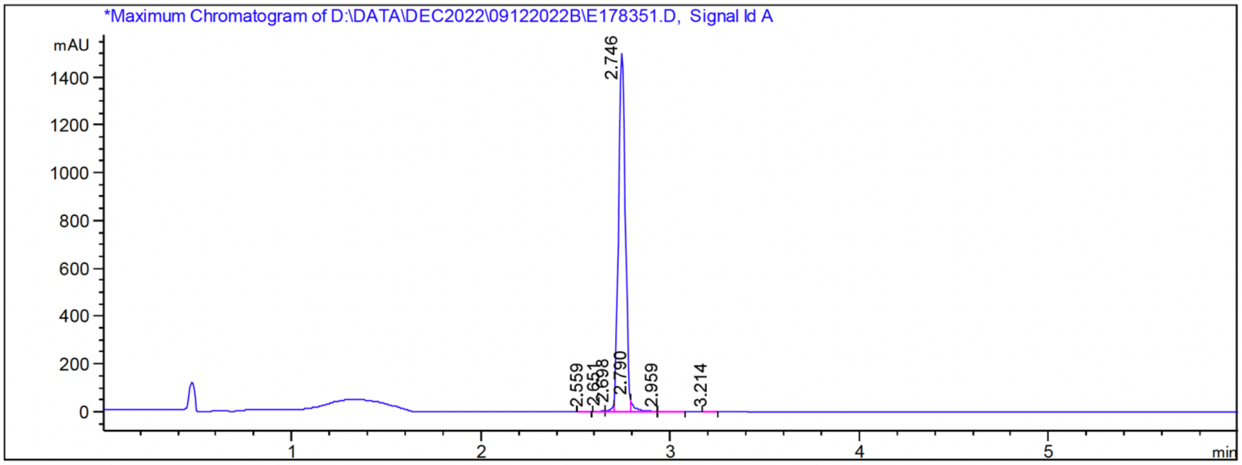

Supplementary Fig. 37 | LCMS spectrum for compound 5

(6)

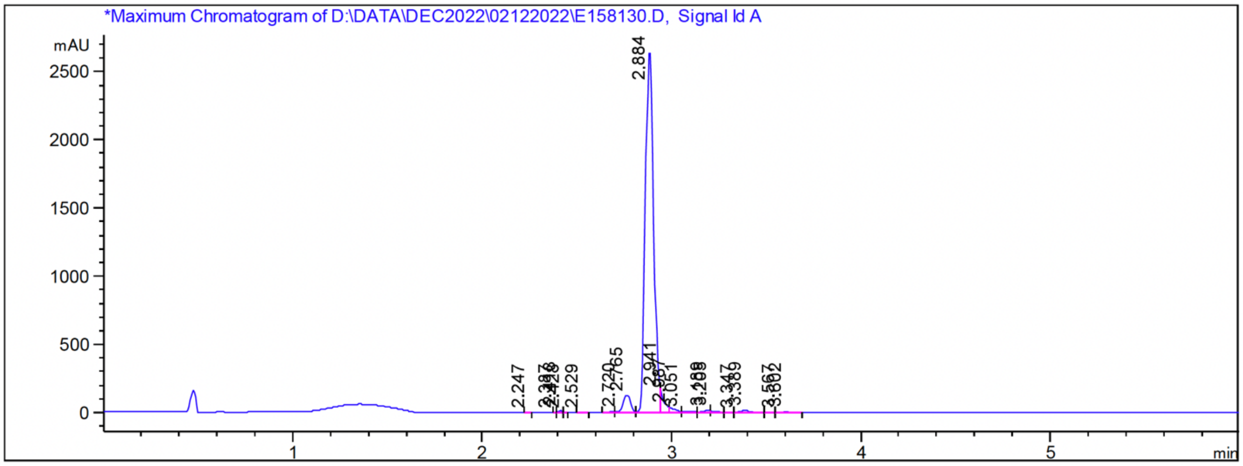

Supplementary Fig. 38 | LCMS spectrum for compound 6

(7)

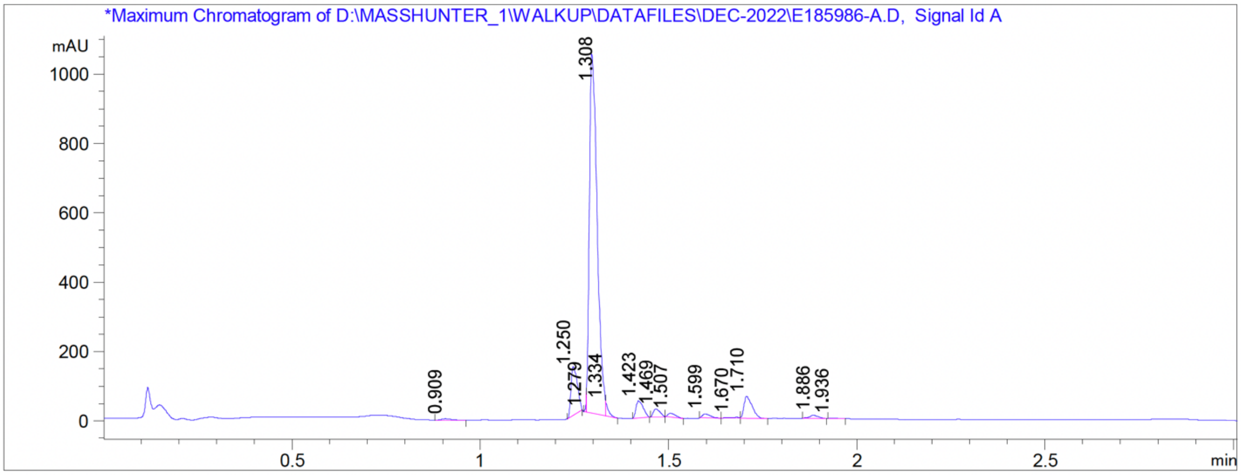

Supplementary Fig. 39 | LCMS spectrum for compound 7

UNC10413728 (8)

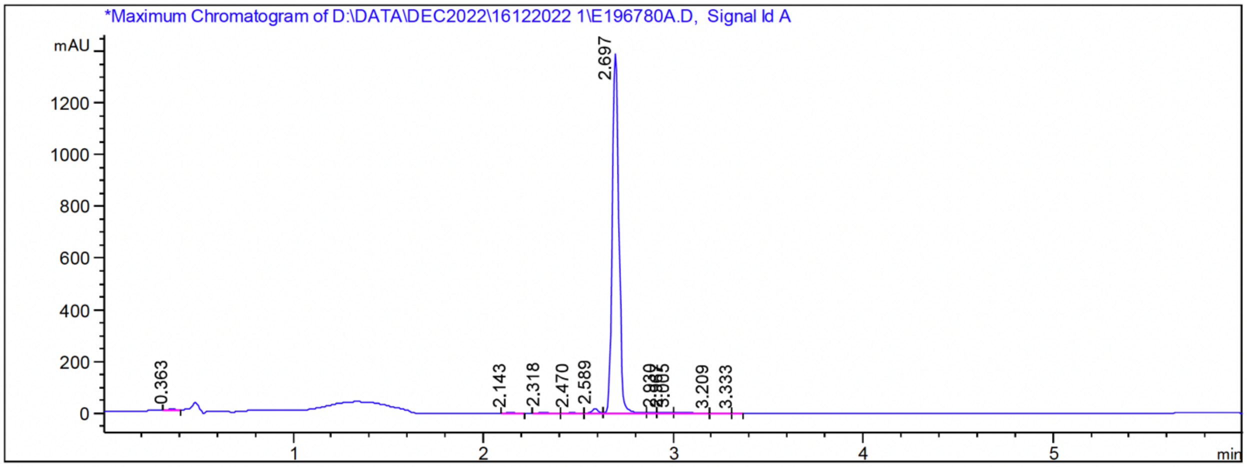

Supplementary Fig. 40 | LCMS spectrum for compound 8

(9)

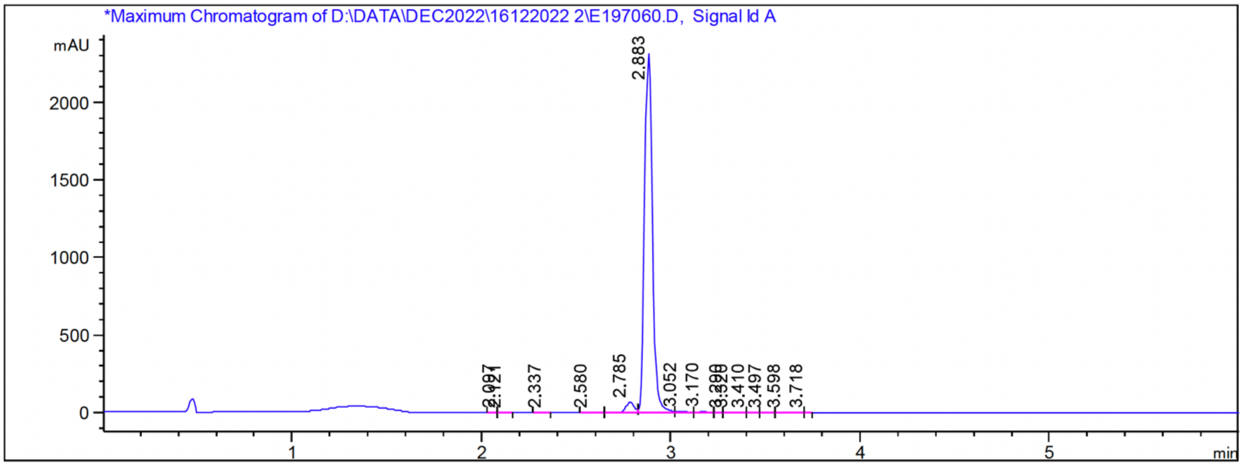

Supplementary Fig. 41 | LCMS spectrum for compound 9

(10)

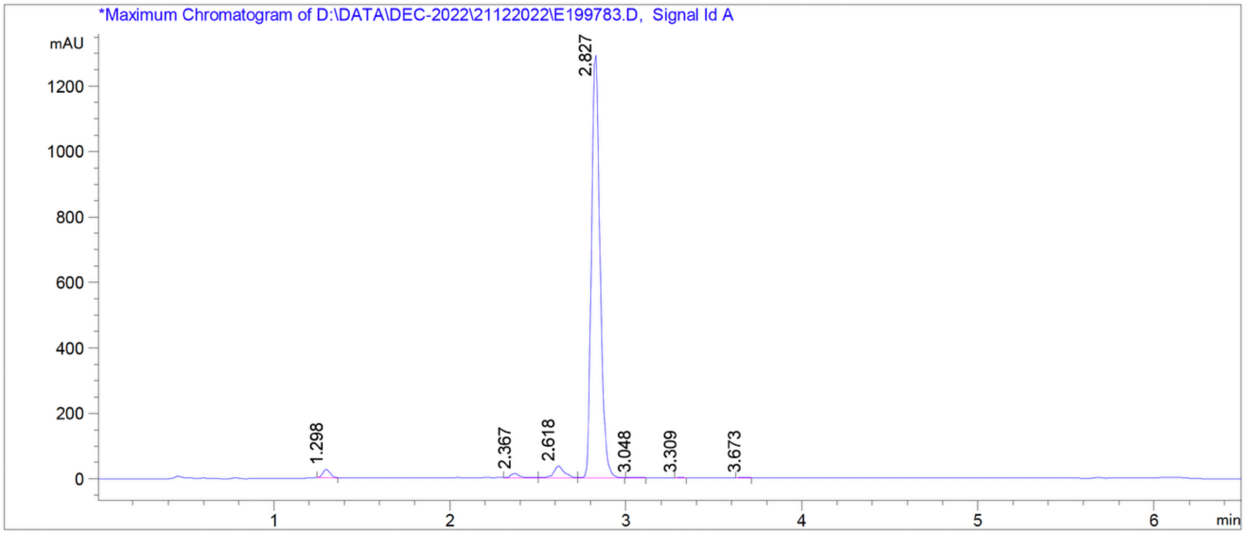

Supplementary Fig. 42 | LCMS spectrum for compound 10

(11)

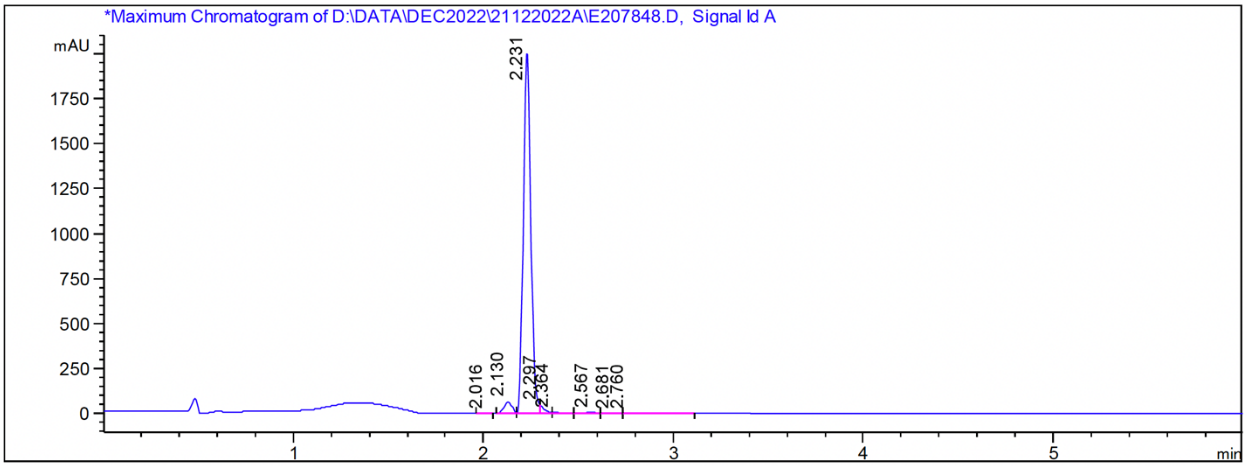

Supplementary Fig. 43 | LCMS spectrum for compound 11

UNC10413729 (12)

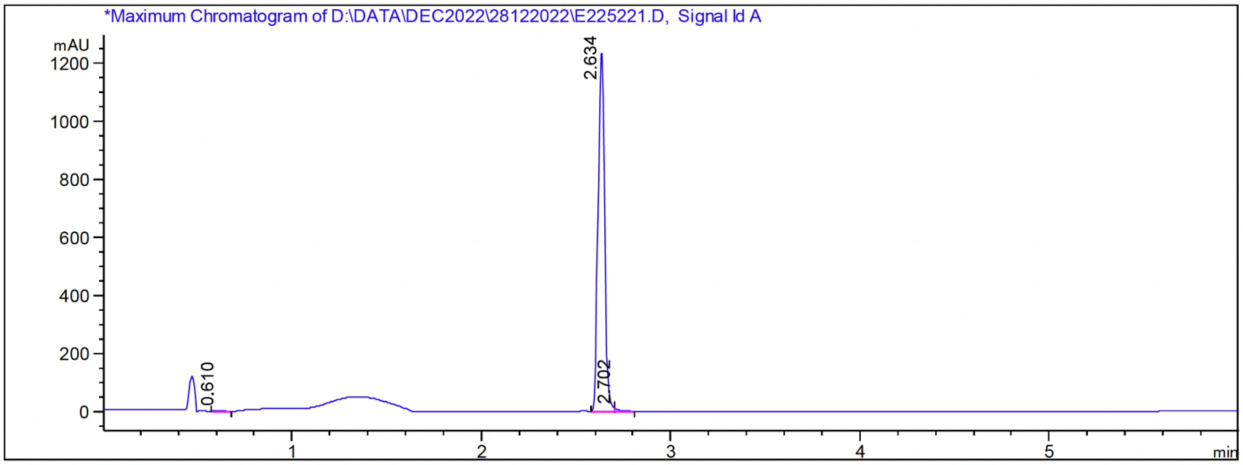

Supplementary Fig. 44 | LCMS spectrum for compound 12

(14)

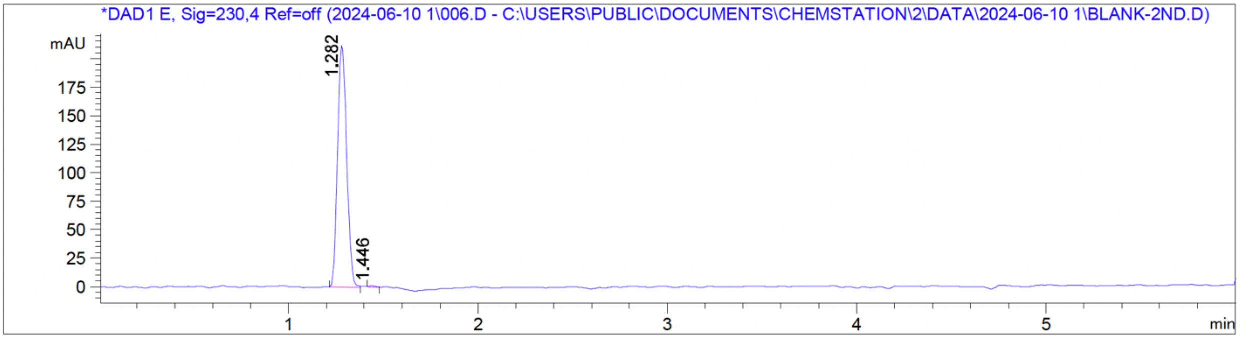

Supplementary Fig. 45 | LCMS spectrum for compound 14

UNC10415667 (15)

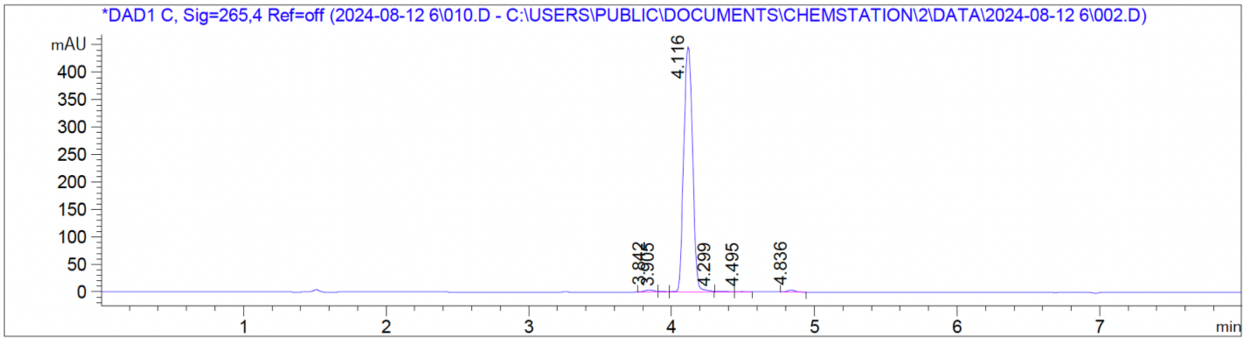

Supplementary Fig. 46 | LCMS spectrum for compound 15

(18)

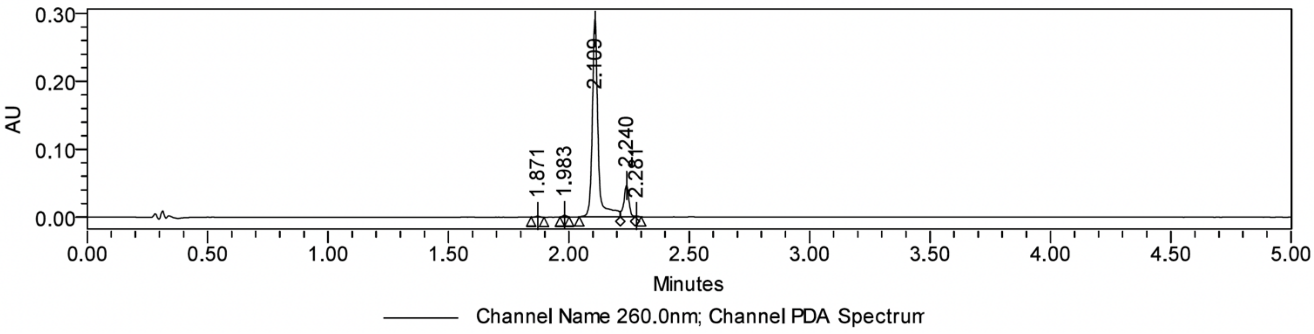

Supplementary Fig. 47 | LCMS spectrum for compound 18

UNC10415668 (19)

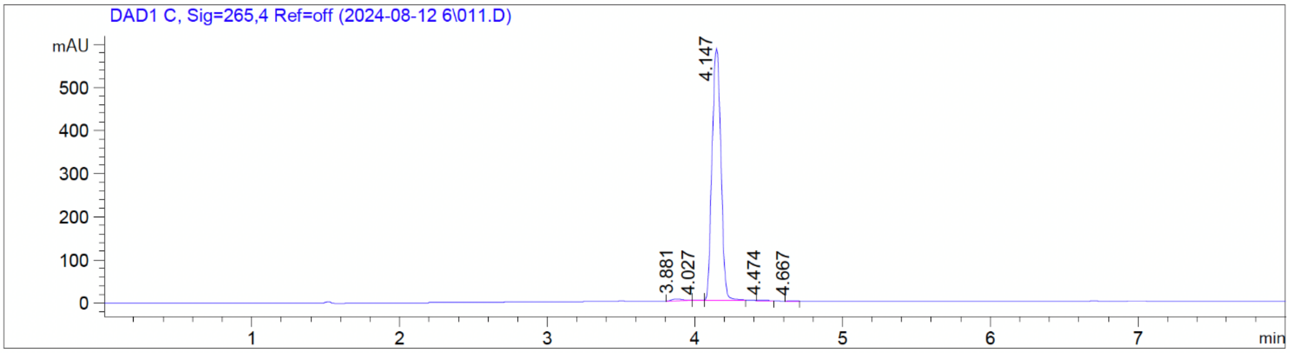

Supplementary Fig. 48 | LCMS spectrum for compound 19

(21)

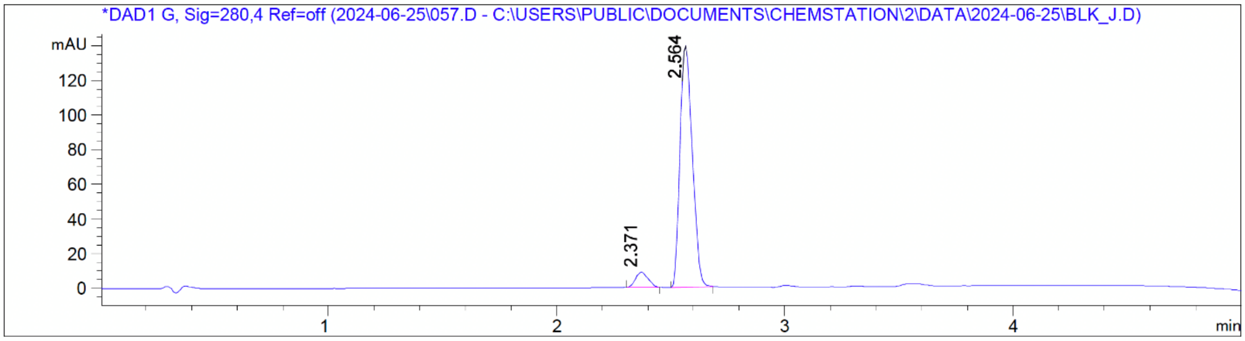

Supplementary Fig. 49 | LCMS spectrum for compound 21

UNC10415669 (22)

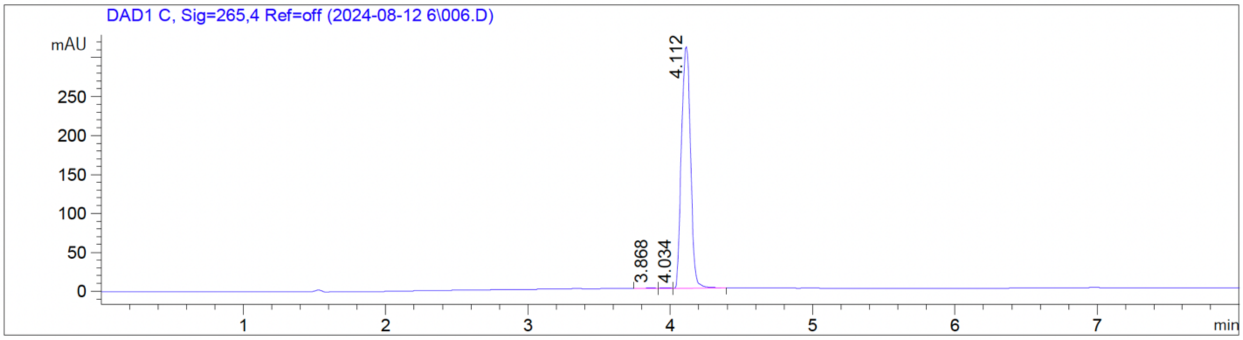

Supplementary Fig. 50 | LCMS spectrum for compound 22

UNC10415670 (24)

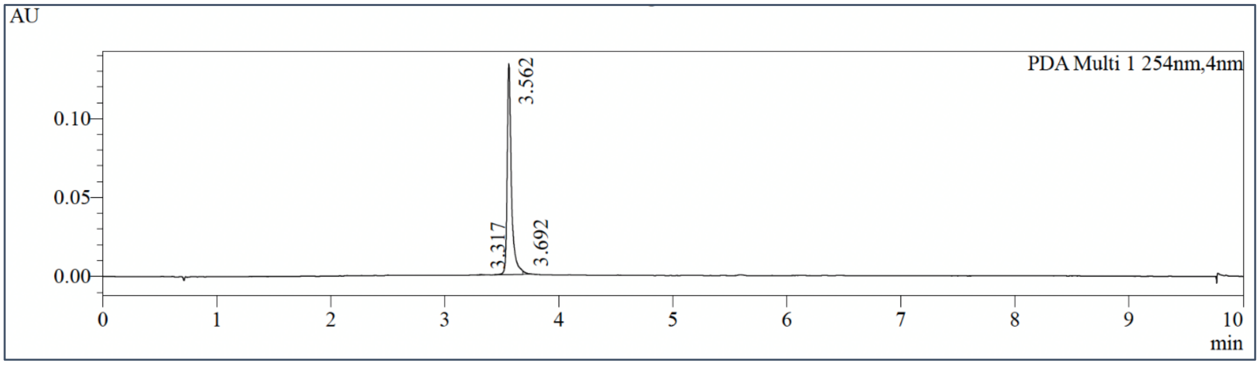

Supplementary Fig. 51 | LCMS spectrum for compound 24

UNC10415671 (25)

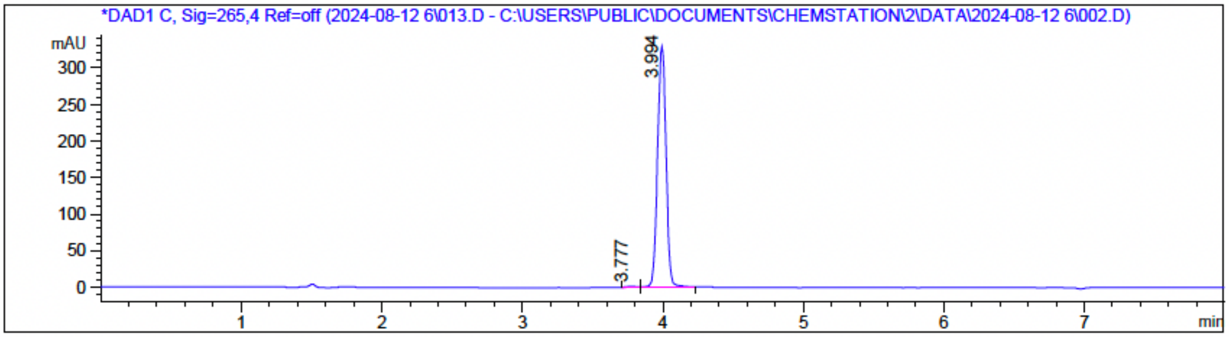

Supplementary Fig. 52 | LCMS spectrum for compound 25

UNC12145 (26)

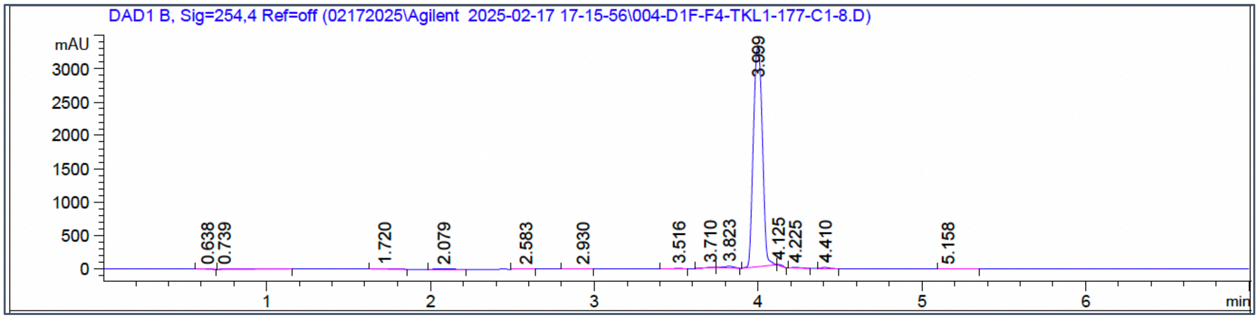

Supplementary Fig. 53 | LCMS spectrum for compound 26

(28)

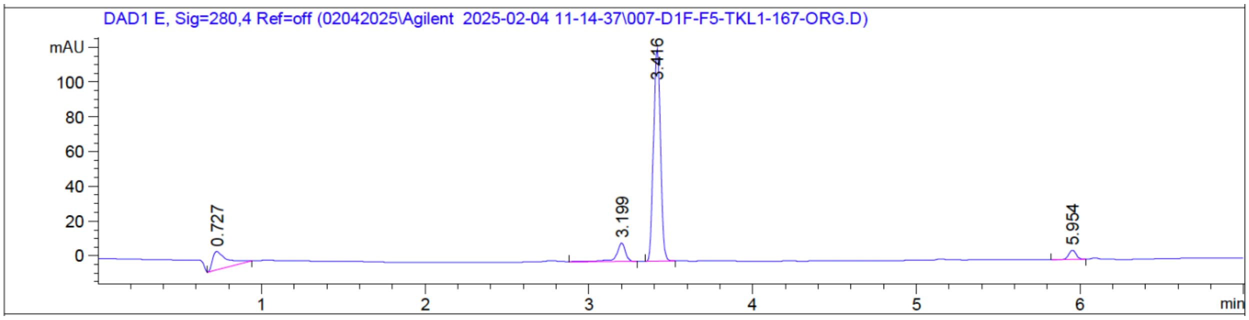

Supplementary Fig. 54 | LCMS spectrum for compound 28

UNC12148 (29)

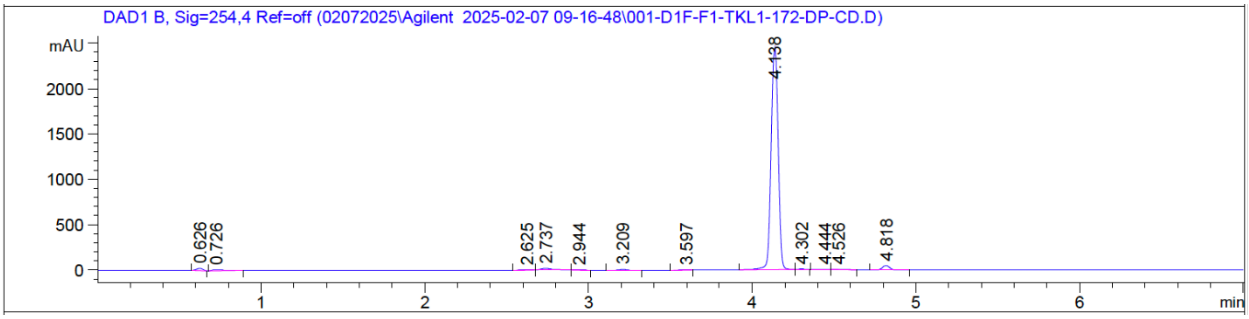

Supplementary Fig. 55 | LCMS spectrum for compound 29

(31)

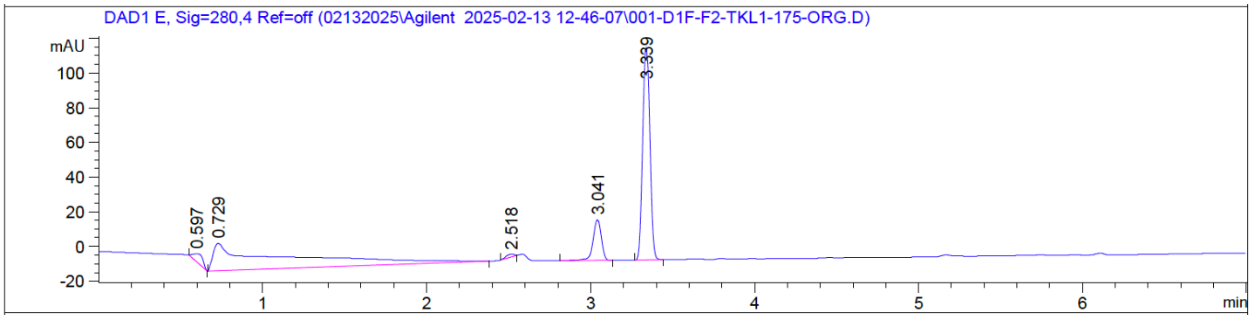

Supplementary Fig. 56 | LCMS spectrum for compound 31

UNC12149 (32)

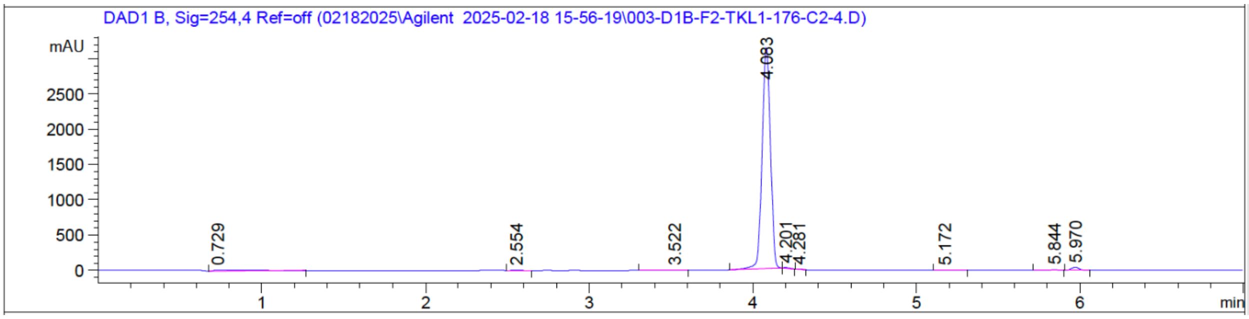

Supplementary Fig. 57 | LCMS spectrum for compound 32

(34)

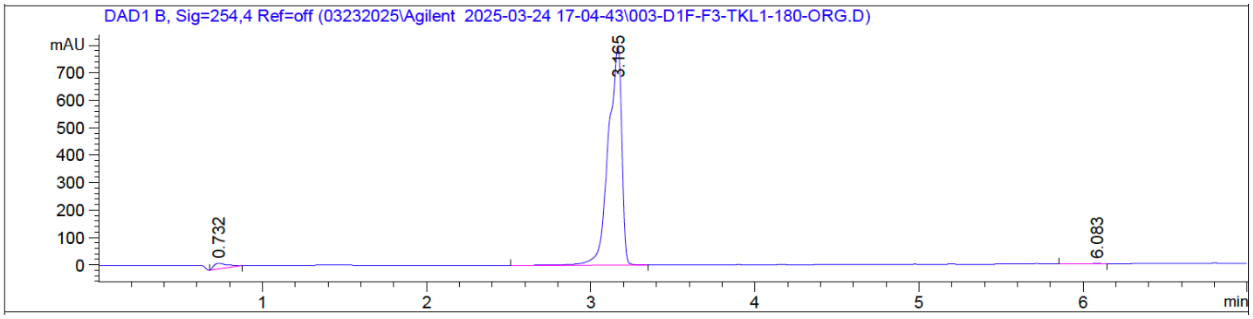

Supplementary Fig. 58 | LCMS spectrum for compound 34

UNC12150 (35)

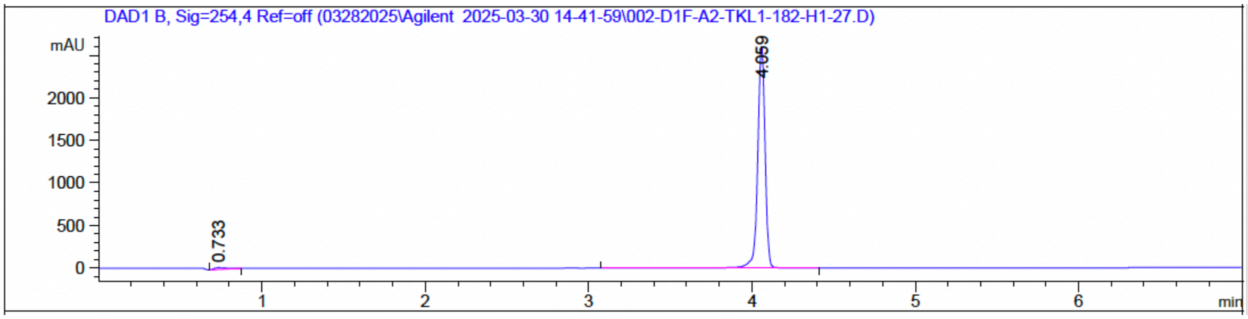

Supplementary Fig. 59 | LCMS spectrum for compound 35

UNC12151 (36)

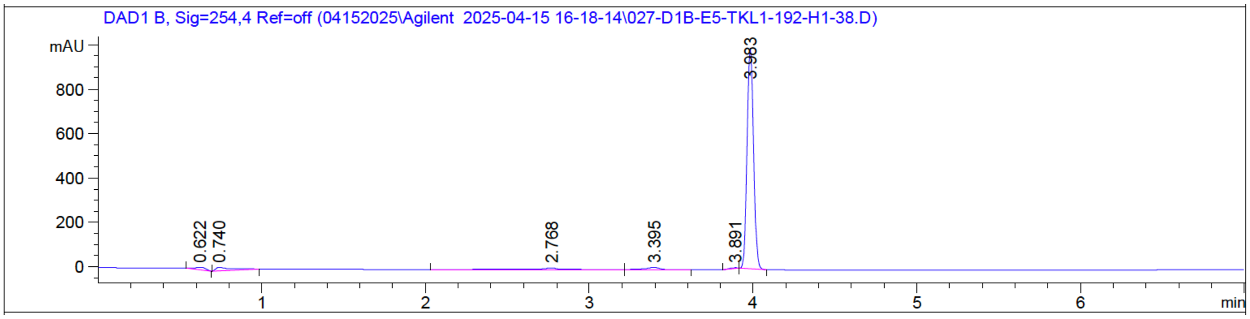

Supplementary Fig. 60 | LCMS spectrum for compound 36

UNC12567 (38)

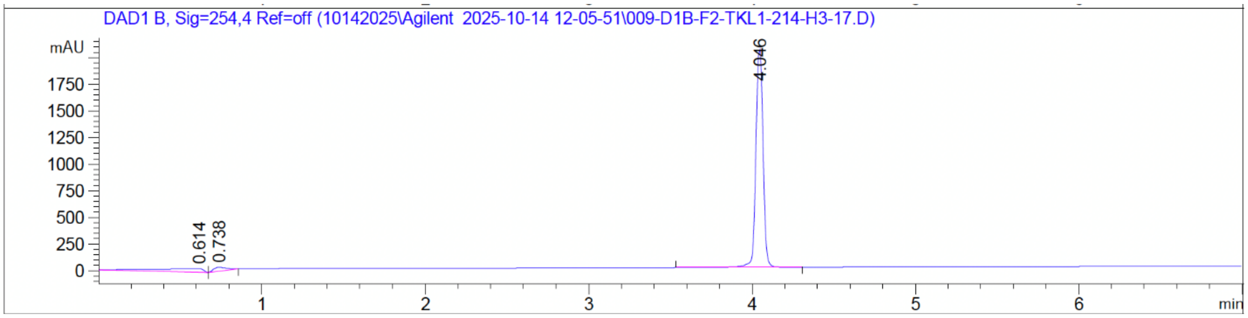

Supplementary Fig. 61 | LCMS spectrum for compound 38

**Reliability and reproducibility checklist for molecular dynamics simulations**<br>***All boxes must be marked YES by acceptance unless "Response not needed if No".**	**Yes**	**No**	**Response**<br>**(Please state where this information can be found in the text)**
**1. Convergence of simulations and analysis**			
1a. Is an evaluation presented in the text to show that the property being measured has equilibrated in the simulations<br>(*e.g.* time-course analysis)?	☒	☐	We examined the effective harmonic force constants and observed that both k_{0P} and k_{0D} reached a value of 1.0 by the end of the GaMD equilibration, indicating that the simulations were equilibrated.
1b. Then, is it described in the text how simulations are split into equilibration and production runs and how much data were analyzed from production runs?	☒	☐	The GaMD simulations contain a 50 ns equilibration and five independent 500 ns production runs. The information could be found under section titled "Gaussian accelerated Molecular Dynamics (GaMD) simulations and Simulation Analysis" in the MATERIAL AND METHODS section.
1c. Are there at least 3 simulations per simulation condition with statistical analysis?	☒	☐	The GaMD production has five independent 500 ns production simulations. The information could be found in under section "Gaussian accelerated Molecular Dynamics (GaMD) simulations and Simulation Analysis".
1d. Is evidence provided in the text that the simulation results presented are independent of initial configuration?	☒	☐	Three independent 500-ns GaMD simulations with randomized initial atomic velocities were performed on the FBXO22, FBXO22+UNC10088, FBXO22+UNC10088+NSD2, and FBXO22+BACH1/2 complexes. These independent GaMD simulations provided similar results of the system dynamics.
**2. Connection to experiments**			
2a. Are calculations provided that can connect to experiments (*e.g.* loss or gain in function from mutagenesis, binding assays, NMR chemical shifts, J-couplings, SAXS curves, interaction distances or FRET distances, structure factors, diffusion coefficients, bulk modulus and other mechanical properties, *etc.*)?	☒	☐	The GaMD simulations of all the complexes performed are highly consistent with the experimental data. The corresponding information could be found under sections "*Proximity-induced degradation of NSD2 does not perturb FBOX22-dependent BACH1 degradation*" and "*Identification of UNC10415667, a benzaldehyde containing SD2 degrador*".
**3. Method choice**			
3a. Do simulations contain membranes, membrane proteins, intrinsically disordered proteins, glycans, nucleic acids, polymers, or cryptic ligand binding?	☒	☐	The simulations did not include membranes, these are explicit systems in a water box with NaCl. Detailed information can be found in the section titled "Gaussian accelerated Molecular Dynamics (GaMD) simulations and Simulation Analysis."

3b. Is it described in the text whether the accuracy of the chosen model(s) is sufficient to address the question(s) under investigation (e.g. all-atom vs. coarse-grained models, fixed charge vs. polarizable force fields, implicit vs. explicit solvent or membrane, force field and water model, etc.)?	☒	☐	GaMD has been widely used to explore the structures and dynamics of membrane proteins and was therefore applied in the current study. Further details are provided in the section titled "Gaussian accelerated Molecular Dynamics (GaMD) simulations and Simulation Analysis".
3c. Is the timescale of the event(s) under investigation beyond the brute-force MD simulation timescale in this study that enhanced sampling methods are needed?	☒	☐	Due to low sampling efficiency, brute-force MD is difficult to capture the slow dynamics of our systems. Therefore, GaMD enhanced sampling simulations were applied.
If **YES**, are the parameters and convergence criteria for the enhanced sampling method clearly stated?	☒	☐	Five GaMD simulation trajectories, each 500 ns in length, the RMSF profiles, key intermolecular distance distributions, and conformational clustering analyses monitored and compared across replicas. The consistency of fluctuation patterns, stabilization of interaction distances, and recurrence of dominant conformational clusters indicate that the system sufficiently sampled the relevant conformational space and reached stable behavior".
If **NO,** is the evidence provided in the text?	☐	☐	
**4. Code and reproducibility**			
4a. Is a table provided describing the system setup that includes simulation box dimensions, total number of atoms, total number of water molecules, salt concentration, lipid composition (number of molecules and type)?	☒	☐	The information is provided in the section titled "Gaussian accelerated Molecular Dynamics (GaMD) simulations and Simulation Analysis".
4b. Is it described in the text what simulation and analysis software and which versions are used?	☒	☐	The information is provided in the section titled "Gaussian accelerated Molecular Dynamics (GaMD) simulations and Simulation Analysis".
4c. Are other parameters for the system setup described in the text, such as protonation state, type of structural restraints if applied, nonbonded cutoff, thermostat and barostat, etc.?	☒	☐	The information is provided in the section titled "Gaussian accelerated Molecular Dynamics (GaMD) simulations and Simulation Analysis".
4d. Are initial coordinate and simulation input files and a coordinate file of the final output provided as supplementary files or in a public repository?	☒	☐	We have shared the "initial coordinate, simulation input files and combined imaged trajectory files" for GaMD simulations of the FBXO22, FBXO22+UNC10088, FBXO22+UNC10088+NSD2, and FBXO22+BACH1/2 complexes in the public repository Figshare.<br>The following are the links:<br>FBXO22:<br>https://doi.org/10.6084/m9.figshare.31301590<br><br>FBXO22+UNC10088:<br>https://doi.org/10.6084/m9.figshare.31298356

			FBXO22+UNC10088+NSD2: https://doi.org/10.6084/m9.figshare.31301545<br><br>FBXO22+BACH: https://doi.org/10.6084/m9.figshare.31298464
4e. Is there custom code or custom force field parameters?	☐	☒	Response not needed if **No**
If **YES**, are they provided as supplementary files or in a public repository?	☐	☐	
