# Supplementary material for: Structural basis of NSD2 degradation via targeted recruitment of SCF-FBXO22
Source: Nat Commun. 2026 Apr 23;17:5635. doi: 10.1038/s41467-026-72235-9 (PMC13316043; doi:10.1038/s41467-026-72235-9)
Supplement: Supplementary file 2 — Description of Additional Supplementary Files [file 41467_2026_72235_MOESM2_ESM.pdf]

Description of Additional Supplementary Files

File Name: Supplementary Movie 1

Description: This movie compares 500 ns molecular dynamics trajectories of FBXO22 simulated with and without NSD2. In the presence of NSD2, both UNC10088 and the Y390 remain structurally stable, exhibiting minimal reorientation throughout the simulation. In contrast, the absence of NSD2 results in pronounced conformational dynamics, characterized by substantial rotation of the tyrosine residue and large-amplitude motions of UNC10088. Notably, despite these enhanced fluctuations, Y390 gradually returns to a native-like orientation at ~250 ns.
