# Supplementary material for: Structural basis of NSD2 degradation via targeted recruitment of SCF-FBXO22
Source: Nat Commun. 2026 Apr 23;17:5635. doi: 10.1038/s41467-026-72235-9 (PMC13316043; doi:10.1038/s41467-026-72235-9)
Supplement: Supplementary file 5 — Transparent Peer Review file [file 41467_2026_72235_MOESM5_ESM.pdf]

nature portfolio

Peer Review File

Structural basis of NSD2 degradation via targeted recruitment of SCF-FBXO22

Corresponding Author: Dr Nicholas Brown

This file contains all reviewer reports in order by version, followed by all author rebuttals in order by version.

Version 0:

Reviewer comments:

Reviewer #1

(Remarks to the Author)
The authors present an update on previous work they published using FBXO22 for targeted degradation of NSD2 using their reversible-covalent compound. Using their cryoEM structure of the ternary complex, they were able to identify key residues for compound binding as well as protein-protein interactions. This allowed them to use SBDD to design a more potent compound, which demonstrated cooperative binding between FBXO22 and NSD2. With the improved compound, they showed that binding of UNC10415667 to FBXO22 and subsequent formation of the ternary complex with neosubstrate NSD2 did not hinder the endogenous activity of FBXO22, namely binding and degradation of BACH1. This suggested that FBXO22 could bind substrates on multiple faces to allow for degradation. This potentially has implications on other E3s used for TPD and the effects on the homeostasis of their endogenous substrates when treated with degraders, but additional structures for these proteins will be required.

The authors did extensive studies to understand the interactions in their cryoEM structure in order to allow for SAR studies of UNC10088. Based on the interactions observed in the structure, their designed mutants appeared appropriate. For the TR-FRET experiments, is any difference observed if the compounds are not pre-incubated with FBXO22?
Were any attempts made to look at the FBXO22 structure in the absence of a substrate to look at the arrangement of C326 and the Y390 loop?
There are several clashes observed near the ligand binding site in the cryoEM structures, as reflected in their high clash score. How are these impacting the conclusions that can be made about the compound binding and protein-protein interactions?
Do the authors feel that the reversibility of the covalent warhead is required, or could an irreversible warhead also work? Additional compound series are likely out of the scope of this publication, but would be interesting to see follow up done.
Longer linkers were not favored in the benzaldehyde series. Can the authors use the cryoEM structure to explain if this is due to sterics or the added flexibility?
BACH1 has been shown to be ubiquitinated by both FBXO22 and FBXL17 (refs: https://www.cell.com/cell/fulltext/S0092-8674(24)01322-9; https://www.sciencedirect.com/science/article/pii/S0092867424011887). It is not well understood whether treatment with hemin results in degradation through FBXO22 or FBXL17. As such, the data does not totally support the authors' conclusion that endogenous substrates and neosubstrates can be degraded simultaneously. A better control would be to inhibit/knockdown FBXL17 and see if BACH1 levels are then affected by treatment with UNC10415667. Additionally for the degradation studies of NSD2 and BACH1, a negative control compound that does not have binding to NSD2 or an irreversible FBXO22 binder would be useful to understand the impact of the NSD2 binding on BACH1 binding to FBXO22. Given the covalent nature of the molecules, selectivity of labeling FBXO22 would be useful. Additionally, given the cooperative nature of binding, are kinetics of UNC10415667 binding to FBXO22 improved in the presence of NSD2?

Reviewer #2

(Remarks to the Author)
The authors provide a timely follow-up to their 2023 and 2024 papers outlining the structural basis of NSD2 recruitment to FBXO22 via their reported degrader. Their structures describe a mode of action by which UNC10088 (and UNC10415667) induces ternary complex formation mediated not only by a covalent interaction between ligand and FBXO22, but also protein-protein interaction between NSD2 and FBXO22. This interaction is validated by a series of mutations that reduced

ternary complex formation as observed by ubiquitination assays, SEC, and TR-FRET. The authors demonstrate preliminary SAR around their degrader. Finally, the authors demonstrate that degrader engagement of FBXO22 does not inhibit FBXO22-mediated degradation of BACH1.

Overall the paper is well-written and the figures are clear. It will be of interest to the TPD/UPS community due to the novelty of the neosubstrate interface on FBXO22 and clear potential to further improve compound-induced degradation of NSD2 due to the structural enablement.

We believe the manuscript could be strengthened by addressing the following points:

Do you have data for any other re-introduced cysteine-only mutants for figure 2a, since there are 7 of them?

Notably in lines 164-184 and figure 2b-c, the mutants that interact directly with ligand drastically reduce ubiquitylation whereas the PPI-disrupting mutants only partially hinder it. Have you assessed double/triple mutants to further disrupt the PPI, with the hypothesis that multiple mutations would more fully hinder ubiquitylation? Also, if all the FBXO22 proteins have been purified, it would be fitting to see TR-FRET with the suite of mutant proteins. The hypothesis is that the TR-FRET would match the ubiquitylation assay, however, there is only a small assay window on the TR-FRET in this particular format.

I like the hypothesis that the addition of an aromatic ring would promote additional tyrosine interactions. However, your data, DSF (Fig 3g) nor cryoEM, support that, as you state in line 268. Upon inspection of these experimental maps there is no density associated with Tyr390 in either map; it seems to have been simply pushed out of the way of the ligand. However in map j221 it looks like a bit of density matches the placement of Glu294 in the 8UA3 structure. These are pretty low-resolution maps, as are the published structures/maps, so I appreciate the difficulty in modelling with any specificity. I think it would be very interesting to understand the conformational dynamics of the loop containing Tyr390. Have you performed any MD on the FBXO22-ligand conjugates to find a low-energy conformation(s) of the Tyr loop? It's not a bad model, but I do want to know what that tyrosine is doing and why you put it there. This may inform on two following points as well.

In Extended Data Fig 5b, R2 and R3 can also be on the other face of the benzaldehyde. Please correct/clarify. With that in mind, you may wish to re-interpret your SAR. Line 297-299, also with Ser389 per your model (unless it's in a different spot per the above feedback / MD), Line 304 – more likely L246 of NSD2, as major clashes would be introduced to N360.

It would be critical to see dose response of NSD2 degradation with UNC12149. It could be more potent.

In Line 316-317 does your model (and suggested MD) support substrate recruitment via the induced beta sheet from this disordered region? While BACH1 binds on a different face, it is the disordered strand directly preceeding Tyr390 that makes those contacts from FBXO22. If indeed there is a conformational change of FBXO22 Tyr390 to support ligand binding, your model demonstrates changes propagated through residues 372. Your data seems to support minimal interference, but does your model?

Minor points to address:

Please provide citations for lines 98-103

Figures 1f-g, very difficult to see the residues being highlighted. Perhaps remove the surface representation, since it does not add anything to the take-home of the figures. One could also zoom in to the ligand about 2-3 fold to focus the viewer on the interactions. Figure 2 is so much clearer, although again, you could zoom in 2 fold to really make the stick representations stand out.

Were all SEC experiments done in same buffer? Including reducing agent? What was the cryoEM buffer?

Reviewer #3

(Remarks to the Author)
I co-reviewed this manuscript with one of the reviewers who provided the listed reports. This is part of the Nature Communications initiative to facilitate training in peer review and to provide appropriate recognition for Early Career Researchers who co-review manuscripts.

Reviewer #4

(Remarks to the Author)
In the manuscript, Robertson and colleagues present a study uncovering the detailed molecular mechanisms underlying UNC10088-mediated targeted protein degradation (TPD) of NSD2. The authors solved the cryo-EM structure of SCFFBXO22 in the presence of NSD2 and the aldehyde derivative of UNC10088. Specifically, UNC10088 facilitates the binding of FBXO22 to NSD2, which acts as a neosubstrate. The optimized analog, UNC10415667, displayed improved activity without the need for bioactivation. Structural analysis suggested that both compounds induced cooperative binding between FBXO22 and NSD2. These results strongly indicate that the UNC10088 degrader recruits NSD2 to a distinct FBXO22 surface other than that observed in the FBXO22-BACH1 complex. This is a good example of how E3 could be harnessed for TPD without significantly affecting its activity toward its native substrates in vivo. Overall the data presented in the study is of high quality, well-controlled, and convincing. I support its publication in Nature Communications with specific

points below.

1. The authors used neddylated CUL1-RBX1 for ubiquitination assays. A SDS−PAGE gel for neddylated CUL1-RBX1 and its unmodified form should be provided and compared to show the efficiency of neddylation.
2. All cryo-EM structures were solved and analyzed for the unmodified SCF complex. While the neddylation is unlikely to remarkably alter the binding interface between protein and compound, this should be mentioned in the discussion.
3. Page 13, Ling 328, please define the two fragments here: “the long and short forms of NSD2”.
4. Please also indicate whether each subunit within the SCF-FBXO22 complex is full length or its truncated form.

Version 1:

Reviewer comments:

Reviewer #1

(Remarks to the Author)
I appreciate the work done by the authors to address my original concerns. The majority of them have been addressed with additional data, which I find to further support the conclusions made in the original manuscript. The added structure for FBXO22 and both NSD2 and BACH1 is very helpful for demonstrating binding of both neo- and endogenous substrates.

My only remaining comment is with the data in Figure 2B, specifically for the FBXO22(E169K) mutant. There does not appear to be much of a difference in the ubiquitination blot, maybe because of the time point. The TR-FRET in Extended Data Figure 3B with the NSD2 mutants is a bit more convincing, as it shows a quantitative change. Could this be done with the FBXO22 mutants as well?

Reviewer #2

(Remarks to the Author)
Congratulations on the improved manuscripts - all our comments have been addressed.

Reviewer #3

(Remarks to the Author)
I co-reviewed this manuscript with one of the reviewers who provided the listed reports. This is part of the Nature Communications initiative to facilitate training in peer review and to provide appropriate recognition for Early Career Researchers who co-review manuscripts.

Reviewer #4

(Remarks to the Author)
My concerns were addressed satisfactorily and I am ok with the revised manuscript. I support its publication in Nature Communications

Open Access This Peer Review File is licensed under a Creative Commons Attribution 4.0 International License, which permits use, sharing, adaptation, distribution and reproduction in any medium or format, as long as you give appropriate credit to the original author(s) and the source, provide a link to the Creative Commons license, and indicate if changes were made.
In cases where reviewers are anonymous, credit should be given to 'Anonymous Referee' and the source.
The images or other third party material in this Peer Review File are included in the article's Creative Commons license, unless indicated otherwise in a credit line to the material. If material is not included in the article's Creative Commons license and your intended use is not permitted by statutory regulation or exceeds the permitted use, you will need to obtain permission directly from the copyright holder.
To view a copy of this license, visit https://creativecommons.org/licenses/by/4.0/

Point-by-point responses in red:

General response to Reviewers: We thank the Reviewers for their careful assessment of our work and suggestions for improving our manuscript. We tried to address all your suggestions, both in terms of experiments and text.

Reviewer #1 (Remarks to the Author)

The authors present an update on previous work they published using FBXO22 for targeted degradation of NSD2 using their reversible-covalent compound. Using their cryoEM structure of the ternary complex, they were able to identify key residues for compound binding as well as protein-protein interactions. This allowed them to use SBDD to design a more potent compound, which demonstrated cooperative binding between FBXO22 and NSD2. With the improved compound, they showed that binding of UNC10415667 to FBXO22 and subsequent formation of the ternary complex with neosubstrate NSD2 did not hinder the endogenous activity of FBXO22, namely binding and degradation of BACH1. This suggested that FBXO22 could bind substrates on multiple faces to allow for degradation. This potentially has implications on other E3s used for TPD and the effects on the homeostasis of their endogenous substrates when treated with degraders, but additional structures for these proteins will be required.

We thank the Reviewer for their appreciation of our findings!

1. The authors did extensive studies to understand the interactions in their cryoEM structure in order to allow for SAR studies of UNC10088. Based on the interactions observed in the structure, their designed mutants appeared appropriate. For the TR-FRET experiments, is any difference observed if the compounds are not pre-incubated with FBXO22?

The TR-FRET experiment was performed without preincubation as suggested. Overall, there is a negligible difference between the two experiments. Similar to the original cooperativity experiment (Fig. 2H), the IC50 was found to be 101 nM and 108 nM with or without preincubation, respectively, and the α-factor was relatively unchanged (3.1 compared to our original value of 3.3). A figure with the TR-FRET data generated without preincubation of the compounds with FBXO22 is now added to Extended Data Fig 3E and additional text is added to lines 209-210.

2. Were any attempts made to look at the FBXO22 structure in the absence of a substrate to look at the arrangement of C326 and the Y390 loop?

We attempted to refine FBXO22 structures in the absence of NSD2 using a subfraction of particles where NSD2 was not bound. However, FBXO22 exhibited substantial flexibility in these particles, preventing reliable modeling of the C326 region and Y390 loop. The structures presented therefore represent the stabilized, NSD2-bound state.

3. There are several clashes observed near the ligand binding site in the cryoEM structures, as reflected in their high clash score. How are these impacting the conclusions that can be made about the compound binding and protein-protein interactions?

The remaining clashes in our refined model are minor and localized away from the ligand-substrate interface, arising from the inherent ambiguity in sidechain placement at this resolution (4-5 Å in this region). Importantly, none involve the compound itself, and our conclusions are strongly supported by orthogonal biochemical data: (1) cooperative binding measurements (Fig. 2H), (2) mutagenesis of the NSD2-FBXO22 interface, and (3) ligand-dependent ternary complex formation assays (Fig. 2A-C, Extended Data Fig. 3A-C). These converging lines of evidence support both the ligand binding mode and the protein-protein interactions presented.

4. Do the authors feel that the reversibility of the covalent warhead is required, or could an irreversible warhead also work? Additional compound series are likely out of the scope of this publication, but would be interesting to see follow up done.

We agree with the Reviewer that including an additional compound series is outside the scope of this manuscript, but we appreciate the question and agree that this is an interesting line of investigation. Previously, we rationally designed, synthesized, and tested over 50 analogs of UNC8732 that contain irreversible warheads in place of the alkyl amine, and in each case, they do not seem to work in cell-free ubiquitination assays or cell-based assays to promote NSD2 ubiquitination or degradation, respectively. It is currently unclear why such compounds are not effective – possibility due to differences in size or polarity – but this is an area that we continue to pursue. Other groups have also pursued a similar line of investigation without any success (https://chemrxiv.org/doi/full/10.26434/chemrxiv-2025-wzgxk).

5. Longer linkers were not favored in the benzaldehyde series. Can the authors use the cryoEM structure to explain if this is due to sterics or the added flexibility?

We agree with the Reviewer that the linker length seems to be important, which is true for many bivalent degraders. In our example, we postulate that the additional protein-protein interactions and cooperative binding are likely sensitive to the linker length. Additional text was added to Lines 298-301.

6. BACH1 has been shown to be ubiquitinated by both FBXO22 and FBXL17 (refs: https://www.cell.com/cell/fulltext/S0092-8674(24)01322-9; https://www.sciencedirect.com/science/article/pii/S0092867424011887). It is not well understood whether treatment with hemin results in degradation through FBXO22 or FBXL17. As such, the data does not totally support the authors' conclusion that endogenous substrates and neosubstrates can be degraded simultaneously. A better control would be to inhibit/knockdown FBXL17 and see if BACH1 levels are then affected by treatment with UNC10415667. Additionally for the degradation studies of NSD2 and BACH1, a negative control compound that does not have binding to NSD2 or an irreversible FBXO22 binder would be useful to understand the impact of the NSD2 binding on BACH1 binding to FBXO22.

These are two very interesting points that we have made significant efforts to address. For the Reviewer's second point, we have performed experiments where we treat cells either with two different NSD2 degraders (UNC10415667 or UNC1088), or with a negative control compound that cannot bind to NSD2 (UNC12567). It is well precedented that conversion of the cyclopropyl group in the NSD2 ligand to an isopropyl group, as in UNC12567, essentially eliminates potent NSD2 binding, and this is further supported by a lack of ternary complex formation in the presence of UNC12567, monitored by size exclusion chromatography (Extended Data Figure 8J). Blotting for NSD2 showed that while both active degraders that can engage FBXO22 and NSD2 promote loss of NSD2, the negative control compound (UNC12567) had no effect on NSD2 levels. In addition, none of these molecules impacted the levels of BACH1, reported FBXO22 substrates (Figure 5E-F, Extended Data Figure 8K). The synthesis of UNC12567 has been added to the chemistry experimental methods.

The first point proved much more difficult to address with cell-based experiments. As the Reviewer suggested, we attempted to deplete FBXL17 and examine NSD2 and BACH1 levels in cells treated with the NSD2 degrader UNC10415667. Consistently, NSD2 was degraded in control and FBXL17 depleted cells treated with UNC10415667. However, BACH1 levels did not increase in that experiment. Moreover, the major band detected by our FBXL17 antibody, which has been used by several other groups, is not lost upon siRNA treatment. Thus, it is unclear that FBXL17 is in fact being depleted. We initially attempted to use a pair of siRNA oligonucleotide sequences previously reported in the literature to deplete FBXL17. In addition, we purchased a new set of pooled siRNAs from Horizon Discovery and obtained identical results. That is, BACH1 levels are unchanged by siRNA depletion of FBXL17. Further complicating this issue is our concern that the FBXL17 antibody may not be detecting endogenous levels of FBXL17, although it appears to pick up exogenously expressed versions, based on data by multiple other groups for this reagent. To the best of our knowledge, no peer reviewed publications have validated that the most commonly used antibody can detect endogenous protein.

Nevertheless, we have added other important data to support this point. First, we reanalyzed our MS data and found that there is no increase in BACH1 or KDM4A in UNC8732 treated cells (Lines 378-381). Similarly, a negative control compound has no effect on NSD2 or BACH1 levels when analyzed by western blot (Extended Data Figure 8K). Finally, we generated a new structure of SCF^{FBXO22} bound to both NSD2 and BACH1 simultaneously (Figure 5B-D, Extended Data Figure 8A-I). Altogether, this suggests different binding modes for BACH1 and NSD2 to FBXO22.

7. Given the covalent nature of the molecules, selectivity of labeling FBXO22 would be useful.

We previously attempted to show the labeling of FBXO22 by our compounds via mass spectrometry for our prior paper describing the discovery of our degraders and their mechanism of action (Nie et al., Nat Chem Bio 2024). However, the reversible nature of the covalent interaction made this difficult, and we were unable to detect the covalent interaction by mass spectrometry. Therefore, it is very challenging to determine if the aldehyde adduct of our degraders is engaging other proteins in a similar fashion; however, the mutagenesis, binding, DSF, and structural data support the formation of a covalent adduct on FBXO22 at Cys326 (Figure 2A-D, Figure 3F-G, Extended Data 1C) and the reversible nature of the interaction suggests that it is less likely to lead to problematic off target effects.

8. Additionally, given the cooperative nature of binding, are kinetics of UNC10415667 binding to FBXO22 improved in the presence of NSD2?

We attempted to perform the inverse TR-FRET experiment to determine if ligand binding to FBXO22 was improved by the addition of NSD2. For this experiment, we generated a biotinylated version of a benzaldehyde-based FBXO22 ligand. First, our goal was to evaluate binding of this biotin-benzaldehyde ligand to FBXO22. However, upon incubation of the biotin-benzaldehyde ligand with His-SKP1-GST-FBXO22, we saw no significant increase in signal window for the assay with increasing concentrations of either component, suggesting that the interaction is not potent enough to detect in this assay (this normally requires low micromolar reversible binding). We tried conducting the assay with both an anti-GST Ulight and anti-His Ulight as the acceptor fluorophore. It is also conceivable that the donor and acceptor were not positioned close enough for sufficient energy transfer. In light of this result, we were unable to evaluate any changes in the presence of NSD2; however, we appreciate the Reviewer's suggestion and think this would have been a nice addition to the manuscript.

Reviewer #2 (Remarks to the Author)

The authors provide a timely follow-up to their 2023 and 2024 papers outlining the structural basis of NSD2 recruitment to FBXO22 via their reported degrader. Their structures describe a mode of action by which UNC10088 (and UNC10415667) induces ternary complex formation mediated not only by a covalent interaction between ligand and FBXO22, but also protein-protein interaction between NSD2 and FBXO22. This interaction is validated by a series of mutations that reduced ternary complex formation as observed by ubiquitination assays, SEC, and TR-FRET. The authors demonstrate preliminary SAR around their degrader. Finally, the authors demonstrate that degrader engagement of FBXO22 does not inhibit FBXO22-mediated degradation of BACH1.

Overall the paper is well-written and the figures are clear. It will be of interest to the TPD/UPS community due to the novelty of the neosubstrate interface on FBXO22 and clear potential to further improve compound-induced degradation of NSD2 due to the structural enablement.

We appreciate the Reviewer's positive evaluation of our manuscript!

We believe the manuscript could be strengthened by addressing the following points:

1. Do you have data for any other re-introduced cysteine-only mutants for figure 2a, since there are 7 of them?

We would like to thank the Reviewer for this suggestion as our new data further highlight the specificity of Cys326 engagement by the compounds. To supplement our ternary complex formation assays through size exclusion chromatograph (SEC), we made two additional FBXO22 FIST mutants that either contained C227:C228 only or C378 only. Importantly, these cysteines are on the surface and C227:C228 are even near the UNC10088 binding site. In contrast to the C326 only variant, neither of the other two variants induced ternary complex formation, further strengthening our results. These finding have been added to Extended Data Figure 1C and lines 161-166.

2. Notably in lines 164-184 and figure 2b-c, the mutants that interact directly with ligand

drastically reduce ubiquitylation whereas the PPI-disrupting mutants only partially hinder it. Have you assessed double/triple mutants to further disrupt the PPI, with the hypothesis that multiple mutations would more fully hinder ubiquitylation? Also, if all the FBXO22 proteins have been purified, it would be fitting to see TR-FRET with the suite of mutant proteins. The hypothesis is that the TR-FRET would match the ubiquitylation assay, however, there is only a small assay window on the TR-FRET in this particular format.

We appreciate the Reviewer's suggestion of trying to demonstrate the disruption of the protein-protein interaction between FBXO22 and NSD2 through a binding experiment, further supporting our conclusion. Indeed, we were able to biotinylate the FBXO22 FIST domain and perform a TR-FRET assay to monitor its binding to his-tagged NSD2 in the presence of UNC10088. In this ternary complex assay, we revealed that the association of these proteins were reduced by single point mutations at K253 of NSD2. Importantly, all three mutations at that this position (K253W, K253D, K253E) gave similar trends in the binding assay when compared to the NSD2 ubiquitination results (Extended Data Figure 3A-C, Lines 186-194). Because we saw this clear agreement with single mutations disrupting the FBXO22-NSD2 interaction, we didn't test additional double and triple mutants.

3. I like the hypothesis that the addition of an aromatic ring would promote additional tyrosine interactions. However, your data, DSF (Fig 3g) nor cryoEM, support that, as you state in line 268. Upon inspection of these experimental maps there is no density associated with Tyr390 in either map; it seems to have been simply pushed out of the way of the ligand. However in map j221 it looks like a bit of density matches the placement of Glu294 in the 8UA3 structure. These are pretty low-resolution maps, as are the published structures/maps, so I appreciate the difficulty in modelling with any specificity. I think it would be very interesting to understand the conformational dynamics of the loop containing Tyr390. Have you performed any MD on the FBXO22-ligand conjugates to find a low-energy conformation(s) of the Tyr loop? It's not a bad model, but I do want to know what that tyrosine is doing and why you put it there. This may inform on two following points as well.

To address the principal concern of the placement of Tyr390 and that loop, we collected additional cryo-EM data and performed MD simulations. We first repurified the SCF^{FBXO22}-$UNC10088^{C}$-NSD2 sample to increase NSD2 occupancy, collected ~46k additional movies and performed focused refinements to resolve this part of the map. These data allowed us to create a new model that cleanly resolves this loop and contains unambiguous density for Tyr390 near NSD2. This new workflow and Tyr390 density are shown Extended Data Figure 2 and Extended Data Figure 4A. This new model was then used in MD simulations in the presence or absence of NSD2. When NSD2 is not present, the simulations produced a range of loop and tyrosine positions. Upon the addition of NSD2, the Tyr remains relatively stable and is in agreement with the new density and model (Extended Data Figure 4B-C and Movie S1, Lines 226-237). Taken together, both the new structural data and MD simulations agree with the placement of the Tyr-containing loop as it is positioned away from Cys326. With these new models, several figures containing UNC10088 and UNC10415667 have been updated accordingly.

4. In Extended Data Fig 5b, R2 and R3 can also be on the other face of the benzaldehyde. Please correct/clarify. With that in mind, you may wish to re-interpret your SAR. Line 297-299, also with Ser389 per your model (unless it's in a different spot per the above feedback / MD), Line 304 – more likely L246 of NSD2, as major clashes would be introduced to N360.

We attempted to obtain structural data for the UNC12149 complex to definitively determine R2/R3 positioning but were unable to achieve sufficient resolution. Given the rotational ambiguity without this structure, we have added a panel showing the alternative R2/R3 orientation (Extended Data Fig. 6C) and revised our discussion to avoid specifying particular residue interactions (Lines 324-337). Structural characterization of this complex remains an important goal for future work.

5. It would be critical to see dose response of NSD2 degradation with UNC12149. It could be more potent.

To assess the potency of UNC12149 compared to UNC10088 and UNC10415667, we titrated UNC12149 into our HiBit cell line and determined a DC50 of 120 nM. Indeed, UNC12149 was ~4-fold more potent than UNC10415667 and ~10-fold more potent than UNC8732. This titration has been added as Figure 4H and Lines 318-321 in the text.

6. In Line 316-317 does your model (and suggested MD) support substrate recruitment via the induced beta sheet from this disordered region? While BACH1 binds on a different face, it is the disordered strand directly preceeding Tyr390 that makes those contacts from FBXO22. If indeed there is a conformational change of FBXO22 Tyr390 to support ligand binding, your model demonstrates changes propagated through residues 372. Your data seems to support minimal interference, but does your model?

Yes, our new data includes a more highly resolved loop when NSD2 is bound compared to a beta-sheet when BACH1 is bound. To support this conclusion, we solved a new cryo-EM structure of the SCF^{FBXO22} which shows both NSD2 and BACH1 bound simultaneously (Figure 5B-D, Extended Data Figure 8A-I). By resolving the FBXO22 BACH1-interacting region and the Tyr390-containing loop, both regions closely resembled the orientation of the prior structures, suggesting a minimal interference model of these binding events. Y390 was found in the same location as our SCF^{FBXO22}-$UNC10088^{C}$-NSD2 structure and the FBXO22 beta sheet was still induced to interact with BACH1. This model is further supported by MD simulations (Extended Data Figure 7A-C). This new map and model are now presented in Figure 5B-D and Extended Data Figure 8-I.

Minor points to address:

7. Please provide citations for lines 98-103.

The citation for our prior reports describing the original parent compounds, UNC8732 and UNC1008, is now added to the text as requested.

8. Figures 1f-g, very difficult to see the residues being highlighted. Perhaps remove the surface representation, since it does not add anything to the take-home of the figures. One could also zoom in to the ligand about 2-3 fold to focus the viewer on the interactions. Figure 2 is so much clearer, although again, you could zoom in 2 fold to really make the stick representations stand out.

We appreciate the Reviewer’s suggestions to improve the figures and have done so accordingly (Figure 1F-G).

9. Were all SEC experiments done in same buffer? Including reducing agent? What was the cryoEM buffer?

All SEC experiments, including those for structural studies, were performed in 20 mM HEPES pH 8, 200 mM NaCl, 1 mM DTT. This information has been added to the appropriate methods sections.

Reviewer #3 (Remarks to the Author)

I co-reviewed this manuscript with one of the reviewers who provided the listed reports. This is part of the Nature Communications initiative to facilitate training in peer review and to provide appropriate recognition for Early Career Researchers who co-review manuscripts.

Thank you for helping engage Early Career Researchers in this important process.

Reviewer #4 (Remarks to the Author):

In the manuscript, Robertson and colleagues present a study uncovering the detailed molecular mechanisms underlying UNC10088-mediated targeted protein degradation (TPD) of NSD2. The authors solved the cryo-EM structure of SCFFBXO22 in the presence of NSD2 and the aldehyde derivative of UNC10088. Specifically, UNC10088 facilitates the binding of FBXO22 to NSD2, which acts as a neosubstrate. The optimized analog, UNC10415667, displayed improved activity without the need for bioactivation. Structural analysis suggested that both compounds induced cooperative binding between FBXO22 and NSD2. These results strongly indicate that the UNC10088 degrader recruits NSD2 to a distinct FBXO22 surface other than that observed in the FBXO22-BACH1 complex. This is a good example of how E3 could be harnessed for TPD without significantly affecting its activity toward its native substrates in vivo. Overall the data presented in the study is of high quality, well-controlled, and convincing. I support its publication in Nature Communications with specific points below.

We thank the Reviewer for their supportive response!

1. The authors used neddylated CUL1-RBX1 for ubiquitination assays. A SDS–PAGE gel for neddylated CUL1-RBX1 and its unmodified form should be provided and compared to show the efficiency of neddylation.

As suggested, an Coomassie-stained SDS-PAGE gel of the neddylated Split-n-Coexpressed CUL1-RBX1 is compared to the unneddylated complex, revealing a near total shift to the neddylated form (Extended Data Figure 1A, bottom panel).

2. All cryo-EM structures were solved and analyzed for the unmodified SCF complex. While the neddylation is unlikely to remarkably alter the binding interface between protein and compound, this should be mentioned in the discussion.

We agree with the Reviewer that mentioning that these structures are of the unneddylated form of the SCF and neddylation could potentially alter the binding interfaces is worthwhile. Additional text is now added to the discussion, lines 400-403.

3. Page 13, Ling 328, please define the two fragments here: “the long and short forms of NSD2”.

We apologize for this oversight. We have now included domain maps in Figure 5E to show what domains are included in the 2 major isoforms of NSD2 discussed in our manuscript.

4. Please also indicate whether each subunit within the SCF-FBXO22 complex is full length or its truncated form.

All proteins used to form the SCF-FBXO22 complex for structural studies were full-length except for NSD2 and BACH1, which were only the PWWP1 domain and the BTB domain, respectively. These clarifications were added to the methods section.

Point-by-point responses in red:

Reviewer #1 (Remarks to the Author):

I appreciate the work done by the authors to address my original concerns. The majority of them have been addressed with additional data, which I find to further support the conclusions made in the original manuscript. The added structure for FBXO22 and both NSD2 and BACH1 is very helpful for demonstrating binding of both neo- and endogenous substrates.

My only remaining comment is with the data in Figure 2B, specifically for the FBXO22(E169K) mutant. There does not appear to be much of a difference in the ubiquitination blot, maybe because of the time point. The TR-FRET in Extended Data Figure 3B with the NSD2 mutants is a bit more convincing, as it shows a quantitative change. Could this be done with the FBXO22 mutants as well?

We would like to thank the reviewer for their thoughtful consideration of our manuscript. Their suggested experiment would further support the finding that FBXO22 and NSD2 directly interact. However, this point is already demonstrated by the cryo-EM structures, several mutants in Figure 2, and additional mutagenesis in Extended Data Figure 3. Furthermore, this experiment could not be done quickly with the material in hand. The TR-FRET experiment uses the FIST domain of FBXO22 rather than full-length FBXO22 that is used in the ubiquitination assays.

Reviewer #2 (Remarks to the Author):

Congratulations on the improved manuscripts - all our comments have been addressed.

We appreciate the reviewer's enthusiastic response!

Reviewer #3 (Remarks to the Author):

I co-reviewed this manuscript with one of the reviewers who provided the listed reports. This is part of the Nature Communications initiative to facilitate training in peer review and to provide appropriate recognition for Early Career Researchers who co-review manuscripts.

Thank you for supporting the training of Early Career Researchers.

Reviewer #4 (Remarks to the Author):

My concerns were addressed satisfactorily and I am ok with the revised manuscript. I support its publication in Nature Communications

We thank the reviewer for their support for publication!
